# Protein truncating variants in mitochondrial-related nuclear genes and the risk of chronic liver disease

**DOI:** 10.1186/s12916-024-03466-0

**Published:** 2024-06-11

**Authors:** Huangbo Yuan, Zhenqiu Liu, Mingyang Chen, Qiaoyi Xu, Yanfeng Jiang, Tiejun Zhang, Chen Suo, Xingdong Chen

**Affiliations:** 1 State Key Laboratory of Genetic Engineering, Human Phenome Institute, Zhangjiang Fudan International Innovation Center, and School of Life Sciences, Fudan University, No. 2005 Songhu Road, Shanghai, 200438 China; 2 Department of Epidemiology, School of Public Health, Fudan University, No. 130 Dongan Road, Shanghai, 200032 China; 3 Fudan University Taizhou Institute of Health Sciences, Taizhou, China; 4 National Clinical Research Center for Aging and Medicine, Huashan Hospital, Fudan University, Shanghai, China; 5 Yiwu Research Institute of Fudan University, Yiwu, China

**Keywords:** Protein truncating variant, Mitochondrial dysfunction, Liver-related biomarkers, Chronic liver disease, Genetic susceptibility

## Abstract

**Background:**

Mitochondrial (MT) dysfunction is a hallmark of liver diseases. However, the effects of functional variants such as protein truncating variants (PTVs) in MT-related genes on the risk of liver diseases have not been extensively explored.

**Methods:**

We extracted 60,928 PTVs across 2466 MT-related nucleus genes using whole-exome sequencing data obtained from 442,603 participants in the UK Biobank. We examined their associations with liver dysfunction that represented by the liver-related biomarkers and the risks of chronic liver diseases and liver-related mortality.

**Results:**

96.10% of the total participants carried at least one PTV. We identified 866 PTVs that were positively associated with liver dysfunction at the threshold of *P* value < 8.21e − 07. The coding genes of these PTVs were mainly enriched in pathways related to lipid, fatty acid, amino acid, and carbohydrate metabolisms. The 866 PTVs were presented in 1.07% (4721) of participants. Compared with participants who did not carry any of the PTVs, the carriers had a 5.33-fold (95% CI 4.15–6.85), 2.82-fold (1.69–4.72), and 4.41-fold (3.04–6.41) increased risk for fibrosis and cirrhosis of liver, liver cancer, and liver disease-related mortality, respectively. These adverse effects were consistent across subgroups based on age, sex, body mass index, smoking status, and presence of hypertension, diabetes, dyslipidemia, and metabolic syndrome.

**Conclusions:**

Our findings revealed a significant impact of PTVs in MT-related genes on liver disease risk, highlighting the importance of these variants in identifying populations at risk of liver diseases and facilitating early clinical interventions.

**Supplementary Information:**

The online version contains supplementary material available at 10.1186/s12916-024-03466-0.

## Background

Liver disease, along with its most severe complications such as liver fibrosis, cirrhosis, and liver cancer, contributes to global mortality [1–3]. As early-stage chronic liver disease progresses to its end-stage form, the resultant irreversible liver injury leads to an unfavorable prognosis [4]. Therefore, it is essential to investigate the genetic and environmental factors influencing liver diseases and to identify at risk populations, particularly those susceptible to end-stage chronic liver disease. This is crucial for reducing the mortality rates associated with liver disease.

Liver mitochondria play a crucial role in glucose, lipid, and protein metabolism, as well as in energy generation [5]. The maintenance of mitochondrial function depends on various nuclear gene-encoded proteins [6]. Alterations in these regulatory genes can result to either excessive activation or inhibition of mitochondrial function, leading to changes in mitochondrial dynamics, reduced fatty acid β-oxidation, and increased levels of reactive oxygen species [5, 7, 8]. These are recognized as notable hallmarks in various liver diseases. Kraja and colleagues have demonstrated that genetic variants in nuclear genes related to mitochondria can affect body fat content, insulin levels, and higher risks of metabolic diseases and cancer [9]. However, a comprehensive understanding of their impact on liver diseases remains limited.

Protein truncating variants (PTVs), known for their ability to disrupt transcription and produce truncated or absent proteins, often leading to function loss, have exhibited a notable influence on complex traits [10]. Individuals with PTVs can serve as excellent “natural experiments” for studying the phenotypic consequences of genetic functional alterations [10]. Researching changes in liver function and the occurrence of liver diseases among PTV carriers can provide valuable insights into the functionality of nuclear genes related to mitochondria and provide clues for drug target discovery and the identification of populations at risk for liver diseases.

In this study, we identified PTVs in nuclear genes linked to mitochondrial function that showed strong associations with liver-related biomarkers using whole-exome sequencing (WES) data from the UK Biobank. Furthermore, we demonstrated an increased risk of end-stage chronic liver disease and liver disease-related mortality among individuals with these PTVs.

## Methods

### Description of study population

We conducted a genetic association study using WES and phenotypic data from the UK Biobank [11]. The UK Biobank is a resource containing dense genomics, lifestyle, and phenotyping data from a cohort of 500,938 volunteers, aged 40–69 years at recruitment, across the UK. The participants in this study provided electronic signed consent, completed questionnaires on socio-demographic, lifestyle, and health-related factors, underwent physical assessments and medical evaluations, and contributed DNA samples for future analysis. During the recruitment phase from 2006 to 2010, blood samples were collected to evaluate the levels of liver-related biomarkers, including alanine transaminase (ALT), alkaline phosphatase (ALP), aspartate transaminase (AST), gamma-glutamyl transferase (GGT), total bilirubin (TBIL), and albumin (ALB), while also serving as genomic source material for subsequent extraction.

For this study, we selected the final 470 k WES release (*N* = 469,686) participants with available WES data under project application number 92718. The study population underwent a selection process that excluded individuals marked as had withdrawn research consent and self-reported non-Caucasian race (26,911 removed) and individuals with inconsistent survey gender and genetic gender (91 removed). Consequently, a total of 442,603 individuals were included in the study.

### WES quality control and PTV annotation

The UK Biobank final 470 k WES release was analyzed utilizing an updated OQFE protocol (https://dnanexus.gitbook.io/uk-biobank-rap/science-corner/whole-exome-sequencing-oqfe-protocol/protocol-for-processing-ukb-whole-exome-sequencing-data-sets) [12]. The resulting OQFE CRAMs were processed through DeepVariant (v0.10.0) for small variant calling to generate per-sample gVCFs. These gVCFs were then aggregated and joint-genotyped via GLnexus to create a consolidated multi-sample VCF (pVCF) for all samples, which was employed in this study. To maximize data utility and ease of use, variant calling and hard filtering of variants with inbreeding coefficient <  − 0.03 or without at least one variant genotype of DP ≥ 10, GQ ≥ 20 and, if heterozygous, AB ≥ 0.20 were applied. In this study, we retained biallelic variants with an average sequencing quality greater than 30 and a minimum average sequencing depth greater than 10. Variants were then annotated using VEP v.102 [13] and LOFTEE v.1.0.4 plugin [14]. LOFTEE identifies high confidence loss-of-function (LoF) variants through a multi-step filtering process. It eliminates low-confidence LoF variants by excluding those near transcript ends, affected by non-canonical splice sites, or ancestral states across primates, among other criteria. Through these evaluations, we identified reliable LoF variants (referred to as PTVs in this study) for subsequent analyses. We extracted variants that were predicted to be PTVs, flagged as “high confidence” by LOFTEE, and exhibited a minor allele frequency (MAF) of less than 1% for each canonical transcript (as defined in Ensembl). Finally, we identified 789,840 high-confidence predicted rare PTVs (minor allele frequency < 1%) within canonical transcripts of 19,423 genes.

### Selection of mitochondrial-related nucleus genes

We compiled a list of mitochondrial-related nucleus genes from four sources: (1) MitoCarta 3.0 [15], (2) Literature Lab (Acumenta Biotech), (3) MitoMiner 4.0 [16], and (4) MitoProteome Human Mitochondrial Protein Database (updated date: 1/18/2022) [17]. The genes obtained from the first three sources have been described in a previous study [9]. Briefly, we used two separate sets of human genes and mouse orthologous genes from MitoCarta. We identified 36 terms based on MeSH mitochondria using the Literature Lab, selected only genes with probability functional scores in the upper quartile for each term, and accepted genes that had cited more than 15 abstracts. Using MitoMiner, we identified MT-related genes with a MT-MitoMiner index > 0.70. We then identified additional MT-related genes using MitoProteome database. After integrating genes from all sources and removing duplicates, we finalized a set of 2622 MT-related genes that are present in the human nomenclature (see Additional file 1: Table S1).

We extracted PTVs in the exon regions of 2622 MT-related genes. PTVs with a genotype missing rate > 20% and Hardy–Weinberg equilibrium *P* < 10e − 5 were excluded.

### Genetic association analyses

We conducted a genetic association analysis using PLINK 2.0 [18] for the acquired MT-related PTVs and liver-related biomarkers. Those with more than three missing liver-related biomarkers were excluded in the analysis. Given the rarity of PTVs, we decided to use raw liver-related biomarker measurement values directly with the application of a linear model. The model was adjusted for age, sex, and the top 10 genetic principal components (PCs) as covariates. The corresponding significant PTV results, those with the smallest *P* value per trait, were included in the final list (*P* value < 0.05/60,928 = 8.21e − 07).

Among the PTVs showing significant associations, we specifically focused on the results indicating negative correlations with ALB and positive correlations with ALT, AP, AST, GGT, and TBIL.

### Enrichment analysis

We conducted enrichment analysis of the corresponding protein-coding genes of the significant PTVs. R packages *clusterProfiler* and *topGO* were used to perform enrichment analysis of gene ontology (GO) and Kyoto Encyclopedia of Genes and Genomes (KEGG) pathway [19, 20].

### Definition of liver disease and liver-related mortality

The baseline disease history of liver disease was determined by identifying ICD-10 descriptions containing “liver,” “hepatic,” or “hepatitis,” coupled with the diagnosis occurrence within 6 months of enrollment. For subsequent calculations of incidence rates and Cox regression analysis, participants with pre-existing liver disease were excluded from the analysis.

This study primarily reported on end-stage liver diseases, including fibrosis and cirrhosis of liver, liver cancer, and liver disease-related mortality. To identify the incidence of fibrosis and cirrhosis of liver, we used ICD-10 codes K74, K702, K703, K704, and K717. Liver cancer cases were defined using ICD-10 code C22, encompassing all liver and intrahepatic bile ducts cancers. Liver disease-related mortality was defined based on primary cause of death, using UKB data field 40,001, which includes any ICD-10 descriptions containing “liver,” “hepatic,” or “hepatitis.”

This study also reported on other liver diseases, such as non-alcoholic fatty liver disease (NAFLD), viral hepatitis, and autoimmune hepatitis, as secondary outcomes. NAFLD was defined as ICD-10 K76.0 (fatty [change of] liver, not elsewhere classified) and K75.8 (non-alcoholic steatohepatitis, other specified inflammatory liver diseases). Viral hepatitis was defined as ICD-10 B15–B19. Autoimmune hepatitis was defined as ICD-10 K75.4.

### Covariates

We gathered information on age at enrolment, sex, average annual household income, alcohol intake frequency, ever smoked, physical activity level, education deprivation score, body mass index (BMI), and disease history of hypertension, diabetes, dyslipidemia, and metabolic syndrome. Alcohol intake frequency was categorized as < once a week or ≥ once a week. Average annual household income was categorized into five groups: < £18,000, £18,000–30,999, £31,000–51,999, £52,000–100,000, and > £100,000. We quantified physical activity level using number of days per week with moderate physical activity lasting ≥ 10 min. Subsequently, the physical activity level was categorized into 0–1 day/week, 2–4 days/week, and 5–7 days/week. The physical activity level group was treated as a continuous variable (1, 2, 3) and included in the model. The education deprivation score is a composite index that measures the degree of deprivation related to education, skills, and training in a specific area. Hypertension was defined as having a prior diagnosis of primary hypertension before enrollment. Diabetes was identified by a random blood glucose concentration of 11.1 mmol/L or higher, a glycated hemoglobin (HbA1c) level of 6.5% (48 mmol/mol) or higher at enrollment, or a clinical diagnosis by a doctor. Dyslipidemia was identified as low HDL cholesterol as HDL < 1.29 mmol/mol for women or HDL < 1.04 mmol/mol for men, and elevated triglyceride levels as triglyceride levels ≥ 1.70 mmol/mol. Metabolic syndrome was defined as having 3 or more of the following characteristics: abdominal obesity, defined as waist circumference greater > 88 cm for women or > 102 cm for men, triglycerides ≥ 1.70 mmol/mol, HDL < 1.29 mmol/mol for women or HDL < 1.04 mmol/mol for men, systolic blood pressure (SBP) ≥ 130 mm Hg or diastolic blood pressure (DBP) ≥ 85 mm Hg, or a random blood glucose concentration of 5.56 mmol/L or higher [21].

### Statistical analyses

We implemented a linear regression model to examine the relationship between MT-related PTVs and liver-related biomarkers, which were rank-based inverse normalized and adjusted for age, sex, and the first ten PCs as covariates. To compare incidence rates between PTV carriers and non-carriers, we utilized Kaplan–Meier curves and assessed statistical significance with the log-rank test. Incidence rates of end-stage liver disease and liver disease-related mortality were calculated per 100,000 person-years for both groups. We used a Cox regression model to assess the impact of PTVs on the risk of end-stage liver disease and liver disease-related mortality. This model was adjusted for age, sex, alcohol intake frequency, smoking history, physical activity level, education deprivation score, household income, BMI, hypertension, diabetes, dyslipidemia, and metabolic syndrome.

The Cox model was further stratified by age (under 60 and 60 or older), sex, alcohol intake frequency, smoking status, BMI (under 30 and 30 or above), hypertension, diabetes, dyslipidemia, metabolic syndrome, and known chronic liver disease susceptibility genes. We selected two genetic variants, PNPLA3 rs738409 C/G and TM6SF2 rs58542926 C/T, as stratification factors due to their established association with liver diseases [22–24]. Hazard ratios (HR) and 95% confidence intervals (CI) were calculated to quantify the associations.

We conducted a sensitivity analysis restricting consideration to PTVs present in more than 10 individuals. Furthermore, we employed the rare variant trend test (RVTT) to assess the relationship between the burden of qualifying PTVs in this study and liver disease status [25]. Briefly, we summarized the counts of PTVs in cases and controls using a 2xI contingency table, and the classic Cochran-Armitage trend test was used to calculate the original *z*-score. We then applied a permutation-based approach to determine significance *P* values. Case–control labels were permuted 10,000 times to calculate *z*-scores for each permutation. *P* values equaled the ratio of permuted *z*-scores ≥ original *z*-score to total permutations. Additionally, we extracted putatively deleterious variants mapping to the genes harboring the significant PTVs detected in our analysis and performed the RVTT to evaluate whether a dosage effect relationship exists between the cumulative burden of deleterious variants in these genes and risk of liver disease.

All statistical tests and graphs were performed using the R software version 4.3.0. *P* values were two-tailed, with a significance threshold set at *P* value < 0.05, unless otherwise specified.

## Results

### Characteristics of study population

**Fig. 1:**
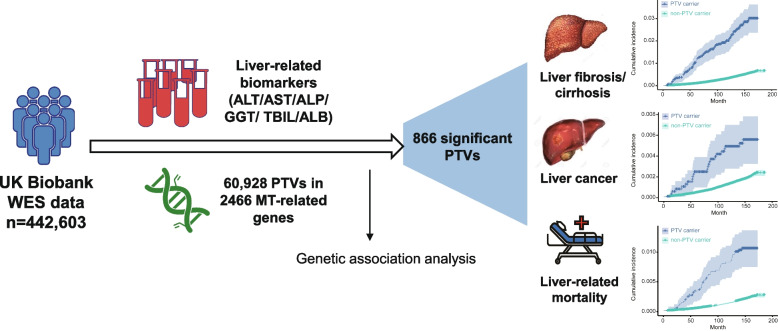
Study design. WES, whole-exome sequencing data; MT, mitochondrial; PTV, protein truncating variant; ALT, alanine aminotransferase; ALP, alkaline phosphatase; AST, aspartate aminotransferase; GGT, gamma-glutamyl transferase; TBIL, total bilirubin; ALB, albumin

Our final dataset included 442,603 self-reported white participants (54.27% female) with a mean age of 56.77 years (standard deviation = 8.03) from the UK Biobank (Fig. 1). A total of 60,928 PTVs across 2466 MT-related genes were extracted from these participants. A total of 96.10% (425,346) of participants carried at least one of these MT-related PTVs. The baseline characteristics of PTV carriers and non-carriers did not exhibit significant differences (Table 1).

**Table 1:** Baseline characteristics and liver-related outcomes of MT-related PTV carriers and non-carriers

Characteristic	MT-related PTVs		PTVs linked to liver biomarkers		
	Non-carriers (*n* = 17,257)	Carriers (*n* = 425,346)	Non-carriers (*n* = 437,882)	Carriers (*n* = 4721)	Multi-PTV carriers (*n* = 131)
Sex, *n* (%)					
Female	9320 (54.0)	230,877 (54.3)	237,872 (54.3)	2325 (49.2)^a^	43 (32.8)^ab^
Male	7937 (46.0)	194,469 (45.7)	200,010 (45.7)	2396 (50.8)	88 (67.2)
Age, *n* (%)					
< 60	9557 (55.4)	236,150 (55.5)	242,975 (55.5)	2732 (57.9)^a^	81 (61.8)
≥ 60	7700 (44.6)	189,196 (44.5)	194,907 (44.5)	1989 (42.1)	50 (38.2)
Alcohol intake frequency, *n* (%)					
< 1/week	12,324 (71.5)	302,990 (71.3)	311,945 (71.3)	3369 (71.5)	95 (72.5)
≥ 1/week	4921 (28.5)	122,055 (28.7)	125,630 (28.7)	1346 (28.5)	36 (27.5)
Ever smoked, *n* (%)					
No	6730 (39.1)	166,275 (39.2)	171,191 (39.2)	1814 (38.5)	42 (32.3)
Yes	10,476 (60.9)	257,603 (60.8)	265,184 (60.8)	2895 (61.5)	88 (67.7)
Body mass index (BMI), *n* (%)					
< 30	12,965 (75.4)	321,431 (75.8)	330,908 (75.8)	3488 (74.3)^a^	99 (75.6)
≥ 30	4232 (24.6)	102,539 (24.2)	105,562 (24.2)	1209 (25.7)	32 (24.4)
Physical activity level, *n* (%)					
0–1 day/week	3424 (20.9)	84,410 (20.8)	86,802 (20.8)	1032 (23.1)^a^	36 (28.3)^a^
2–4 days/week	6467 (39.4)	160,822 (39.7)	165,547 (39.7)	1742 (39.0)	39 (30.7)
5–7 days/week	6502 (39.7)	159,636 (39.4)	164,447 (39.5)	1691 (37.9)	52 (40.9)
Average annual household income, *n* (%)					
< £18,000	3233 (21.8)	81,522 (22.3)	83,839 (22.2)	916 (22.8)	37 (32.7)
£18,000–30,999	3840 (25.9)	93,240 (25.4)	96,053 (25.5)	1027 (25.5)	26 (23.0)
£31,000–51,999	3863 (26.0)	96,275 (26.3)	99,060 (26.3)	1078 (26.8)	26 (23.0)
£52,000–100,000	3102 (20.9)	75,310 (20.6)	77,618 (20.6)	794 (19.7)	19 (16.8)
> £100,000	805 (5.4)	20,031 (5.5)	20,628 (5.5)	208 (5.2)	5 (4.4)
Education deprivation score, median (P25–P75)	8.18 (2.54–19.12)	8.25 (2.70–19.42)	8.24 (2.70–19.40)	8.67 (2.90–20.57)^a^	10.15 (3.00–24.53)
Hypertension, *n* (%)					
No	12,818 (74.3)	317,498 (74.6)	326,907 (74.7)	3409 (72.2)^a^	92 (70.2)
Yes	4439 (25.7)	107,848 (25.4)	110,975 (25.3)	1312 (27.8)	39 (29.8)
Diabetes, *n* (%)					
No	16,291 (94.4)	402,089 (94.5)	413,986 (94.5)	4394 (93.1)^a^	118 (90.1)^a^
Yes	966 (5.6)	23,257 (5.5)	23,896 (5.5)	327 (6.9)	13 (9.9)
Dyslipidemia, *n* (%)					
No	7833 (50.1)	194,308 (50.4)	200,074 (50.5)	2067 (48.2)^a^	51 (41.1)^a^
Yes	7802 (49.9)	190,871 (49.6)	196,450 (49.5)	2223 (51.8)	73 (58.9)
Metabolic syndrome, *n* (%)					
No	12,914 (74.8)	319,387 (75.1)	328,846 (75.1)	3455 (73.2)^a^	87 (66.4)^a^
Yes	4343 (25.2)	105,959 (24.9)	109,036 (24.9)	1266 (26.8)	44 (33.6)
PNPLA3 rs738409, *n* (%)					
CC	10,720 (62.2)	261,778 (61.6)	269,616 (61.6)	2882 (61.1)	76 (58.0)
CG/GG	6517 (37.8)	163,066 (38.4)	167,745 (38.4)	1838 (38.9)	55 (42.0)
TM6SF2 rs58542926, *n* (%)					
CC	14,771 (85.6)	364,128 (85.6)	374,898 (85.6)	4001 (84.7)	109 (83.2)
CT/TT	2486 (14.4)	61,216 (14.4)	62,982 (14.4)	720 (15.3)	22 (16.8)
Liver-related outcome, *n* (%)					
NAFLD	225 (1.3)	5541 (1.3)	5631 (1.3)	135 (2.9)^a^	9 (6.9)^ab^
Viral hepatitis	34 (0.2)	899 (0.2)	897 (0.2)	36 (0.8)^a^	4 (3.1)^ab^
Autoimmune hepatitis	10 (0.1)	312 (0.1)	315 (0.1)	7 (0.1)	0 (0.0)
Fibrosis/cirrhosis	95 (0.6)	2437 (0.6)	2397 (0.5)	135 (2.9)^a^	18 (13.7)^ab^
Liver cancer	27 (0.2)	917 (0.2)	909 (0.2)	35 (0.7)^a^	4 (3.1)^ab^
Liver-related death	35 (0.2)	1088 (0.3)	1062 (0.2)	61 (1.3)^a^	7 (5.3)^ab^

^a^Statistical significance (*P* value < 0.05) when compared with PTV non-carriers; ^b^statistical significance (*P* value < 0.05) when compared with PTV carriers, chi-square test was used for comparison of categorical variables, and Kruskal–Wallis test was used for comparison of continuous variables. Abbreviations: *MT*, mitochondrial; *PTV*, protein truncating variant; *P25–P75*, first and third quartile; *NAFLD*, non-alcoholic fatty liver disease

### Association of MT-related PTVs with liver-related biomarkers

We identified 879 PTVs that were significantly associated with liver-related biomarkers. Among these PTVs, 866 exhibited positive correlations with liver injury severity, leading to elevated ALT/AST/ALP/GGT/TBIL levels or a reduction in ALB levels (Additional file 1: Table S2).

A total of 4721 individuals, representing 1.07% of the study population, were found to carry at least one of the 866 PTVs. Of these, 1.04% (4590 individuals) carried a single PTV, while 0.03% (131 individuals) carried two or more PTVs. All PTV carriers were heterozygous. Table 1 presents the baseline characteristics and liver-related outcomes of both PTV carriers and non-carriers. In comparison to non-carriers, PTV carriers exhibited significantly higher proportions of males (50.8% vs. 45.7%, *P* value < 0.001), obesity (25.7% vs. 24.2%, *P* value = 0.014), hypertension (27.8% vs. 25.3%, *P* value < 0.001), diabetes (6.9% vs. 5.5%, *P* value < 0.001), dyslipidemia (51.8% vs. 49.5%, *P* value = 0.003), and metabolic syndrome (26.8% vs. 24.9%, *P* value = 0.003). Additionally, PTV carriers had lower mean age and physical activity levels, as well as a higher mean education deprivation score. Regarding liver-related outcomes, in comparison to non-carriers, PTV carriers exhibited significantly higher proportions of NAFLD, viral hepatitis, liver fibrosis, liver cancer, and liver-related death. Notably, individuals carrying multiple PTVs had a significantly higher proportion of males, NAFLD, viral hepatitis, liver fibrosis, liver cancer, and liver-related death compared to all PTV carriers and non-carriers.

**Fig. 2:**
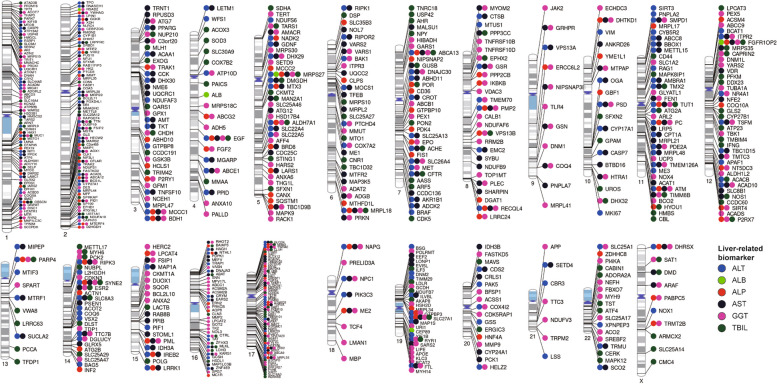
Chromosomal ideograms plot for coding genes of MT-related PTVs linked to liver-related biomarkers. An ideogram of all 22 chromosomes is plotted, along with the X chromosome. Lines are plotted on the chromosomes corresponding to the start location of each coding gene of PTV that was associated with liver-related biomarkers, and the line connects to colored circles representing the biomarkers associated with that PTV. Abbreviations: MT, mitochondrial; PTVs, protein truncating variants; ALT, alanine aminotransferase; ALP, alkaline phosphatase; AST, aspartate aminotransferase; GGT, gamma-glutamyl transferase; TBIL, total bilirubin; ALB, albumin

The 866 PTVs were identified across 661 genes, as depicted in Fig. 2. *TNN* displayed the highest number of significant associations in PTVs (11 PTVs), followed by *ATM* (8 PTVs), *VPS13B* (7 PTVs), *ABCA13* (6 PTVs), *FSIP2* (6 PTVs), *OBSCN* (5 PTVs), and *SYNE2* (5 PTVs). Notably, PTVs located within *MRPL18*, *SLC27A1*, *FGFR1OP2*, and *MRPS27* was associated with five liver-related biomarkers.

Analysis of the 866 PTVs revealed 228 with positive correlations to ALT, 258 to AST, 123 to ALP, 332 to GGT, 184 to TBIL, and 7 with negative correlations to ALB, as shown in Fig. 1. The overlap of PTVs among these biomarkers suggests underlying genetic connections. Specifically, 116 PTVs were common between ALT and AST, while 58, 49, and 47 PTVs were shared between GGT and AST, ALT, and ALP, respectively.

We treated the presence MT-related PTVs as binary variables and normalized the liver-related biomarkers. A linear regression model was then applied to assess the association between MT-PTVs and liver biomarker levels (Table 2). The findings demonstrated a significant increase in ALT, ALP, AST, GGT, and TBIL levels in individuals carrying MT-PTVs (*P* value < 0.001).

**Table 2:** Differences in liver-related biomarker levels between PTV carriers and non-carriers

Biomarker	PTV non-carriers median (P25–P75) (*n* = 437,882)	PTV carriers median (P25–P75) (*n* = 4721)	Beta	*P* value
ALT	20.13 (15.42–27.34)	22.28 (16.40–33.81)	0.30	< 0.001
AST	24.40 (21.00–28.80)	25.80 (21.70–32.80)	0.35	< 0.001
ALP	80.30 (67.20–95.70)	83.80 (69.00–102.20)	0.26	< 0.001
GGT	26.10 (18.40–40.70)	30.70 (20.50–58.48)	0.34	< 0.001
TBIL	8.08 (6.44–10.42)	8.395 (6.62–11.43)	0.16	< 0.001
ALB	45.21 (43.51–46.93)	45.42 (43.45–47.16)	0.02	0.555

The effect of PTVs on liver-related biomarkers was analyzed using linear regression. Age, sex, and the top 10 genetic principal components were included in the model as covariables. Abbreviations: *PTV* protein truncating variant, *P25–P75* first and third quartile, *ALT* alanine aminotransferase, *ALP* alkaline phosphatase, *AST* aspartate aminotransferase, *GGT* gamma-glutamyl transferase, *TBIL* total bilirubin, *ALB* albumin

### Enrichment analysis of significant PTVs

**Fig. 3:**
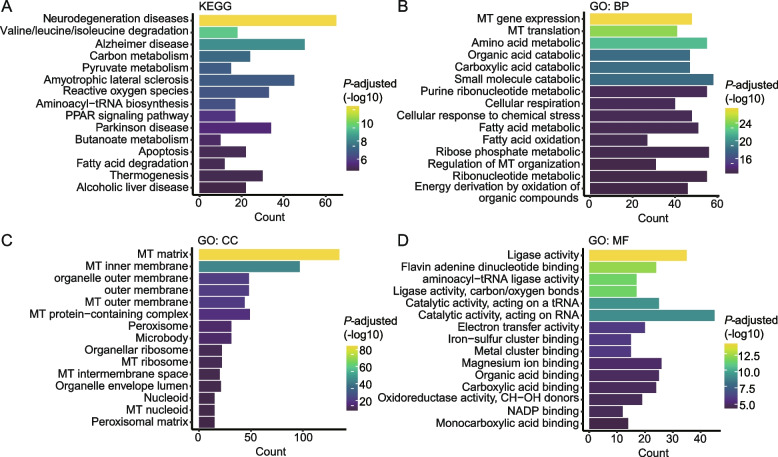
Functional enrichment analysis of PTVs linked to liver-related biomarkers. **A** The top 15 pathways of Kyoto Encyclopedia of Genes and Genomes pathway analysis (KEGG) based on PTVs linking to liver-related biomarkers. The top 15 terminologies of biological process (BP) (**B**), cellular component (CC) (**C**), and molecular function (MF) (**D**) in gene ontology (GO) analysis based on PTVs linked to liver-related biomarkers. Abbreviations: MT, mitochondrial; NADP, nicotinamide adenine dinucleotide phosphate

Genes harboring significant PTVs underwent comprehensive GO and KEGG enrichment analyses. The KEGG analysis indicated that these genes were predominantly involved in metabolic pathways, including those of lipids, fatty acids, amino acids, and carbohydrates, as well as reactive oxygen species, apoptosis, and pathways associated with neurodegenerative and alcoholic liver diseases (Fig. 3A). GO terms for biological processes (BP) were chiefly enriched in mitochondrial (MT) gene expression, organic acid catabolism, and various metabolic processes, alongside cellular respiration and energy derivation pathways (Fig. 3B). Cellular component (CC) terms were related to mitochondrial structure (Fig. 3C), and molecular function (MF) terms were linked to ligase activity (Fig. 3D). Furthermore, trait-specific enrichment analyses revealed distinct functional pathways for each trait, as detailed in Additional file 1: Fig. S1.

### Association between PTVs and the risk of chronic liver disease and liver-related mortality

**Fig. 4:**
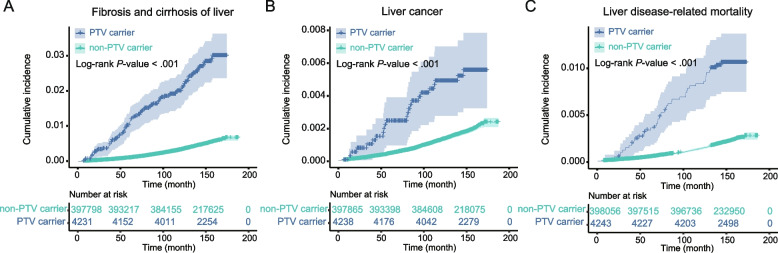
Cumulative incidences of liver diseases between mitochondrial-related PTV carriers and non-PTV carriers. Kaplan–Meier curves showing that mitochondrial-related PTV carriers had significantly increased risk of fibrosis and cirrhosis of liver (**A**), liver cancer (**B**), and liver disease-related mortality (**C**) when compared with non-PTV carriers

**Fig. 5:**
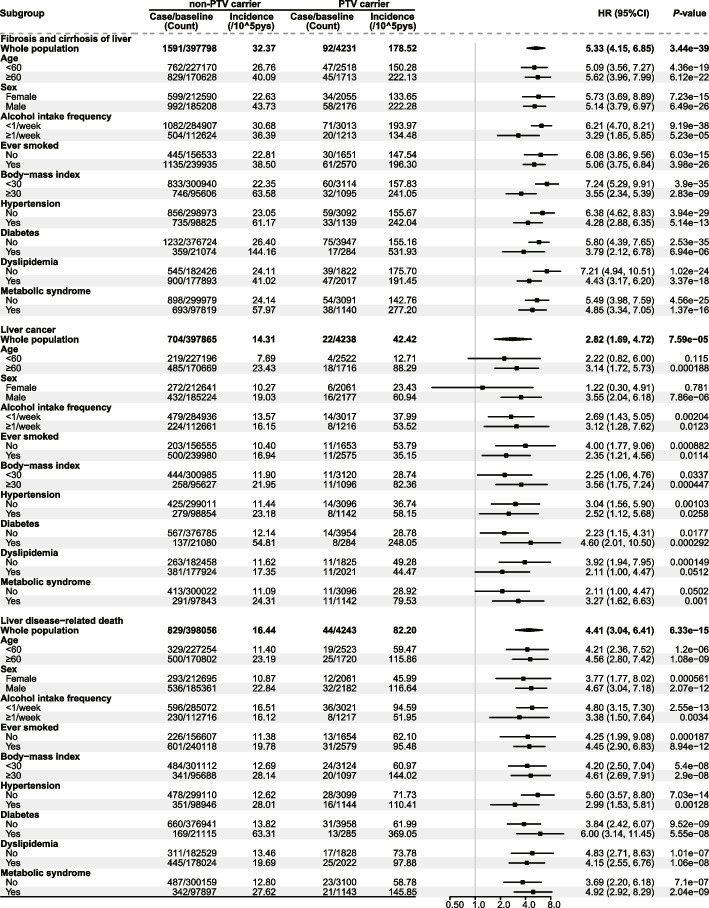
Impact of mitochondrial-related PTVs on the risks of end-stage liver diseases and liver-related mortality. The impact of PTVs on the risk of end-stage liver diseases (fibrosis and cirrhosis of liver and liver cancer) and liver disease-related mortality was evaluated using a COX regression model. The model was adjusted for age, sex, alcohol intake frequency, ever smoked, physical activity level, education deprivation score, household income, BMI, hypertension, diabetes, and dyslipidemia. The incidence rates (per 100,000 person-years, /10^5pys) of diseases were also calculated in the whole population and subgroups. Abbreviations: PTV, protein truncating variant; HR, hazard ratio; CI, confidence interval

After excluding participants with a history of liver disease and those with outcome events within 6 months of the survey, the remaining baseline population carrying PTVs were 4231 for liver fibrosis, 4238 for liver cancer, and 4243 for liver disease-related mortality. We observed a significantly increased risk of liver fibrosis (Fig. 4A), liver cancer (Fig. 4B), and liver disease-related mortality (Fig. 4C) in PTV carriers compared to non-carriers of PTVs. For liver fibrosis, the incidence rate was 178.52 per 100,000 person-years in PTV carriers, whereas for PTV non-carriers, it was 32.37 per 100,000 person-years. Similarly, the incidence rates of liver cancer and liver disease-related mortality were higher in PTV carriers, at 42.42 and 82.20 per 100,000 person-years, respectively, compared to 14.31 and 16.44 per 100,000 person-years in non-carriers (Fig. 5).

We used the Cox regression model to assess the impact of PTVs on the risk of end-stage liver disease and liver disease-related mortality (Fig. 5). The results revealed that PTVs were associated with a HR of 5.33 (95% CI 4.15–6.85) for liver fibrosis, 2.82 (95% CI 1.69–4.72) for liver cancer, and 4.41 (95% CI 3.04–6.41) for liver disease-related mortality.

The RVTT was used to assess the relationship between the burden of PTVs in this study and liver disease risks. Tendency of dose–response effects were observed for the effect of PTVs to liver fibrosis (original *z*-score =  − 22.35, *P* value < 0.001), liver cancer (original *z*-score =  − 6.93, *P* value < 0.001), and liver disease-related mortality (original *z*-score =  − 13.73, *P* value < 0.001). We subsequently conducted the RVTT on the 661 genes to evaluate the relationship between the cumulative burden of deleterious variants in genes and risk of liver-related outcomes. We identified *YWHAE* as significantly associated with liver fibrosis, *AVIL*, *FIS1*, *MUC1*, *ACOT7*, *CLUH*, *HNF4A*, and *TNFSF10* with liver cancer, and *AVIL* with liver disease-related mortality (FDR-adjusted *P* values < 0.05).

We further analyzed the association between liver injury biomarker-specific PTVs and pathway-specific PTVs with liver disease outcomes (Additional file 1: Fig. S2). Among liver injury biomarker-specific PTVs, PTVs in GGT significantly increased the risks of liver fibrosis (HR = 7.41, 95% CI 5.38–10.20), liver cancer (HR = 3.89, 95% CI 2.01–7.53), and liver disease-related mortality (HR = 5.47, 95% CI 3.33–9.00), while ALP PTVs substantially elevated the risk liver fibrosis (HR = 13.91, 95% CI 8.49–22.80) and liver disease-related mortality (HR = 5.83, 95% CI 2.18–15.59). For pathway-specific PTVs, those involved in butanoate metabolism (HR = 17.22, 95% CI 2.42–122.43), fatty acid degradation (HR = 18.31, 95% CI 2.57–130.35), and pyruvate metabolism (HR = 7.22, 95% CI 2.32–22.42) considerably increased the risk of liver fibrosis. PTVs in carbon metabolism (HR = 11.03, 95% CI 1.55–78.55) significantly increased the risk of liver cancer. PTVs in pathways the valine/leucine/isoleucine degradation (HR = 11.40, 95% CI 1.60–81.15), pyruvate metabolism (HR = 14.18, 95% CI 4.55–44.13), PPAR signaling pathway (HR = 7.91, 95% CI 1.97–31.74), and carbon metabolism (HR = 7.75, 95% CI 1.09–55.15) significantly increased the risk of liver disease mortality.

In addition, we examined the effects of PTVs on other liver diseases. Overall, PTVs significantly increased the risks of NAFLD (HR = 2.05, 95% CI 1.62–2.60) and viral hepatitis (HR = 2.10, 95% CI 1.04–4.23), while the result for autoimmune hepatitis was not significant (Additional file 1: Fig. S3). AST-specific PTVs (HR = 2.20, 95% CI 1.54–3.15) and GGT-specific PTVs (HR = 1.82, 95% CI 1.24–2.65) increased the risk of NAFLD, while ALT-specific (HR = 4.14, 95% CI 1.03–16.63) increased the risks of viral hepatitis. For pathway-specific PTVs, 5 pathway-specific PTVs considerably increased the risk of NAFLD.

### Sensitivity analysis

We performed a sensitivity analysis considering only PTVs present in more than 10 individuals. We found that 89 PTVs occurred in 10 or more individuals, with a total of 2859 individuals carrying the 89 PTVs. These PTVs significantly increased the risks of liver fibrosis (HR = 2.11, 95% CI 1.34–3.32), liver cancer (HR = 2.16, 95% CI 1.19–3.91), and liver disease-related mortality (HR = 2.10, 95% CI 1.12–3.92).

### Stratification analysis

To investigate the influence of PTVs on the risk of end-stage chronic liver disease and liver disease-related mortality across various subgroups, we conducted a comprehensive stratification analysis. This analysis was based on factors such as age, sex, alcohol consumption, smoking, BMI, hypertension, diabetes, dyslipidemia and metabolic syndrome (Fig. 5), and the presence of PNPLA3 rs738409/TM6SF2 rs58542926 variants (Additional file 1: Fig. S4). The results indicated that PTVs significantly increased the risk of all three liver disease outcomes across different factor groups. Specifically, PTVs significantly elevated the risk of liver disease in elders, males, smokers, those with a BMI ≥ 30, those with hypertension/diabetes/metabolic syndrome, and individuals carrying rs738409/rs58542926 variants.

Interaction analyses revealed significant interactions between obesity (coefficient =  − 0.71, *P* value = 0.008) with PTVs in the context of liver fibrosis. In non-obese individuals, PTVs conferred a 7.24-fold (95% CI 5.29–9.91) increased risk of liver fibrosis, while in obese individuals, the risk increase was 3.55-fold (95% CI 2.34–5.39).

## Discussions

To the best of our knowledge, this is the first large-scale cohort study to investigate the relationship between MT-related PTVs and liver-related biomarkers, and their impact on liver disease risk. Our findings establish a significant correlation between MT-related PTVs and the occurrence of end-stage chronic liver disease and liver disease-related mortality. These results highlight the crucial role of mitochondrial dysfunction in liver injury and disease susceptibility and emphasize the clinical significance of MT-related PTVs in assessing liver disease risk.

Previous studies have reported that genetic variants influencing liver-related biomarkers tend to cluster in pathways related to lipid and carbohydrate metabolism and were associated with intrahepatic and extrahepatic diseases [26–28]. Our study confirms these findings. Additionally, we identified PTVs that clustered in pathways associated with neurodegeneration diseases, organic acid catabolic, cellular respiration, energy derivation pathways, and mitochondrial composition. These pathways participate in various physiological processes in the liver [29–31]. Abnormal expression of their related proteins may cause abnormal liver function and liver injury, leading to abnormal expression of liver-related biomarkers. They may play important roles in the pathogenesis of liver diseases. The analysis of pathway-specific PTVs and their association with liver diseases revealed a significant increase in the risk of liver fibrosis attributed to PTVs in butanoate metabolism, fatty acid degradation, and pyruvate metabolism pathways. PTVs related to carbon metabolism increased the risk of liver cancer. PTVs related to butanoate metabolism, fatty acid degradation, and pyruvate metabolism prominently increased liver-related mortality risks. Our findings suggested the significant role of these pathways and genes in liver function and the development of liver disease.

A previous study reported associations between genetic variations in nuclear genes related to mitochondrial function and body mass index, waist-to-hip ratio, extreme obesity, blood glucose, insulin, and glycated hemoglobin levels [9]. As is well known, these factors are important risk factors for liver diseases. However, impacts on liver disease were not assessed in the study. Here, we demonstrated that MT-related PTV carriers have heightened chronic liver disease risk. We also found that the prevalence of metabolic diseases was significantly higher in PTV carriers compared to non-carriers. We speculate that individuals carrying MT-related PTVs may have impaired mitochondrial function or reduced adaptability to metabolic changes, rendering them more susceptible to metabolic disease, as well as to chronic liver disease. Metabolic abnormalities such as changes in fatty acid and glucose metabolism exert continuous pressure on liver mitochondria in the early stages of liver injury [5]. Persistent disruption in liver metabolism leads to chronic mitochondrial dysfunction and chronic liver diseases, eventually progressing to end-stage liver disease [5]. Individuals caring PTVs concurrently with obesity, diabetes, or metabolic syndrome displayed markedly increased liver disease and mortality risk, underscoring the vulnerability of metabolic homeostasis in this population and reveals potential mechanisms underlying their susceptibility to liver disease.

A negative interaction was observed between MT-related PTVs and obesity in relation to liver fibrosis risk. For metabolic abnormalities such as obesity, one of the major causes leading to liver disease is chronic mitochondrial dysfunction [32]. Our finding suggests that the presence of MT-related PTVs may predispose individuals to mitochondrial dysfunction, which may mask or override the incremental risk typically associated with obesity. The mitochondrial dysfunction in individuals carrying PTVs may have already reached a threshold of mitochondrial burden, wherein additional metabolic stressors, such as obesity, do not proportionally increase the risk of liver fibrosis. These findings confirm the role of mitochondrial dysfunction in liver disease mechanisms triggered by diverse environmental factors [5]. Notably, in individuals with inherent mitochondrial dysfunction (those with MT-related PTVs), various risk factors showed distinct effects, as demonstrated in this study, emphasizing the importance of lifestyle changes and early interventions within this high-risk group. Healthcare providers should be aware that traditional risk factors, such as obesity, may not exert the same degree of impact on the progression of liver disease in individuals carrying PTVs. Therefore, addressing other modifiable factors becomes even more crucial in formulating preventive strategies for this high-risk population.

This study has some limitations. First, the study population was exclusively European Caucasians in the UK Biobank, which may introduce the potential for population-specific heterogeneity of PTVs. Although we found that these PTVs were prevalent (1.48 to 11.04%) in other populations in UK Biobank, the sample size and case number of liver disease in this population limited validating the results in this study. Therefore, validation through large-scale cohorts with diverse populations is warranted in future research. Second, a higher percentage of PTVs associated with liver biomarkers was observed in males, potentially influencing the results. We propose that unique genetic predispositions in males, possibly affected by hormonal or environmental factors, may account for this discrepancy. In addition, the lack of long-term follow-up information in the study limits the understanding of disease progression, but our findings provide clues that the PTVs may have unfavorable effects on liver disease progression because these genetic variants have a broad impact on a spectrum of liver diseases from NAFLD to liver cancer. Lastly, given that the PTVs in this study are novel rare variants, applying eQTL and pQTL analyses to clarify the effects of these variants on downstream molecular expression presents a challenge. Consequently, the findings of this study necessitate future experimental validation, as well as investigation through additional large-scale cohorts.

## Conclusions

Our analysis provides compelling evidence for the association between MT-related PTVs and liver-related biomarkers, suggesting that these PTVs could serve as significant indicators for liver disease risk stratification. Integrating the knowledge of PTVs identified in this study into clinical practice can enhance risk assessment, early detection, and personalized management of liver diseases, ultimately improving patient prognosis. However, further research is required to confirm these results in diverse populations and to clarify the mechanisms by which PTVs contribute to liver damage.

### Supplementary Information

Additional file 1: Table S1 List of mitochondrial-related nuclear genes in the study. Table S2 Mitochondrial-related protein truncating variants that were positively associated with liver dysfunction. Fig. S1 Functional enrichment analysis of significantly associated PTVs for each liver-related biomarker. Fig. S2 Impact of biomarker-specific and pathway-specific PTVs on the risks of end-stage liver diseases and liver-related mortality. Fig. S3 Impact of PTVs on the risks of non-alcoholic fatty liver disease, viral hepatitis, and autoimmune hepatitis. Fig. S4 Stratified analysis of the impact of PTVs on mitochondrial-related PTVs on the risks of end-stage liver diseases and liver-related mortality based on liver disease susceptibility genes.

## Data Availability

All UK Biobank information is available online on the webpage www.ukbiobank.ac.uk/. Data access is available through applications. This research was conducted using the UK Biobank Resource under application number 92718.

## Abbreviations

ALB: Albumin
ALP: Alkaline phosphatase
ALT: Alanine aminotransferase
AST: Aspartate aminotransferase
BMI: Body mass index
BP: Biological process
CC: Cellular component
CI: Confidence intervals
GGT: Gamma-glutamyl transferase
GO: Gene ontology
HR: Hazard ratios
KEGG: Kyoto Encyclopedia of Genes and Genomes
MF: Molecular function
MT: Mitochondrial
PTV: Protein truncating variant
TBIL: Total bilirubin
WES: Whole-exome sequencing
