# Supplementary material for: Protein truncating variants in mitochondrial-related nuclear genes and the risk of chronic liver disease
Source: BMC Med. 2024 Jun 11;22:239. doi: 10.1186/s12916-024-03466-0 (PMC11167739; doi:10.1186/s12916-024-03466-0)

**SUPPLEMENTARY INFORMATION FOR**

**Protein truncating variants in mitochondrial-related nuclear genes and the risk of chronic liver disease**

*Yuan H et al.*

*TABLE OF CONTENT*

[**Table S1. List of mitochondrial-related nuclear genes in the study 3**](#_Toc168063208)

[**Table S2 Mitochondrial-related protein truncating variants that were positively associated with liver dysfunction 14**](#_Toc168063209)

[**Figure S1 Functional enrichment analysis of significantly associated PTVs for each liver-related biomarker. 62**](#_Toc168063210)

[**Figure S2 Impact of biomarker-specific and pathway-specific PTVs on the risks of end-stage liver diseases and liver-related mortality. 63**](#_Toc168063211)

[**Figure S3 Impact of PTVs on the risks of non-alcoholic fatty liver disease, viral hepatitis, and autoimmune hepatitis. 64**](#_Toc168063212)

[**Figure S4 Stratified analysis of the impact of PTVs on mitochondrial-related PTVs on the risks of end-stage liver diseases and liver-related mortality based on liver disease susceptibility genes. 65**](#_Toc168063213)

# Table S1. List of mitochondrial-related nuclear genes in the study

| **Gene Symbol** | | | | | |
| --- | --- | --- | --- | --- | --- |
| AADAT | CD34 | FKBP8 | MCUR1 | PCMT1 | SLC25A24 |
| AARD | CD36 | FLAD1 | MDH1 | PCNA | SLC25A25 |
| AARS2 | CD40 | FLCN | MDH2 | PDE12 | SLC25A26 |
| AASS | CD44 | FLVCR1 | MDM2 | PDE2A | SLC25A27 |
| ABAT | CD46 | FMC1 | ME1 | PDF | SLC25A28 |
| ABCA1 | CD68 | FNDC1 | ME2 | PDHA1 | SLC25A29 |
| ABCA12 | CD8A | FNDC5 | ME3 | PDHA2 | SLC25A3 |
| ABCA13 | CD93 | FOS | MECP2 | PDHB | SLC25A30 |
| ABCA8 | CDC25A | FOXD4L1 | MECR | PDHX | SLC25A31 |
| ABCA9 | CDC25C | FOXO1 | MED7 | PDIA3 | SLC25A32 |
| ABCB1 | CDH1 | FOXO3 | MEF2A | PDK1 | SLC25A33 |
| ABCB10 | CDIPT | FOXRED1 | MEF2C | PDK2 | SLC25A34 |
| ABCB6 | CDK1 | FPGS | MEFV | PDK3 | SLC25A35 |
| ABCB7 | CDK11B | FSIP1 | MET | PDK4 | SLC25A36 |
| ABCB8 | CDK2 | FSIP2 | METAP1D | PDP1 | SLC25A37 |
| ABCB9 | CDK4 | FTH1 | METTL15 | PDP2 | SLC25A38 |
| ABCC1 | CDK5 | FTL | METTL17 | PDPK1 | SLC25A39 |
| ABCC12 | CDK5RAP1 | FTMT | METTL5 | PDPR | SLC25A4 |
| ABCC13 | CDK6 | FUNDC1 | METTL8 | PDSS1 | SLC25A40 |
| ABCC2 | CDK7 | FUNDC2 | METTL9 | PDSS2 | SLC25A41 |
| ABCC3 | CDKN1A | FXN | MFF | PDX1 | SLC25A42 |
| ABCC8 | CDKN1B | FYN | MFN1 | PECAM1 | SLC25A43 |
| ABCC9 | CDKN2A | G0S2 | MFN2 | PECR | SLC25A44 |
| ABCD1 | CDKN3 | G6PC1 | MGARP | PEMT | SLC25A45 |
| ABCD2 | CDS2 | G6PD | MGME1 | PERP | SLC25A46 |
| ABCD3 | CEBPA | GABARAP | MGST1 | PET100 | SLC25A47 |
| ABCE1 | CEBPB | GABARAPL1 | MGST3 | PET117 | SLC25A48 |
| ABCF2 | CEBPZOS | GABARAPL2 | MICOS10 | PEX1 | SLC25A5 |
| ABCG1 | CEND1 | GABARAPL3 | MICOS10-NBL1 | PEX11B | SLC25A51 |
| ABCG2 | CEP20 | GABPA | MICOS13 | PEX19 | SLC25A52 |
| ABHD10 | CEP89 | GABPB1 | MICU1 | PEX3 | SLC25A53 |
| ABHD11 | CERK | GADD45A | MICU2 | PEX5 | SLC25A6 |
| ABHD16A | CERT1 | GADD45GIP1 | MICU3 | PFDN2 | SLC25A6P2 |
| ABHD6 | CES2 | GAL | MIEF1 | PFDN4 | SLC26A4 |
| ABL1 | CFAP45 | GALK2 | MIEF2 | PFKFB3 | SLC27A1 |
| ACAA1 | CFAP91 | GAP43 | MIGA1 | PFKM | SLC27A2 |
| ACAA2 | CFL1 | GAPDH | MIGA2 | PGAM5 | SLC27A3 |
| ACACA | CFLAR | GAPDHP44 | MIMT1 | PGAP4 | SLC2A1 |
| ACACB | CFTR | GARS1 | MIPEP | PGC | SLC2A2 |
| ACAD10 | CHAT | GATB | MIR1-1 | PGD | SLC2A3 |
| ACAD11 | CHCHD1 | GATC | MIR1-2 | PGK1 | SLC2A4 |
| ACAD8 | CHCHD10 | GATD3 | MIR210 | PGR | SLC30A6 |
| ACAD9 | CHCHD2 | GATM | MIR23A | PGRMC2 | SLC30A9 |
| ACADL | CHCHD2P9 | GBF1 | MIR7-3HG | PGS1 | SLC35B3 |
| ACADM | CHCHD3 | GCAT | MIX23 | PHB1 | SLC35E1 |
| ACADS | CHCHD4 | GCDH | MKI67 | PHB2 | SLC35F6 |
| ACADSB | CHCHD5 | GCG | MLEC | PHKA1 | SLC37A4 |
| ACADVL | CHCHD6 | GCK | MLH1 | PHYH | SLC3A1 |
| ACAN | CHCHD7 | GCKR | MLKL | PHYHIPL | SLC44A1 |
| ACAT1 | CHDH | GCLC | MLLT11 | PHYKPL | SLC44A5 |
| ACAT2 | CHEK1 | GCLM | MLXIP | PI4K2A | SLC4A5 |
| ACBD3 | CHEK2 | GCSH | MLYCD | PI4KA | SLC6A3 |
| ACCS | CHKB | GDAP1 | MMAA | PI4KB | SLC8A1 |
| ACHE | CHMP2B | GDF5-AS1 | MMAB | PICK1 | SLC8A3 |
| ACLY | CHPF | GDNF | MMACHC | PID1 | SLC8B1 |
| ACO1 | CHPT1 | GDPD1 | MMADHC | PIDD1 | SLC9A1 |
| ACO2 | CHUK | GFAP | MMP2 | PIF1 | SLC9A3 |
| ACOD1 | CIAPIN1 | GFER | MMP9 | PIK3C3 | SLC9A6 |
| ACOT1 | CIBAR1 | GFM1 | MMUT | PIM1 | SLC9B2 |
| ACOT13 | CIDEA | GFM2 | MOAP1 | PIM2 | SLIRP |
| ACOT2 | CIMAP2 | GGCT | MOBP | PIN1 | SLIT3 |
| ACOT7 | CISD1 | GGNBP1 | MOCS1 | PIN4 | SMCP |
| ACOT8 | CISD2 | GGPS1 | MOGS | PINK1 | SMDT1 |
| ACOT9 | CISD3 | GHITM | MOS | PINX1 | SMIM12 |
| ACOX1 | CKB | GHRL | MPC1 | PISD | SMIM17 |
| ACOX3 | CKM | GIMAP5 | MPC1L | PITRM1 | SMIM20 |
| ACP6 | CKMT1A | GIMAP8 | MPC2 | PKLR | SMPD1 |
| ACSBG2 | CKMT1B | GJA1 | MPDU1 | PKM | SMPD2 |
| ACSF2 | CKMT2 | GJB1 | MPG | PLA2G15 | SNAP29 |
| ACSF3 | CLEC5A | GJB2 | MPO | PLA2G2A | SNCA |
| ACSL1 | CLIC1 | GJB3 | MPP7 | PLA2G4A | SNCB |
| ACSL3 | CLIC4 | GJB6 | MPST | PLA2G4B | SND1 |
| ACSL4 | CLK1 | GK | MPV17 | PLA2G6 | SNHG29 |
| ACSL5 | CLN3 | GK2 | MPV17L | PLD6 | SNN |
| ACSL6 | CLN8 | GK3 | MPV17L2 | PLEC | SNPH |
| ACSM1 | CLPB | GLDC | MPZ | PLEK | SOD1 |
| ACSM2A | CLPP | GLO1 | MRM1 | PLGRKT | SOD2 |
| ACSM2B | CLPS | GLOD4 | MRM2 | PLIN1 | SOD3 |
| ACSM3 | CLPX | GLOD5 | MRM3 | PLIN2 | SORD |
| ACSM4 | CLTC | GLRX | MRPL1 | PLIN5 | SOSTDC1 |
| ACSM5 | CLU | GLRX2 | MRPL10 | PLN | SOX10 |
| ACSM6 | CLUH | GLRX3 | MRPL11 | PLPBP | SOX4 |
| ACSS1 | CLYBL | GLRX5 | MRPL12 | PLS3 | SP1 |
| ACSS2 | CMC1 | GLS | MRPL13 | PLSCR3 | SP100 |
| ACSS3 | CMC2 | GLS2 | MRPL14 | PMAIP1 | SPACA6 |
| ACTB | CMC4 | GLUD1 | MRPL15 | PML | SPARC |
| ACTC1 | CMPK1 | GLUD2 | MRPL16 | PMP2 | SPART |
| ACTN1 | CMPK2 | GLUL | MRPL17 | PMP22 | SPAST |
| ACTR2 | CNP | GLYAT | MRPL18 | PMPCA | SPATA18 |
| ACYP2 | CNR1 | GLYATL1 | MRPL19 | PMPCB | SPATA19 |
| ADA | CNTROB | GLYATL2 | MRPL2 | PNKD | SPATA20 |
| ADAM12 | COA1 | GLYATL3 | MRPL20 | PNPLA2 | SPATA2L |
| ADAP2 | COA3 | GLYCTK | MRPL21 | PNPLA4 | SPCS2 |
| ADAT2 | COA4 | GM2A | MRPL22 | PNPLA7 | SPG7 |
| ADCK1 | COA5 | GMFB | MRPL23 | PNPLA8 | SPHKAP |
| ADCK2 | COA6 | GMPPB | MRPL24 | PNPO | SPNS1 |
| ADCK5 | COA7 | GNG5 | MRPL27 | PNPT1 | SPR |
| ADCY10 | COA8 | GNL3L | MRPL28 | POLB | SPRYD4 |
| ADGB | COASY | GNPAT | MRPL3 | POLD3 | SPSB3 |
| ADGRL2 | COL11A2 | GOLPH3 | MRPL30 | POLDIP2 | SPTAN1 |
| ADH1A | COL6A1 | GOT1 | MRPL32 | POLG | SPTLC2 |
| ADH1B | COMT | GOT2 | MRPL33 | POLG2 | SQOR |
| ADH1C | COMTD1 | GPAA1 | MRPL34 | POLR1G | SQSTM1 |
| ADH5 | COQ10A | GPAM | MRPL35 | POLRMT | SRBD1 |
| ADH6 | COQ10B | GPAT2 | MRPL36 | POMC | SRC |
| ADHFE1 | COQ2 | GPD1 | MRPL37 | PON2 | SREBF1 |
| ADIPOQ | COQ3 | GPD2 | MRPL38 | POP7 | SREBF2 |
| ADIPOR1 | COQ4 | GPER1 | MRPL39 | POR | SRI |
| ADIPOR2 | COQ5 | GPI | MRPL4 | POU5F1 | SRP19 |
| ADIRF | COQ6 | GPRC5C | MRPL40 | PPA1 | SRY |
| ADO | COQ7 | GPT | MRPL41 | PPA2 | SSBP1 |
| ADORA1 | COQ8A | GPT2 | MRPL42 | PPARA | ST20 |
| ADORA2A | COQ8B | GPX1 | MRPL43 | PPARD | STAP1 |
| ADPRS | COQ9 | GPX3 | MRPL44 | PPARG | STAR |
| ADRB3 | CORO6 | GPX4 | MRPL45 | PPARGC1A | STARD13 |
| AFF4 | COX10 | GRAMD4 | MRPL46 | PPARGC1B | STARD3 |
| AFG1L | COX11 | GRHPR | MRPL47 | PPIA | STARD7 |
| AFG2A | COX14 | GRID1 | MRPL48 | PPIB | STAT1 |
| AFG3L1P | COX15 | GRN | MRPL49 | PPID | STAT3 |
| AFG3L2 | COX16 | GRPEL1 | MRPL50 | PPIF | STAT5A |
| AGAP2 | COX17 | GRPEL2 | MRPL51 | PPL | STAT5B |
| AGER | COX17P1 | GRSF1 | MRPL52 | PPM1E | STC1 |
| AGFG1 | COX18 | GSDMC | MRPL53 | PPM1K | STIM1 |
| AGK | COX19 | GSE1 | MRPL54 | PPM1M | STING1 |
| AGMAT | COX20 | GSK3A | MRPL55 | PPME1 | STK11 |
| AGPAT4 | COX4I1 | GSK3B | MRPL57 | PPOX | STOM |
| AGPAT5 | COX4I2 | GSN | MRPL58 | PPP1CC | STOML1 |
| AGPS | COX5A | GSR | MRPL9 | PPP1R12B | STOML2 |
| AGR2 | COX5B | GSS | MRPS10 | PPP1R14A | STX17 |
| AGRP | COX6A1 | GSTA4 | MRPS11 | PPP1R15A | STXBP1 |
| AGT | COX6A2 | GSTK1 | MRPS12 | PPP1R27 | SUCLA2 |
| AGTPBP1 | COX6B1 | GSTO1 | MRPS14 | PPP2CA | SUCLG1 |
| AGXT | COX6B2 | GSTP1 | MRPS15 | PPP2CB | SUCLG2 |
| AGXT2 | COX6C | GSTZ1 | MRPS16 | PPP2R1A | SUGCT |
| AHCYL1 | COX7A1 | GTPBP10 | MRPS17 | PPP2R2B | SUOX |
| AHR | COX7A2 | GTPBP3 | MRPS18A | PPP2R3C | SUPV3L1 |
| AIF1 | COX7A2L | GTPBP6 | MRPS18B | PPP3CA | SURF1 |
| AIFM1 | COX7A2P2 | GTPBP8 | MRPS18C | PPP3CC | SYBU |
| AIFM2 | COX7B | GUF1 | MRPS2 | PPP3R1 | SYNE2 |
| AIFM3 | COX7B2 | GUK1 | MRPS21 | PPRC1 | SYNJ2BP |
| AIM2 | COX7C | GUSB | MRPS22 | PPTC7 | SYNJ2BP-COX16 |
| AIP | COX8A | GZMB | MRPS23 | PPWD1 | SYP |
| AIPL1 | COX8C | H2AX | MRPS24 | PRDM16 | TACO1 |
| AK1 | CP | H6PD | MRPS25 | PRDX1 | TAF3 |
| AK2 | CPOX | HAAO | MRPS26 | PRDX2 | TAFAZZIN |
| AK3 | CPS1 | HADH | MRPS27 | PRDX3 | TAL2 |
| AK4 | CPT1A | HADHA | MRPS28 | PRDX4 | TALDO1 |
| AKAP1 | CPT1B | HADHB | MRPS30 | PRDX5 | TAMM41 |
| AKAP10 | CPT1C | HAGH | MRPS31 | PRDX6 | TANC2 |
| AKAP8 | CPT2 | HAMP | MRPS33 | PRELID1 | TANGO2 |
| AKR1A1 | CRAT | HAO2 | MRPS34 | PRELID2 | TAP1 |
| AKR1B1 | CRB1 | HAP1 | MRPS35 | PRELID3A | TARDBP |
| AKR1B10 | CREB1 | HARS2 | MRPS36 | PRELID3B | TARS1 |
| AKR1B15 | CRLS1 | HAVCR1 | MRPS5 | PREPL | TARS2 |
| AKR1C4 | CROT | HAX1 | MRPS6 | PRF1 | TAT |
| AKR7A2 | CRTC1 | HBB | MRPS7 | PRIMPOL | TATDN3 |
| AKT1 | CRTC2 | HCCS | MRPS9 | PRKAA1 | TBC1D15 |
| AKT2 | CRY1 | HCFC1 | MRRF | PRKAA2 | TBC1D32 |
| AKT3 | CRYAA | HCLS1 | MRS2 | PRKACA | TBC1D9B |
| ALAD | CRYAB | HDAC1 | MSRA | PRKCA | TBK1 |
| ALAS1 | CRYM | HDAC6 | MSRB2 | PRKCB | TBRG4 |
| ALAS2 | CRYZ | HDAC9 | MSRB3 | PRKCD | TCAIM |
| ALB | CS | HDDC2 | MSS51 | PRKCE | TCF3 |
| ALDH18A1 | CSDE1 | HDHD3 | MSTN | PRKCZ | TCF4 |
| ALDH1A1 | CSK | HDHD5 | MSTO1 | PRKDC | TCHP |
| ALDH1B1 | CSKMT | HEATR1 | MTCH1 | PRKN | TCIRG1 |
| ALDH1L1 | CTH | HEATR6 | MTCH2 | PRNP | TCN1 |
| ALDH1L2 | CTNNB1 | HEBP1 | MTCP1 | PRODH | TCN2 |
| ALDH2 | CTPS2 | HEBP2 | MTERF1 | PRODH2 | TCP1 |
| ALDH3A1 | CTRL | HECW2 | MTERF2 | PRORP | TDH |
| ALDH3A2 | CTSA | HELZ2 | MTERF3 | PRR5L | TDP1 |
| ALDH4A1 | CTSB | HEMK1 | MTERF4 | PRRX1 | TDRD7 |
| ALDH5A1 | CTSD | HERC2 | MTF1 | PRSS35 | TDRKH |
| ALDH6A1 | CTSL | HFE | MTFMT | PRXL2A | TEFM |
| ALDH7A1 | CTU1 | HGF | MTFP1 | PSAP | TERT |
| ALDH9A1 | CTU2 | HIBADH | MTFR1 | PSD | TEX47 |
| ALDOC | CUEDC1 | HIBCH | MTFR1L | PSEN1 | TF |
| ALKBH1 | CXADR | HIF1A | MTFR2 | PSEN2 | TFAM |
| ALKBH3 | CXCL8 | HIGD1A | MTG1 | PSMA6 | TFB1M |
| ALKBH7 | CYB5A | HIGD2A | MTG2 | PSMB3 | TFB2M |
| ALOX12 | CYB5B | HINT1 | MTHFD1 | PSMD10 | TFDP1 |
| ALOX15 | CYB5R1 | HINT2 | MTHFD1L | PSTK | TFEB |
| AMACR | CYB5R2 | HINT3 | MTHFD2 | PTCD1 | TFEC |
| AMBRA1 | CYB5R3 | HIVEP1 | MTHFD2L | PTCD2 | TFRC |
| AMELX | CYBA | HK1 | MTHFS | PTCD3 | TGFB1 |
| AMPD2 | CYBB | HK2 | MTIF2 | PTCHD4 | TGM2 |
| AMT | CYC1 | HK3 | MTIF3 | PTEN | TH |
| ANGEL2 | CYCS | HKDC1 | MTM1 | PTF1A | THAP3 |
| ANKRD26 | CYP11A1 | HLCS | MTO1 | PTGES2 | THEM4 |
| ANTKMT | CYP11B1 | HMBS | MTOR | PTGR3 | THEM5 |
| ANXA1 | CYP11B2 | HMGB1 | MTPAP | PTGS1 | THG1L |
| ANXA10 | CYP17A1 | HMGCL | MTR | PTGS2 | THNSL1 |
| ANXA2 | CYP1A1 | HMGCR | MTRES1 | PTH2 | THY1 |
| ANXA5 | CYP1A2 | HMGCS2 | MTRF1 | PTK2 | TIMM10 |
| ANXA6 | CYP1B1 | HMOX1 | MTRF1L | PTPMT1 | TIMM10B |
| AOX1 | CYP21A2 | HNF4A | MTRFR | PTPN1 | TIMM13 |
| AOX2P | CYP24A1 | HNRNPK | MTUS1 | PTPN11 | TIMM17A |
| AP2B1 | CYP27A1 | HOGA1 | MTX1 | PTPN4 | TIMM17B |
| AP2M1 | CYP27B1 | HOXB9 | MTX2 | PTRH1 | TIMM21 |
| APAF1 | CYP2D6 | HPDL | MTX3 | PTRH2 | TIMM22 |
| APBA1 | CYP2E1 | HRK | MUC1 | PTS | TIMM23 |
| APEX1 | CYP7A1 | HSCB | MUL1 | PUF60 | TIMM23B |
| APEX2 | D2HGDH | HSD17B10 | MUTYH | PUS1 | TIMM29 |
| APOE | DACT2 | HSD17B4 | MYC | PUS10 | TIMM44 |
| APOO | DAO | HSD17B8 | MYCBP | PUSL1 | TIMM50 |
| APOOL | DAOA | HSD3B1 | MYCN | PVALB | TIMM8A |
| APP | DAP | HSD3B2 | MYG1 | PWAR4 | TIMM8B |
| AQP8 | DAP3 | HSDL1 | MYH10 | PXMP2 | TIMM9 |
| AR | DARS1 | HSDL2 | MYH14 | PXMP4 | TIMMDC1 |
| ARAF | DARS2 | HSF1 | MYH2 | PYCARD | TK1 |
| ARF5 | DBI | HSH2D | MYH6 | PYCR1 | TK2 |
| ARG1 | DBT | HSP90AA1 | MYH9 | PYCR2 | TKT |
| ARG2 | DCAF5 | HSP90AB1 | MYL10 | PYGM | TLR4 |
| ARGLU1 | DCAKD | HSP90B1 | MYO19 | PYROXD2 | TLR9 |
| ARL2 | DCK | HSPA1A | MYO1C | PYURF | TM9SF4 |
| ARL2BP | DCPS | HSPA1L | MYO5A | QARS1 | TMBIM4 |
| ARL6IP5 | DCXR | HSPA2 | MYOC | QDPR | TMBIM6 |
| ARMC1 | DDAH1 | HSPA4 | MYOD1 | QRSL1 | TMEM101 |
| ARMC10 | DDAH2 | HSPA5 | MYOG | QTRT1 | TMEM102 |
| ARMCX1 | DDIT3 | HSPA8 | MYOM2 | QTRT2 | TMEM11 |
| ARMCX2 | DDIT4 | HSPA9 | NABP2 | RAB11A | TMEM126A |
| ARMCX3 | DDT | HSPB1 | NADK2 | RAB11B | TMEM126B |
| ARMS2 | DDX23 | HSPB2 | NAGS | RAB11FIP5 | TMEM141 |
| ARNT | DDX28 | HSPB7 | NAIF1 | RAB1B | TMEM143 |
| ARRB2 | DDX3X | HSPB8 | NAMPT | RAB24 | TMEM14C |
| ARSB | DECR1 | HSPD1 | NANOG | RAB29 | TMEM160 |
| AS3MT | DEFA5 | HSPE1 | NAPG | RAB32 | TMEM177 |
| ASAH1 | DEGS1 | HTATIP2 | NARS1 | RAB35 | TMEM186 |
| ASAH2 | DELE1 | HTD2 | NARS2 | RAB3A | TMEM205 |
| ASB9 | DENND2D | HTRA1 | NAT8L | RAB3D | TMEM223 |
| ASPDH | DEPP1 | HTRA2 | NAV2 | RAB40AL | TMEM256 |
| ASS1 | DEPTOR | HTT | NAXD | RAB5IF | TMEM65 |
| ATAD1 | DES | HYOU1 | NAXE | RAB7A | TMEM69 |
| ATAD3A | DFFA | IARS2 | NBR1 | RAB8B | TMEM70 |
| ATAD3B | DFFB | IBA57 | NCBP1 | RAC1 | TMEM8B |
| ATCAY | DGAT1 | ICAM1 | NCEH1 | RACK1 | TMLHE |
| ATF2 | DGAT2 | IDE | NCF1 | RAD51 | TMTC1 |
| ATF3 | DGLUCY | IDH1 | NCF2 | RAD51C | TMTC3 |
| ATF4 | DGUOK | IDH2 | NCOA4 | RAF1 | TMX2 |
| ATF6 | DHCR24 | IDH3A | NDFIP2 | RAG1 | TNF |
| ATG12 | DHFR | IDH3B | NDOR1 | RAG2 | TNFRSF10A |
| ATG16L1 | DHFR2 | IDH3G | NDUFA1 | RAI1 | TNFRSF10B |
| ATG2A | DHODH | IDI1 | NDUFA10 | RAI14 | TNFRSF10C |
| ATG2B | DHRS1 | IER3 | NDUFA11 | RAP1GDS1 | TNFRSF10D |
| ATG3 | DHRS2 | IER5L | NDUFA12 | RAPGEF3 | TNFRSF1A |
| ATG4A | DHRS3 | IFI27 | NDUFA13 | RAPGEF4 | TNFRSF1B |
| ATG4B | DHRS4 | IFI6 | NDUFA2 | RARA | TNFSF10 |
| ATG4C | DHRS7 | IFIH1 | NDUFA3 | RARS1 | TNFSF12 |
| ATG4D | DHRS7B | IFIT2 | NDUFA4 | RARS2 | TNNC1 |
| ATG5 | DHRSX | IFIT3 | NDUFA4L2 | RAVER1 | TNNT1 |
| ATG7 | DHTKD1 | IFNB1 | NDUFA5 | RB1 | TNRC18 |
| ATG9A | DHX29 | IFNG | NDUFA6 | RBFA | TOMM20 |
| ATIC | DHX30 | IFNG-AS1 | NDUFA7 | RBFOX3 | TOMM20L |
| ATM | DHX32 | IGF1 | NDUFA8 | RBP3 | TOMM22 |
| ATP10D | DHX57 | IGF1R | NDUFA9 | RCAN1 | TOMM34 |
| ATP13A2 | DIABLO | IGF2BP1 | NDUFAB1 | RCC1L | TOMM40 |
| ATP23 | DIAPH2 | IGHM | NDUFAF1 | RCN2 | TOMM40L |
| ATP2A1 | DIO2 | IKBKB | NDUFAF2 | RDH11 | TOMM5 |
| ATP2A2 | DISC1 | IKBKE | NDUFAF3 | RDH13 | TOMM6 |
| ATP2B2 | DLAT | IL10 | NDUFAF4 | RDH14 | TOMM7 |
| ATP5F1A | DLD | IL18 | NDUFAF5 | RECQL4 | TOMM70 |
| ATP5F1B | DLG4 | IL1B | NDUFAF6 | REEP1 | TOP1 |
| ATP5F1C | DLST | IL2 | NDUFAF7 | RELA | TOP1MT |
| ATP5F1D | DMAC1 | IL3 | NDUFAF8 | REN | TOP2A |
| ATP5F1E | DMAC2 | IL6 | NDUFB1 | RETN | TOP2B |
| ATP5F1EP2 | DMAC2L | ILF3 | NDUFB10 | REXO2 | TOP3A |
| ATP5IF1 | DMD | ILVBL | NDUFB11 | RFK | TP53 |
| ATP5MC1 | DMGDH | IMMP1L | NDUFB2 | RGN | TP53AIP1 |
| ATP5MC2 | DMPK | IMMP2L | NDUFB3 | RGS2 | TP53BP1 |
| ATP5MC3 | DNA2 | IMMT | NDUFB4 | RHBDD1 | TP73 |
| ATP5ME | DNAI4 | IMMTP1 | NDUFB5 | RHO | TPI1 |
| ATP5MF | DNAJA1 | INF2 | NDUFB6 | RHOA | TPO |
| ATP5MG | DNAJA3 | INS | NDUFB7 | RHOD | TPP1 |
| ATP5MGL | DNAJB1 | INSR | NDUFB8 | RHOT1 | TRABD |
| ATP5MJ | DNAJC11 | IQCE | NDUFB9 | RHOT2 | TRADD |
| ATP5MK | DNAJC15 | IQCN | NDUFC1 | RICTOR | TRAF2 |
| ATP5PB | DNAJC19 | IREB2 | NDUFC2 | RIDA | TRAF3 |
| ATP5PD | DNAJC27 | IRF3 | NDUFC2-KCTD14 | RIGI | TRAF6 |
| ATP5PF | DNAJC28 | IRS1 | NDUFS1 | RILP | TRAK1 |
| ATP5PO | DNAJC30 | ISCA1 | NDUFS2 | RIMKLB | TRAK2 |
| ATP6V1A | DNAJC4 | ISCA2 | NDUFS3 | RIMS3 | TRAP1 |
| ATP6V1E1 | DNAJC5 | ISCU | NDUFS4 | RIPK1 | TRIAP1 |
| ATP7A | DNASE1 | ISL1 | NDUFS5 | RIPK3 | TRIM31 |
| ATP7B | DNLZ | ISOC2 | NDUFS6 | RIPOR2 | TRIM39 |
| ATPAF1 | DNM1 | ITGAE | NDUFS7 | RMDN1 | TRIM42 |
| ATPAF2 | DNM1L | ITGAM | NDUFS8 | RMDN2 | TRIM54 |
| ATPSCKMT | DNM1P50 | ITGB1 | NDUFV1 | RMDN3 | TRIM63 |
| ATXN1 | DNM2 | ITPR1 | NDUFV2 | RMND1 | TRIT1 |
| ATXN2 | DNM3 | ITPR2 | NDUFV3 | RN7SL263P | TRMT1 |
| ATXN3 | DNMT1 | ITPR3 | NECTIN2 | RNA18SN5 | TRMT10C |
| AUH | DNTT | IVD | NEFH | RNASE1 | TRMT11 |
| AURKAIP1 | DOCK8 | JAK2 | NEFL | RNASEH1 | TRMT2B |
| AVP | DOK4 | JTB | NELFE | RNASEL | TRMT5 |
| AZIN2 | DOK6 | JUN | NEU4 | RNF144B | TRMT61B |
| B3GNT2 | DPYSL2 | KANK2 | NFATC1 | RNF185 | TRMU |
| B3GNTL1 | DPYSL4 | KARS1 | NFE2 | RNF5 | TRNT1 |
| BACE1 | DRG2 | KCNA3 | NFE2L1 | RNR2 | TRPC4AP |
| BAD | DSP | KCNA5 | NFE2L2 | ROCK1 | TRPM2 |
| BAG1 | DTNB | KCNJ11 | NFKB1 | ROMO1 | TRPV1 |
| BAG2 | DTYMK | KCNJ8 | NFKBIA | RPE65 | TRS-AGA2-3 |
| BAG5 | DUOX1 | KCTD14 | NFS1 | RPIA | TRUB2 |
| BAIAP3 | DUOX2 | KEAP1 | NFU1 | RPL10 | TSC2 |
| BAK1 | DUS2 | KIAA0930 | NGB | RPL10A | TSFM |
| BAX | DUSP1 | KIAA1191 | NGFR | RPL15 | TSHZ3 |
| BBC3 | DUSP19 | KIAA2013 | NGRN | RPL18A | TSPO |
| BBOX1 | DUSP21 | KIF11 | NIF3L1 | RPL3 | TSPOAP1 |
| BCAP31 | DUSP26 | KIF1B | NIPSNAP1 | RPL30 | TST |
| BCAT1 | DUT | KIF28P | NIPSNAP2 | RPL34 | TSTD1 |
| BCAT2 | DYNLL1 | KIF5B | NIPSNAP3A | RPL35A | TSTD3 |
| BCKDHA | DYNLL2 | KIFBP | NIPSNAP3B | RPL36AL | TTC19 |
| BCKDHB | E2F1 | KIT | NIT1 | RPL4 | TTC3 |
| BCKDK | EARS2 | KLC3 | NIT2 | RPL5 | TTC32 |
| BCL2 | EBP | KLHDC7B | NLN | RPL6 | TTC7B |
| BCL2A1 | ECH1 | KLHDC9 | NLRC4 | RPL8 | TTN |
| BCL2L1 | ECHDC1 | KLHL29 | NLRP3 | RPLP0 | TUBA1A |
| BCL2L10 | ECHDC2 | KLK6 | NLRP5 | RPLP2 | TUBB |
| BCL2L11 | ECHDC3 | KMO | NLRX1 | RPN2 | TUBB3 |
| BCL2L13 | ECHS1 | KNG1 | NME1 | RPP14 | TUBB8P5 |
| BCL2L2 | ECI1 | KRAS | NME1-NME2 | RPS10 | TUFM |
| BCLAF3 | ECI2 | KRT18 | NME2 | RPS12 | TUNAR |
| BCO2 | ECSIT | KRT5 | NME3 | RPS14 | TUSC2 |
| BCR | EDN1 | KRT8 | NME4 | RPS15A | TUSC3 |
| BCS1L | EEF1A1 | KYAT1 | NME6 | RPS18 | TUT1 |
| BDH1 | EEF1G | KYAT3 | NMNAT1 | RPS19 | TWNK |
| BDH2 | EEF2 | KYNU | NMNAT3 | RPS2 | TXN |
| BDNF | EEFSEC | L2HGDH | NMT1 | RPS3 | TXN2 |
| BECN1 | EFHD1 | LACTB | NNT | RPS4X | TXNDC12 |
| BFSP1 | EGF | LACTB2 | NOA1 | RPS6 | TXNIP |
| BHLHA15 | EGFR | LAMC1 | NOL3 | RPS6KA1 | TXNRD1 |
| BID | EGLN1 | LAMP1 | NOL6 | RPS6KA6 | TXNRD2 |
| BIK | EGR1 | LAMP2 | NOL7 | RPS6KB1 | TYMP |
| BIRC2 | EHHADH | LAP3 | NOP14 | RPS7 | TYMS |
| BIRC3 | EIF2AK2 | LARS1 | NOP9 | RPS8 | TYSND1 |
| BIRC5 | EIF2S1 | LARS2 | NOS1 | RPS9 | UACA |
| BIRC7 | EIF2S2 | LCK | NOS2 | RPSA | UBA1 |
| BLID | EIF2S3 | LDHA | NOS3 | RPTOR | UBB |
| BLOC1S1 | EIF4EBP1 | LDHAL6B | NOTCH3 | RPUSD2 | UBE2H |
| BLOC1S2 | EIF4EBP2 | LDHB | NOX1 | RPUSD3 | UBE2N |
| BLTP2 | ELAC2 | LDHD | NOX4 | RPUSD4 | UBE3B |
| BLTP3A | ELK1 | LDLR | NPC1 | RRM2B | UBIAD1 |
| BLTP3B | ELK3 | LEP | NPM1 | RRP15 | UBL4B |
| BMF | ELN | LEPR | NPPA | RSAD1 | UCHL1 |
| BMP7 | EMC2 | LETM1 | NPTX1 | RSAD2 | UCN |
| BNIP1 | EMC8 | LETM2 | NPTX2 | RSPH3 | UCP1 |
| BNIP3 | ENDOG | LETMD1 | NPY | RSRP1 | UCP2 |
| BNIP3L | ENO1 | LGALS3 | NQO1 | RTKN | UCP3 |
| BOK | ENO2 | LIAS | NR1H3 | RTL10 | UGCG |
| BOLA1 | ENOSF1 | LIG1 | NR3C1 | RTN4 | UGT1A1 |
| BOLA3 | ENTPD1 | LIG3 | NR4A1 | RTN4IP1 | ULK1 |
| BPHL | ENTREP2 | LIN28B | NR4A3 | RUNDC3B | UNG |
| BPNT1 | EPAS1 | LIPE | NR5A1 | RUNX1T1 | UOX |
| BRAF | EPHA4 | LIPF | NRDC | RXRA | UQCC1 |
| BRD8 | EPHX1 | LIPJ | NRF1 | RYR1 | UQCC2 |
| BRI3BP | EPHX2 | LIPT1 | NRIP1 | RYR2 | UQCC3 |
| BRINP3 | EPO | LIPT2 | NSUN3 | S100G | UQCC4 |
| BSG | EPRS1 | LMAN1 | NSUN4 | SACS | UQCC5 |
| BTBD16 | ERAL1 | LMNA | NT5C | SAMM50 | UQCR10 |
| BTD | ERBB2 | LMNB1 | NT5C3A | SARDH | UQCR11 |
| C14orf119 | ERBB4 | LONP1 | NT5DC2 | SARM1 | UQCRB |
| C15orf40 | ERCC6L2 | LONP2 | NT5DC3 | SARS1 | UQCRC1 |
| C15orf48 | ERGIC3 | LPCAT1 | NT5M | SARS2 | UQCRC2 |
| C15orf61 | ERI3 | LPCAT2 | NTHL1 | SAT1 | UQCRFS1 |
| C15orf62 | ERN1 | LPCAT3 | NUBPL | SCCPDH | UQCRH |
| C19orf12 | ERN2 | LPCAT4 | NUCB2 | SCD | UQCRQ |
| C1orf53 | ERO1A | LPIN1 | NUDT1 | SCO1 | URI1 |
| C1QBP | ESR1 | LPL | NUDT13 | SCO2 | UROS |
| C2orf69 | ESR2 | LRP5 | NUDT19 | SCP2 | USP30 |
| C3orf20 | ESRRA | LRPPRC | NUDT2 | SDHA | USP34 |
| C3orf33 | ESRRG | LRRC10 | NUDT5 | SDHAF1 | USP42 |
| C5orf63 | ETFA | LRRC24 | NUDT6 | SDHAF2 | USP48 |
| C6orf136 | ETFB | LRRC59 | NUDT8 | SDHAF3 | USP54 |
| C8orf82 | ETFBKMT | LRRC63 | NUDT9 | SDHAF4 | UTP23 |
| CA2 | ETFDH | LRRK1 | NUP210 | SDHB | UXS1 |
| CA5A | ETFRF1 | LRRK2 | NVL | SDHC | UXT |
| CA5B | ETHE1 | LSS | NXNL1 | SDHD | VAMP1 |
| CA5BP1 | ETNPPL | LYN | OAS1 | SDR39U1 | VAMP8 |
| CABIN1 | EVI5L | LYPLA1 | OAS2 | SDS | VAPB |
| CABS1 | EXD2 | LYPLAL1 | OAT | SDSL | VARS1 |
| CALB1 | EXOG | LYRM1 | OBSCN | SECISBP2 | VARS2 |
| CALB2 | EYA2 | LYRM2 | OCA2 | SELENOO | VASN |
| CALR | FAAH | LYRM4 | OCIAD1 | SELP | VAT1 |
| CAMK2A | FABP1 | LYRM7 | OCIAD2 | SEPTIN4 | VCP |
| CAMK4 | FABP3 | LYRM9 | OGA | SERAC1 | VDAC1 |
| CAMKK2 | FABP4 | LYSMD2 | OGDH | SERHL2 | VDAC2 |
| CANX | FABP5 | M1AP | OGDHL | SESN2 | VDAC3 |
| CAPN1 | FABP7 | MACROD1 | OGG1 | SETD4 | VDR |
| CAPN10 | FADD | MAIP1 | OGT | SETD9 | VEGFA |
| CAPN2 | FADS1 | MALSU1 | OLFM4 | SFN | VHL |
| CAPRIN2 | FAHD1 | MAN2A1 | OMA1 | SFXN1 | VHLL |
| CARD18 | FAHD2A | MAOA | OPA1 | SFXN2 | VIM |
| CARD19 | FAM107B | MAOB | OPA3 | SFXN3 | VPS13A |
| CARS2 | FAM110B | MAP1A | OPRD1 | SFXN4 | VPS13B |
| CASD1 | FAM136A | MAP1B | OPTN | SFXN5 | VPS25 |
| CASP1 | FAM162A | MAP1LC3A | ORAI1 | SGK1 | VRK2 |
| CASP10 | FAM171B | MAP1LC3B | OSBPL1A | SH3BP5 | VSX2 |
| CASP12 | FAM185A | MAP1LC3B2 | OSGEPL1 | SH3GLB1 | |
| CASP2 | FAM200C | MAP1LC3C | OTC | SHARPIN | WARS2 |
| CASP3 | FAM210A | MAP1S | OVAAL | SHC1 | WDR31 |
| CASP4 | FAM210B | MAP2 | OXA1L | SHMT1 | WDR45 |
| CASP6 | FAM222A | MAP2K1 | OXCT1 | SHMT2 | WDR45B |
| CASP7 | FAM32A | MAP2K2 | OXCT2 | SHOX2 | WDR81 |
| CASP8 | FAM72A | MAP2K3 | OXLD1 | SIAH3 | WFS1 |
| CASP8AP2 | FANCG | MAP2K4 | OXNAD1 | SIGMAR1 | WIPI1 |
| CASP9 | FARS2 | MAP3K1 | OXR1 | SIRT1 | WIPI2 |
| CASQ1 | FAS | MAP3K5 | OXSM | SIRT2 | WSCD1 |
| CAST | FASLG | MAPK1 | P2RX7 | SIRT3 | WSCD2 |
| CAT | FASN | MAPK10 | P2RY1 | SIRT4 | WWOX |
| CAV1 | FASTK | MAPK11 | P2RY12 | SIRT5 | XAF1 |
| CAV2 | FASTKD1 | MAPK12 | P4HA1 | SIRT6 | XBP1 |
| CAV3 | FASTKD2 | MAPK14 | P4HB | SIRT7 | XDH |
| CAVIN1 | FASTKD3 | MAPK3 | PABPC5 | SIVA1 | XIAP |
| CBL | FASTKD5 | MAPK8 | PACRG | SLC11A2 | XPNPEP3 |
| CBR3 | FBXL12 | MAPK8IP1 | PACS2 | SLC16A1 | XRCC3 |
| CBR4 | FBXL4 | MAPK9 | PACSIN2 | SLC16A11 | XRCC6 |
| CBS | FBXO32 | MAPKAP1 | PAICS | SLC16A3 | YAP1 |
| CCAR2 | FBXO7 | MAPT | PAK5 | SLC16A4 | YARS1 |
| CCDC127 | FBXW7 | MARCHF1 | PALLD | SLC16A7 | YARS2 |
| CCDC136 | FDPS | MARCHF2 | PAM16 | SLC1A2 | YBEY |
| CCDC191 | FDX1 | MARCHF5 | PANDAR | SLC22A4 | YIF1B |
| CCDC51 | FDX2 | MARK2 | PANK2 | SLC22A5 | YJEFN3 |
| CCDC60 | FDXR | MARS1 | PAPSS1 | SLC24A4 | YKT6 |
| CCDC90B | FECH | MARS2 | PAPSS2 | SLC25A1 | YME1L1 |
| CCK | FEN1 | MAT2B | PAQR9 | SLC25A10 | YRDC |
| CCL2 | FEZ1 | MAVS | PARG | SLC25A11 | YWHAB |
| CCNA1 | FGB | MB | PARK7 | SLC25A12 | YWHAE |
| CCNA2 | FGF2 | MBP | PARL | SLC25A13 | YWHAG |
| CCNB1 | FGF21 | MC1R | PARP1 | SLC25A14 | YWHAH |
| CCNB2 | FGFR1OP2 | MC4R | PARP4 | SLC25A15 | YWHAQ |
| CCNB3 | FGR | MCAT | PARS2 | SLC25A16 | YWHAZ |
| CCND1 | FH | MCCC1 | PARVA | SLC25A17 | YY1 |
| CCND2 | FHIT | MCCC2 | PAWR | SLC25A18 | ZDHHC8 |
| CCND3 | FIBP | MCCD1 | PC | SLC25A19 | ZFHX3 |
| CCNE1 | FIS1 | MCEE | PCBD2 | SLC25A2 | ZFY |
| CCNE2 | FITM2 | MCL1 | PCCA | SLC25A20 | ZFYVE1 |
| CCT7 | FKBP10 | MCRIP2 | PCCB | SLC25A21 | ZNF469 |
| CD2 | FKBP11 | MCU | PCK1 | SLC25A22 | ZNG1B |
| CD24 | FKBP4 | MCUB | PCK2 | SLC25A23 | ZZEF1 |

# Table S2 Mitochondrial-related protein truncating variants that were positively associated with liver dysfunction

| Chromosome | Position | Ref | Alt | Beta | SE | P value | Gene | rsid | Trait |
| --- | --- | --- | --- | --- | --- | --- | --- | --- | --- |
| 1 | 1490604 | C | A | 11.3447 | 2.20795 | 2.78E-07 | ATAD3B | novel | TBIL |
| 1 | 3396593 | G | T | 260.232 | 42.0393 | 6.01E-10 | PRDM16 | novel | GGT |
| 1 | 3732906 | C | T | 22.1726 | 4.4159 | 5.14E-07 | TP73 | novel | TBIL |
| 1 | 6358844 | A | G | 331.187 | 42.0308 | 3.29E-15 | ACOT7 | novel | GGT |
| 1 | 6385492 | A | G | 41.9425 | 7.38007 | 1.32E-08 | ACOT7 | novel | AST |
| 1 | 6625229 | C | A | 298.292 | 42.0314 | 1.28E-12 | THAP3 | rs547107923 | GGT |
| 1 | 7962872 | T | A | 24.2599 | 4.41579 | 3.93E-08 | PARK7 | novel | TBIL |
| 1 | 10297248 | T | G | 82.4249 | 14.1445 | 5.64E-09 | KIF1B | novel | ALT |
| 1 | 10297248 | T | G | 633.413 | 42.0224 | 2.51E-51 | KIF1B | novel | GGT |
| 1 | 11112897 | G | A | 53.7529 | 10.437 | 2.60E-07 | MTOR | novel | AST |
| 1 | 15524068 | C | A | 76.9098 | 14.1442 | 5.41E-08 | CASP9 | novel | ALT |
| 1 | 15524068 | C | A | 90.8141 | 10.4353 | 3.25E-18 | CASP9 | novel | AST |
| 1 | 16992019 | G | A | 128.441 | 14.1437 | 1.08E-19 | ATP13A2 | novel | ALT |
| 1 | 21706908 | C | T | 110.608 | 10.4357 | 3.04E-26 | USP48 | novel | AST |
| 1 | 21706908 | C | T | 370.492 | 42.0292 | 1.20E-18 | USP48 | novel | GGT |
| 1 | 23816732 | C | CT | 25.9605 | 4.41642 | 4.15E-09 | HMGCL | novel | TBIL |
| 1 | 25246702 | A | ACCGGC | 87.6836 | 14.1445 | 5.68E-10 | RSRP1 | novel | ALT |
| 1 | 27102681 | C | T | 30.9543 | 4.41578 | 2.39E-12 | SLC9A1 | novel | TBIL |
| 1 | 28273412 | C | T | 76.5916 | 14.1446 | 6.14E-08 | SESN2 | novel | ALT |
| 1 | 33011447 | C | T | 39.5788 | 7.48303 | 1.23E-07 | AK2 | novel | AP |
| 1 | 33082307 | C | T | 1026.57 | 42.0039 | 7.95E-132 | AZIN2 | novel | GGT |
| 1 | 36456234 | G | A | 38.0088 | 5.21833 | 3.25E-13 | MRPS15 | rs375296349 | AST |
| 1 | 36456234 | G | A | 201.791 | 21.0146 | 7.85E-22 | MRPS15 | rs375296349 | GGT |
| 1 | 39883368 | G | A | 173.953 | 29.7213 | 4.84E-09 | TRIT1 | novel | GGT |
| 1 | 45508326 | C | CA | 154.583 | 24.2672 | 1.89E-10 | MMACHC | novel | GGT |
| 1 | 45514539 | T | TGAAC | 194.964 | 25.9207 | 5.42E-14 | PRDX1 | novel | AP |
| 1 | 45514539 | T | TGAAC | 670.22 | 42.0209 | 2.98E-57 | PRDX1 | novel | GGT |
| 1 | 46310279 | A | AG | 32.293 | 6.3286 | 3.35E-07 | UQCRH | novel | ALT |
| 1 | 46643277 | G | A | 293.95 | 42.0343 | 2.69E-12 | ATPAF1 | novel | GGT |
| 1 | 66905181 | C | G | 67.6837 | 11.2363 | 1.71E-09 | DNAI4 | novel | GGT |
| 1 | 74719249 | C | CG | 27.0818 | 5.00093 | 6.12E-08 | CRYZ | novel | ALT |
| 1 | 75398422 | C | G | 194.2 | 14.142 | 6.66E-43 | SLC44A5 | novel | ALT |
| 1 | 75398422 | C | G | 96.7784 | 10.4363 | 1.81E-20 | SLC44A5 | novel | AST |
| 1 | 75728388 | G | T | 88.0352 | 10.0011 | 1.34E-18 | ACADM | novel | ALT |
| 1 | 75728388 | G | T | 212.836 | 29.7259 | 8.08E-13 | ACADM | novel | GGT |
| 1 | 77806978 | C | T | 80.8998 | 14.1437 | 1.07E-08 | MIGA1 | novel | ALT |
| 1 | 77806978 | C | T | 352.608 | 42.0309 | 4.91E-17 | MIGA1 | novel | GGT |
| 1 | 81970535 | G | A | 35.4902 | 4.41604 | 9.26E-16 | ADGRL2 | novel | TBIL |
| 1 | 81989660 | C | CTA | 27.8111 | 4.75972 | 5.13E-09 | ADGRL2 | rs774428553 | GGT |
| 1 | 94464867 | T | A | 152.027 | 29.7308 | 3.16E-07 | ABCD3 | rs774071471 | GGT |
| 1 | 100249803 | C | CA | 107.928 | 18.7974 | 9.38E-09 | DBT | novel | GGT |
| 1 | 110389267 | C | A | 26.5826 | 4.41587 | 1.75E-09 | SLC16A4 | novel | TBIL |
| 1 | 110674690 | G | T | 290.885 | 42.0537 | 4.62E-12 | KCNA3 | rs750044802 | GGT |
| 1 | 111459568 | C | T | 149.176 | 29.7213 | 5.19E-07 | ATP5PB | rs749584142 | GGT |
| 1 | 119045608 | G | A | 48.1742 | 6.02555 | 1.30E-15 | WARS2 | rs1010276895 | AST |
| 1 | 119045608 | G | A | 354.339 | 24.2621 | 2.70E-48 | WARS2 | rs1010276895 | GGT |
| 1 | 119514066 | C | A | 23.3167 | 4.4159 | 1.29E-07 | HSD3B1 | novel | TBIL |
| 1 | 119764176 | C | T | 67.3647 | 10.0016 | 1.64E-11 | HMGCS2 | novel | ALT |
| 1 | 119764176 | C | T | 42.1904 | 7.38006 | 1.09E-08 | HMGCS2 | novel | AST |
| 1 | 150499215 | G | A | 56.9441 | 4.41517 | 4.73E-38 | TARS2 | novel | TBIL |
| 1 | 150817203 | C | T | 26.0263 | 4.41586 | 3.78E-09 | ARNT | novel | TBIL |
| 1 | 151762375 | C | T | 133.901 | 14.1485 | 2.98E-21 | MRPL9 | novel | ALT |
| 1 | 151762375 | C | T | 30.7256 | 4.41735 | 3.51E-12 | MRPL9 | novel | TBIL |
| 1 | 151776199 | G | A | 23.0734 | 4.41589 | 1.74E-07 | TDRKH | rs749386396 | TBIL |
| 1 | 154273806 | G | T | 62.4287 | 8.19714 | 2.62E-14 | HAX1 | novel | AP |
| 1 | 154273806 | G | T | 35.3041 | 3.30023 | 1.05E-26 | HAX1 | novel | AST |
| 1 | 155187487 | C | T | 21.911 | 4.41596 | 6.99E-07 | MUC1 | novel | TBIL |
| 1 | 155192276 | T | C | 0.184236 | 0.0308631 | 2.38E-09 | MUC1 | rs4072037 | ALT |
| 1 | 155192276 | T | C | 0.13377 | 0.0228087 | 4.50E-09 | MUC1 | rs4072037 | AST |
| 1 | 155192276 | T | C | 0.872116 | 0.0917149 | 1.93E-21 | MUC1 | rs4072037 | GGT |
| 1 | 155610423 | A | T | 137.335 | 25.9308 | 1.18E-07 | MSTO1 | novel | AP |
| 1 | 155612188 | C | T | 30.2902 | 4.41583 | 6.92E-12 | MSTO1 | novel | TBIL |
| 1 | 156126225 | T | C | 85.9861 | 14.1444 | 1.21E-09 | LMNA | novel | ALT |
| 1 | 159890479 | C | A | 19.2063 | 3.12247 | 7.71E-10 | CFAP45 | novel | TBIL |
| 1 | 160283001 | C | A | 127.848 | 25.9216 | 8.14E-07 | PEX19 | novel | AP |
| 1 | 161119389 | G | A | 106.961 | 10.0007 | 1.08E-26 | NIT1 | novel | ALT |
| 1 | 161119389 | G | A | 93.8631 | 7.37886 | 4.62E-37 | NIT1 | novel | AST |
| 1 | 161167378 | A | T | 13.5246 | 2.6093 | 2.18E-07 | PPOX | novel | AST |
| 1 | 161169037 | G | A | 114.087 | 14.1456 | 7.33E-16 | PPOX | rs952064540 | ALT |
| 1 | 161169037 | G | A | 167.285 | 10.4353 | 8.11E-58 | PPOX | rs952064540 | AST |
| 1 | 161784097 | G | A | 46.211 | 6.02605 | 1.74E-14 | ATF6 | novel | AST |
| 1 | 165667417 | T | TC | 218.686 | 42.0322 | 1.96E-07 | ALDH9A1 | novel | GGT |
| 1 | 167883547 | G | A | 104.47 | 17.1601 | 1.14E-09 | ADCY10 | rs756835253 | GGT |
| 1 | 169613014 | G | T | 36.0245 | 4.41674 | 3.46E-16 | SELP | novel | TBIL |
| 1 | 169617328 | G | A | 30.1341 | 5.72032 | 1.38E-07 | SELP | novel | GGT |
| 1 | 171652481 | C | T | 26.7456 | 5.21964 | 2.99E-07 | MYOC | novel | AST |
| 1 | 173828401 | T | A | 250.349 | 18.326 | 1.78E-42 | DARS2 | novel | AP |
| 1 | 173828401 | T | A | 129.087 | 7.37764 | 1.60E-68 | DARS2 | novel | AST |
| 1 | 173837006 | C | T | 151.87 | 18.3285 | 1.17E-16 | DARS2 | novel | AP |
| 1 | 183110655 | G | A | 89.5841 | 14.1444 | 2.40E-10 | LAMC1 | novel | ALT |
| 1 | 183110655 | G | A | 66.8248 | 10.4368 | 1.53E-10 | LAMC1 | novel | AST |
| 1 | 193105562 | A | G | 284.75 | 42.0322 | 1.25E-11 | GLRX2 | novel | GGT |
| 1 | 220018143 | G | A | 36.8705 | 7.3802 | 5.86E-07 | EPRS1 | novel | AST |
| 1 | 220102235 | T | G | 25.615 | 4.41585 | 6.61E-09 | IARS2 | novel | TBIL |
| 1 | 220147576 | A | ATTAC | 91.0009 | 18.3295 | 6.88E-07 | IARS2 | novel | AP |
| 1 | 225844520 | C | CT | 101.655 | 14.1456 | 6.67E-13 | EPHX1 | novel | ALT |
| 1 | 226152333 | A | T | 101.464 | 14.1442 | 7.32E-13 | ACBD3 | novel | ALT |
| 1 | 226152333 | A | T | 234.145 | 42.0322 | 2.54E-08 | ACBD3 | novel | GGT |
| 1 | 226986592 | T | TC | 269.58 | 42.0314 | 1.42E-10 | COQ8A | novel | GGT |
| 1 | 228175355 | C | T | 39.3812 | 7.38018 | 9.50E-08 | IBA57 | novel | AST |
| 1 | 228216621 | C | T | 180.352 | 18.329 | 7.64E-23 | OBSCN | rs775886471 | AP |
| 1 | 228246642 | G | A | 22.4545 | 4.41593 | 3.68E-07 | OBSCN | novel | TBIL |
| 1 | 228317925 | C | G | 42.0091 | 5.00067 | 4.45E-17 | OBSCN | novel | ALT |
| 1 | 228332858 | A | G | 13.1496 | 1.67137 | 3.62E-15 | OBSCN | novel | AST |
| 1 | 228372022 | C | T | 25.5358 | 4.41585 | 7.35E-09 | OBSCN | novel | TBIL |
| 1 | 231265366 | T | G | 56.8741 | 10.4377 | 5.07E-08 | GNPAT | novel | AST |
| 1 | 231265366 | T | G | 271.263 | 42.035 | 1.10E-10 | GNPAT | novel | GGT |
| 1 | 231694187 | G | A | 91.3316 | 14.1444 | 1.07E-10 | DISC1 | novel | ALT |
| 1 | 231694187 | G | A | 478.756 | 42.0271 | 4.65E-30 | DISC1 | novel | GGT |
| 1 | 236586291 | G | T | 217.411 | 42.0323 | 2.31E-07 | HEATR1 | novel | GGT |
| 1 | 236852638 | G | A | 84.7479 | 17.1603 | 7.87E-07 | MTR | rs778242201 | GGT |
| 1 | 237454495 | C | T | 113.721 | 15.8864 | 8.18E-13 | RYR2 | novel | GGT |
| 1 | 237819193 | G | A | 78.1436 | 10.4367 | 7.04E-14 | RYR2 | novel | AST |
| 1 | 241998513 | C | T | 95.1721 | 18.3295 | 2.08E-07 | MAP1LC3C | rs149312540 | AP |
| 1 | 246548561 | T | TACAC | 71.4583 | 14.0111 | 3.40E-07 | TFB2M | novel | GGT |
| 1 | 246736056 | G | A | 148.537 | 29.733 | 5.86E-07 | SCCPDH | rs756327771 | GGT |
| 2 | 1540841 | G | GTCCGT | 68.6194 | 8.16677 | 4.39E-17 | TPO | novel | ALT |
| 2 | 1540841 | G | GTCCGT | 41.1741 | 6.02622 | 8.36E-12 | TPO | novel | AST |
| 2 | 3556786 | T | C | 57.5078 | 7.07207 | 4.25E-16 | RNASEH1 | novel | ALT |
| 2 | 3556786 | T | C | 31.4913 | 5.21939 | 1.61E-09 | RNASEH1 | novel | AST |
| 2 | 6886979 | G | T | -13.6902 | 2.58466 | 1.18E-07 | RSAD2 | novel | ALB |
| 2 | 6886979 | G | T | 262.681 | 42.0424 | 4.16E-10 | RSAD2 | novel | GGT |
| 2 | 9630451 | A | G | 449.862 | 25.9133 | 1.74E-67 | YWHAQ | novel | AP |
| 2 | 9630451 | A | G | 52.3816 | 10.437 | 5.20E-07 | YWHAQ | novel | AST |
| 2 | 9630451 | A | G | 1054.65 | 42.0025 | 4.98E-139 | YWHAQ | novel | GGT |
| 2 | 11677657 | C | T | 84.3776 | 14.1445 | 2.44E-09 | LPIN1 | novel | ALT |
| 2 | 11677657 | C | T | 220.152 | 42.0322 | 1.63E-07 | LPIN1 | novel | GGT |
| 2 | 27506887 | T | A | 261.591 | 14.1402 | 2.22E-76 | GCKR | novel | ALT |
| 2 | 27506887 | T | A | 132.613 | 25.9227 | 3.13E-07 | GCKR | novel | AP |
| 2 | 27506887 | T | A | 255.586 | 10.4305 | 1.67E-132 | GCKR | novel | AST |
| 2 | 31397691 | G | A | 69.7824 | 14.1449 | 8.08E-07 | XDH | rs756268720 | ALT |
| 2 | 31397691 | G | A | 397.595 | 42.0299 | 3.10E-21 | XDH | rs756268720 | GGT |
| 2 | 32252551 | C | A | 32.202 | 6.32576 | 3.57E-07 | NLRC4 | novel | ALT |
| 2 | 37201727 | G | GT | 68.3886 | 8.16672 | 5.58E-17 | CEBPZOS | novel | ALT |
| 2 | 37201727 | G | GT | 169.821 | 24.2791 | 2.67E-12 | CEBPZOS | novel | GGT |
| 2 | 37974039 | G | T | 59.1458 | 4.66691 | 8.43E-37 | RMDN2 | novel | AST |
| 2 | 38074345 | C | A | 78.0287 | 14.1446 | 3.46E-08 | CYP1B1 | novel | ALT |
| 2 | 38074409 | A | T | 75.319 | 14.1466 | 1.01E-07 | CYP1B1 | novel | ALT |
| 2 | 38860998 | C | A | 84.0663 | 9.79704 | 9.45E-18 | DHX57 | novel | AP |
| 2 | 43925963 | T | G | 74.9597 | 14.1447 | 1.16E-07 | LRPPRC | novel | ALT |
| 2 | 43925963 | T | G | 62.0527 | 10.4371 | 2.76E-09 | LRPPRC | novel | AST |
| 2 | 43957445 | G | A | 10.2353 | 1.39646 | 2.31E-13 | LRPPRC | rs775735922 | TBIL |
| 2 | 43995946 | A | G | 15.6566 | 3.12252 | 5.33E-07 | LRPPRC | novel | TBIL |
| 2 | 45605362 | A | C | 22.666 | 4.41592 | 2.86E-07 | SRBD1 | novel | TBIL |
| 2 | 55240062 | G | A | 529.082 | 42.026 | 2.45E-36 | MTIF2 | novel | GGT |
| 2 | 55244078 | C | CA | 332.409 | 25.9173 | 1.20E-37 | MTIF2 | novel | AP |
| 2 | 58046822 | G | C | 27.9261 | 4.41594 | 2.55E-10 | VRK2 | novel | TBIL |
| 2 | 58084138 | G | A | 51.7277 | 9.1647 | 1.66E-08 | VRK2 | novel | AP |
| 2 | 58159392 | C | CTT | 69.7972 | 14.1446 | 8.04E-07 | VRK2 | novel | ALT |
| 2 | 60960392 | T | TGA | 134.98 | 10.4245 | 2.44E-38 | PUS10 | novel | AST |
| 2 | 69419581 | A | G | 132.469 | 21.0067 | 2.87E-10 | NFU1 | novel | GGT |
| 2 | 72945081 | C | G | 101.089 | 14.1442 | 8.88E-13 | SFXN5 | novel | ALT |
| 2 | 73088751 | T | C | 22.7907 | 4.41594 | 2.46E-07 | RAB11FIP5 | novel | TBIL |
| 2 | 74873908 | A | ATG | 152.061 | 25.9216 | 4.46E-09 | HK2 | novel | AP |
| 2 | 74873908 | A | ATG | 292.95 | 42.0315 | 3.18E-12 | HK2 | novel | GGT |
| 2 | 86119045 | G | A | 75.2072 | 14.1458 | 1.06E-07 | PTCD3 | novel | ALT |
| 2 | 86130647 | G | A | 62.554 | 10.0017 | 4.00E-10 | PTCD3 | novel | ALT |
| 2 | 86147834 | C | G | 317.788 | 18.3236 | 2.35E-67 | IMMT | novel | AP |
| 2 | 86173667 | G | GT | 47.1678 | 7.38085 | 1.65E-10 | IMMT | novel | AST |
| 2 | 86207295 | C | T | 155.363 | 21.0157 | 1.44E-13 | MRPL35 | rs766826821 | GGT |
| 2 | 86788562 | T | A | 24.5381 | 4.41594 | 2.75E-08 | CD8A | rs534544636 | TBIL |
| 2 | 88127997 | G | T | 152.327 | 29.7215 | 2.97E-07 | FABP1 | novel | GGT |
| 2 | 98604193 | T | A | 59.8689 | 10.4301 | 9.47E-09 | COA5 | rs957506507 | AST |
| 2 | 99195190 | G | A | 41.4005 | 6.32566 | 5.96E-11 | MRPL30 | novel | ALT |
| 2 | 99195190 | G | A | 28.5722 | 4.66766 | 9.29E-10 | MRPL30 | novel | AST |
| 2 | 105038228 | G | C | 133.499 | 14.1436 | 3.79E-21 | MRPS9 | novel | ALT |
| 2 | 105038228 | G | C | 119.709 | 10.4356 | 1.86E-30 | MRPS9 | novel | AST |
| 2 | 105038228 | G | C | 260.01 | 42.0318 | 6.18E-10 | MRPS9 | novel | GGT |
| 2 | 111123803 | C | G | 28.1883 | 4.41585 | 1.73E-10 | BCL2L11 | novel | TBIL |
| 2 | 113499759 | T | TC | 28.5598 | 3.92293 | 3.34E-13 | FOXD4L1 | novel | ALT |
| 2 | 113499759 | T | TC | 19.2637 | 2.89465 | 2.84E-11 | FOXD4L1 | novel | AST |
| 2 | 119367436 | C | G | 81.0041 | 10.4365 | 8.41E-15 | DBI | novel | AST |
| 2 | 149582126 | C | T | 18.8991 | 3.01292 | 3.55E-10 | MMADHC | novel | AST |
| 2 | 171339472 | C | T | 26.2279 | 4.41586 | 2.86E-09 | METTL8 | novel | TBIL |
| 2 | 171893234 | G | GATCC | 51.5553 | 10.4369 | 7.83E-07 | SLC25A12 | novel | AST |
| 2 | 172961150 | G | A | 292.308 | 42.0764 | 3.73E-12 | RAPGEF4 | novel | GGT |
| 2 | 173027232 | T | TA | 159.522 | 29.7219 | 8.00E-08 | RAPGEF4 | novel | GGT |
| 2 | 178553529 | C | CATTCATTT | 89.6994 | 14.1443 | 2.27E-10 | TTN | novel | ALT |
| 2 | 178563593 | G | A | 80.7977 | 8.16575 | 4.41E-23 | TTN | novel | ALT |
| 2 | 178563593 | G | A | 55.4087 | 6.02539 | 3.74E-20 | TTN | novel | AST |
| 2 | 178572193 | G | A | 50.751 | 7.0721 | 7.18E-13 | TTN | novel | ALT |
| 2 | 178572193 | G | A | 28.4879 | 5.21845 | 4.79E-08 | TTN | novel | AST |
| 2 | 178607263 | A | AT | 24.292 | 4.4159 | 3.78E-08 | TTN | novel | TBIL |
| 2 | 178621474 | C | A | 143.603 | 18.7968 | 2.18E-14 | TTN | novel | GGT |
| 2 | 178666870 | C | CT | 42.2164 | 8.24367 | 3.04E-07 | TTN | rs765879488 | GGT |
| 2 | 178688676 | C | A | 17.1378 | 3.1225 | 4.06E-08 | TTN | novel | TBIL |
| 2 | 178694506 | A | C | 16.285 | 3.01575 | 6.67E-08 | TTN | rs772559062 | ALT |
| 2 | 178718093 | G | A | 137.303 | 24.2673 | 1.53E-08 | TTN | rs760266983 | GGT |
| 2 | 178751628 | G | C | 34.0406 | 4.41575 | 1.27E-14 | TTN | novel | TBIL |
| 2 | 178792095 | G | A | 84.5674 | 14.1445 | 2.25E-09 | TTN | novel | ALT |
| 2 | 183078939 | C | T | 239.511 | 42.0321 | 1.21E-08 | DUSP19 | novel | GGT |
| 2 | 185743174 | T | TTGTC | 273.075 | 42.0316 | 8.21E-11 | FSIP2 | novel | GGT |
| 2 | 185792640 | C | T | 61.3776 | 11.6578 | 1.40E-07 | FSIP2 | rs767059672 | GGT |
| 2 | 185801926 | G | A | 16.0516 | 3.12252 | 2.74E-07 | FSIP2 | novel | TBIL |
| 2 | 185803308 | G | T | 36.6293 | 5.77443 | 2.25E-10 | FSIP2 | novel | ALT |
| 2 | 185803308 | G | T | 33.4665 | 4.2607 | 4.02E-15 | FSIP2 | novel | AST |
| 2 | 185807934 | C | CA | 57.4461 | 10.0019 | 9.28E-09 | FSIP2 | novel | ALT |
| 2 | 185807934 | C | CA | 191.968 | 29.719 | 1.05E-10 | FSIP2 | novel | GGT |
| 2 | 185815419 | A | T | 190.725 | 24.2651 | 3.85E-15 | FSIP2 | novel | GGT |
| 2 | 190057416 | T | TA | 61.9124 | 10.4368 | 2.99E-09 | MSTN | novel | AST |
| 2 | 190895250 | T | A | 382.439 | 29.7321 | 7.41E-38 | GLS | novel | GGT |
| 2 | 196306607 | G | A | 124.321 | 14.1437 | 1.50E-18 | HECW2 | rs756320580 | ALT |
| 2 | 196306607 | G | A | 95.2449 | 10.4362 | 7.11E-20 | HECW2 | rs756320580 | AST |
| 2 | 196306607 | G | A | 226.196 | 42.0321 | 7.39E-08 | HECW2 | rs756320580 | GGT |
| 2 | 197706441 | C | T | 70.7083 | 14.1446 | 5.77E-07 | MARS2 | novel | ALT |
| 2 | 197706441 | C | T | 165.527 | 10.4341 | 1.17E-56 | MARS2 | novel | AST |
| 2 | 199911441 | G | T | 71.0294 | 9.16445 | 9.17E-15 | C2orf69 | novel | AP |
| 2 | 199911441 | G | T | 8.82058 | 1.56127 | 1.61E-08 | C2orf69 | novel | TBIL |
| 2 | 199955495 | A | G | 87.2257 | 17.1595 | 3.71E-07 | MAIP1 | novel | GGT |
| 2 | 200621244 | A | T | 263.326 | 42.0315 | 3.73E-10 | AOX1 | novel | GGT |
| 2 | 200641185 | G | C | 12.3958 | 2.26517 | 4.44E-08 | AOX1 | rs766739242 | ALT |
| 2 | 200641185 | G | C | 8.25287 | 1.67148 | 7.92E-07 | AOX1 | rs766739242 | AST |
| 2 | 200861420 | G | A | 145.427 | 25.9216 | 2.02E-08 | CLK1 | novel | AP |
| 2 | 200903669 | G | A | 32.9693 | 4.41575 | 8.26E-14 | NIF3L1 | novel | TBIL |
| 2 | 201136108 | G | T | 130.616 | 25.9216 | 4.68E-07 | CFLAR | novel | AP |
| 2 | 201136108 | G | T | 726.806 | 42.0185 | 5.20E-67 | CFLAR | novel | GGT |
| 2 | 201160827 | C | T | 32.2666 | 6.02579 | 8.57E-08 | CFLAR | novel | AST |
| 2 | 201387976 | C | G | 46.5999 | 8.16662 | 1.16E-08 | TRAK2 | novel | ALT |
| 2 | 206126766 | G | A | 62.8443 | 12.6736 | 7.10E-07 | NDUFS1 | rs762369653 | GGT |
| 2 | 206767047 | G | GTC | 23.714 | 4.41592 | 7.87E-08 | FASTKD2 | novel | TBIL |
| 2 | 210192803 | C | G | 65.1727 | 10.0017 | 7.22E-11 | ACADL | novel | ALT |
| 2 | 210192803 | C | G | 111.517 | 7.37845 | 1.35E-51 | ACADL | novel | AST |
| 2 | 210192803 | C | G | 169.721 | 29.7222 | 1.13E-08 | ACADL | novel | GGT |
| 2 | 215011631 | G | A | 58.8799 | 9.16463 | 1.32E-10 | ABCA12 | rs763481375 | AP |
| 2 | 215045919 | G | A | 285.45 | 42.0314 | 1.11E-11 | ABCA12 | novel | GGT |
| 2 | 215052507 | G | T | 18.3274 | 3.0129 | 1.18E-09 | ABCA12 | novel | AST |
| 2 | 218661896 | C | T | 32.9772 | 6.32576 | 1.86E-07 | BCS1L | novel | ALT |
| 2 | 218661896 | C | T | 30.7108 | 4.66753 | 4.72E-11 | BCS1L | novel | AST |
| 2 | 218661896 | C | T | 116.159 | 18.7975 | 6.44E-10 | BCS1L | novel | GGT |
| 2 | 218812596 | C | T | 33.868 | 6.32573 | 8.61E-08 | CYP27A1 | rs72551315 | ALT |
| 2 | 219425662 | G | A | 215.502 | 42.0322 | 2.94E-07 | DES | novel | GGT |
| 2 | 223959778 | G | T | 78.1547 | 14.1446 | 3.29E-08 | MRPL44 | novel | ALT |
| 2 | 227342820 | G | A | 37.8241 | 7.48309 | 4.31E-07 | MFF | novel | AP |
| 2 | 228018255 | G | A | 22.3476 | 4.41589 | 4.18E-07 | SPHKAP | rs867742232 | TBIL |
| 2 | 229232053 | A | G | 149.739 | 25.9213 | 7.62E-09 | PID1 | rs750129687 | AP |
| 2 | 229262738 | A | T | 32.7691 | 5.21841 | 3.40E-10 | PID1 | novel | AST |
| 2 | 229262738 | A | T | 169.973 | 21.0152 | 6.08E-16 | PID1 | novel | GGT |
| 2 | 230416882 | G | A | 25.2818 | 4.41587 | 1.03E-08 | SP100 | novel | TBIL |
| 2 | 232633903 | G | A | 143.156 | 21.0166 | 9.67E-12 | EFHD1 | rs550603455 | GGT |
| 2 | 233277674 | G | A | 198.306 | 24.2665 | 3.04E-16 | ATG16L1 | novel | GGT |
| 2 | 233760634 | T | TA | 150.165 | 7.37671 | 4.49E-92 | UGT1A1 | rs748219743 | AST |
| 2 | 233760634 | T | TA | 26.4712 | 3.12238 | 2.30E-17 | UGT1A1 | rs748219743 | TBIL |
| 2 | 233760935 | C | CT | 58.8602 | 4.41521 | 1.55E-40 | UGT1A1 | novel | TBIL |
| 2 | 233761020 | C | T | 7.71108 | 1.04082 | 1.28E-13 | UGT1A1 | novel | TBIL |
| 2 | 240011663 | G | A | 6.96327 | 1.39645 | 6.15E-07 | NDUFA10 | novel | TBIL |
| 2 | 240021204 | C | G | 80.9964 | 14.1445 | 1.03E-08 | NDUFA10 | novel | ALT |
| 2 | 240021204 | C | G | 57.0808 | 10.4369 | 4.53E-08 | NDUFA10 | novel | AST |
| 2 | 240594673 | G | GTCAC | 127.894 | 21.0159 | 1.16E-09 | CAPN10 | novel | GGT |
| 2 | 241096246 | C | CA | 30.833 | 5.77464 | 9.33E-08 | MTERF4 | novel | ALT |
| 2 | 241096246 | C | CA | 91.1442 | 10.5821 | 7.14E-18 | MTERF4 | novel | AP |
| 2 | 241096246 | C | CA | 106.49 | 17.1598 | 5.45E-10 | MTERF4 | novel | GGT |
| 2 | 241743681 | C | T | 42.3991 | 7.81578 | 5.80E-08 | D2HGDH | novel | AP |
| 2 | 241767711 | A | G | 124.992 | 18.3349 | 9.30E-12 | D2HGDH | rs113782371 | AP |
| 2 | 241767711 | A | G | 408.358 | 29.7254 | 6.17E-43 | D2HGDH | rs113782371 | GGT |
| 3 | 3140608 | T | TG | 102.948 | 10.4375 | 6.04E-23 | TRNT1 | novel | AST |
| 3 | 9838148 | C | CA | 56.4713 | 10.4369 | 6.28E-08 | RPUSD3 | novel | AST |
| 3 | 11306959 | G | GCACTCCTAGATACAGCAACT | 20.7952 | 3.94483 | 1.35E-07 | ATG7 | novel | AST |
| 3 | 11306959 | G | GCACTCCTAGATACAGCAACT | 99.4441 | 15.8866 | 3.86E-10 | ATG7 | novel | GGT |
| 3 | 12433936 | A | G | 26.9495 | 3.92297 | 6.44E-12 | PPARG | novel | ALT |
| 3 | 12433936 | A | G | 25.1246 | 2.89457 | 3.97E-18 | PPARG | novel | AST |
| 3 | 13339909 | G | GA | 123.614 | 24.2674 | 3.51E-07 | NUP210 | novel | GGT |
| 3 | 13358221 | C | G | 25.2048 | 4.41589 | 1.15E-08 | NUP210 | novel | TBIL |
| 3 | 13379629 | C | CCAAGACAG | 36.3681 | 4.41568 | 1.78E-16 | NUP210 | novel | TBIL |
| 3 | 14761610 | A | AT | 78.111 | 10.4366 | 7.20E-14 | C3orf20 | novel | AST |
| 3 | 14761610 | A | AT | 816.068 | 42.0148 | 5.33E-84 | C3orf20 | novel | GGT |
| 3 | 37011857 | A | T | 25.0658 | 4.41593 | 1.38E-08 | MLH1 | rs863225383 | TBIL |
| 3 | 38126657 | C | T | 73.9857 | 14.145 | 1.69E-07 | ACAA1 | novel | ALT |
| 3 | 38129289 | C | T | 77.0223 | 10.4366 | 1.59E-13 | ACAA1 | novel | AST |
| 3 | 38506856 | T | A | 22.716 | 4.41565 | 2.68E-07 | EXOG | novel | TBIL |
| 3 | 42210215 | T | TC | 145.867 | 24.2671 | 1.85E-09 | TRAK1 | novel | GGT |
| 3 | 42220504 | G | C | 107.109 | 14.9654 | 8.25E-13 | TRAK1 | novel | AP |
| 3 | 42220504 | G | C | 226.693 | 24.2656 | 9.48E-21 | TRAK1 | novel | GGT |
| 3 | 42263416 | C | T | 19.785 | 3.92307 | 4.58E-07 | CCK | rs765774285 | ALT |
| 3 | 42263416 | C | T | 18.3002 | 2.89468 | 2.58E-10 | CCK | rs765774285 | AST |
| 3 | 47810685 | T | A | 118.285 | 14.1439 | 6.13E-17 | DHX30 | novel | ALT |
| 3 | 47810685 | T | A | 74.7862 | 10.4367 | 7.75E-13 | DHX30 | novel | AST |
| 3 | 48295139 | C | T | 5.20334 | 0.995273 | 1.71E-07 | NME6 | rs199785467 | AST |
| 3 | 48604321 | G | A | 16.0056 | 2.39442 | 2.32E-11 | UQCRC1 | rs369886828 | AST |
| 3 | 49023167 | C | T | 77.1414 | 10.0056 | 1.26E-14 | NDUFAF3 | rs527841371 | ALT |
| 3 | 49104685 | C | A | 32.0835 | 4.41575 | 3.72E-13 | QARS1 | rs546130184 | TBIL |
| 3 | 49357565 | G | A | 70.4251 | 10.8529 | 8.65E-11 | GPX1 | rs757196709 | GGT |
| 3 | 49417974 | C | T | 77.4647 | 10.0511 | 1.29E-14 | AMT | rs181134220 | ALT |
| 3 | 49417974 | C | T | 41.1731 | 7.41705 | 2.84E-08 | AMT | rs181134220 | AST |
| 3 | 49419319 | A | AC | 48.4235 | 7.37989 | 5.33E-11 | AMT | novel | AST |
| 3 | 53230544 | C | CCCAT | 28.9307 | 5.34621 | 6.26E-08 | TKT | novel | ALT |
| 3 | 53230544 | C | CCCAT | 26.4077 | 3.94476 | 2.17E-11 | TKT | novel | AST |
| 3 | 53234981 | A | AC | 71.9865 | 14.1446 | 3.60E-07 | TKT | novel | ALT |
| 3 | 53818977 | G | A | 163.579 | 29.722 | 3.72E-08 | CHDH | novel | GGT |
| 3 | 53818977 | G | A | 17.1226 | 3.12255 | 4.17E-08 | CHDH | novel | TBIL |
| 3 | 111986304 | G | T | 34.2707 | 5.52659 | 5.61E-10 | ABHD10 | rs371246123 | AP |
| 3 | 112991038 | C | T | 27.0163 | 3.53609 | 2.17E-14 | GTPBP8 | novel | ALT |
| 3 | 114046733 | C | T | 40.0312 | 4.4158 | 1.25E-19 | CCDC191 | novel | TBIL |
| 3 | 119866589 | C | T | 163.202 | 29.7241 | 4.01E-08 | GSK3B | novel | GGT |
| 3 | 121632468 | G | GGGCTCAGGCTCA | 0.359196 | 0.068134 | 1.35E-07 | HCLS1 | rs150627065 | AP |
| 3 | 140688364 | C | A | 15.6942 | 3.12252 | 5.01E-07 | TRIM42 | novel | TBIL |
| 3 | 152835866 | C | CACGGT | 79.3122 | 14.9659 | 1.16E-07 | P2RY1 | novel | AP |
| 3 | 152835866 | C | CACGGT | 164.236 | 24.2669 | 1.31E-11 | P2RY1 | novel | GGT |
| 3 | 158653319 | C | A | 86.9883 | 14.8609 | 4.82E-09 | GFM1 | novel | GGT |
| 3 | 172518466 | C | T | 143.207 | 14.1441 | 4.31E-24 | TNFSF10 | novel | ALT |
| 3 | 172518466 | C | T | 57.4305 | 10.4375 | 3.75E-08 | TNFSF10 | novel | AST |
| 3 | 172647875 | C | T | 229.051 | 42.032 | 5.06E-08 | NCEH1 | novel | GGT |
| 3 | 179598744 | C | T | 72.4496 | 10.0015 | 4.37E-13 | MRPL47 | novel | ALT |
| 3 | 183052169 | A | G | 53.6117 | 5.7742 | 1.63E-20 | MCCC1 | rs150862707 | ALT |
| 3 | 183052169 | A | G | 83.3662 | 10.5822 | 3.33E-15 | MCCC1 | rs150862707 | AP |
| 3 | 183052169 | A | G | 21.2119 | 4.26095 | 6.42E-07 | MCCC1 | rs150862707 | AST |
| 3 | 183052169 | A | G | 109.648 | 17.1596 | 1.66E-10 | MCCC1 | rs150862707 | GGT |
| 3 | 197512246 | G | T | 113.536 | 14.144 | 1.00E-15 | BDH1 | novel | ALT |
| 3 | 197512246 | G | T | 596.682 | 25.9062 | 2.61E-117 | BDH1 | novel | AP |
| 3 | 197512246 | G | T | 111.517 | 10.4359 | 1.19E-26 | BDH1 | novel | AST |
| 3 | 197512246 | G | T | 908.732 | 42.0103 | 1.04E-103 | BDH1 | novel | GGT |
| 4 | 1822313 | C | T | 149.787 | 14.1433 | 3.32E-26 | LETM1 | novel | ALT |
| 4 | 1822313 | C | T | 135.655 | 10.4353 | 1.25E-38 | LETM1 | novel | AST |
| 4 | 6295004 | G | A | 69.9239 | 14.1447 | 7.68E-07 | WFS1 | novel | ALT |
| 4 | 8381501 | G | A | 15.476 | 3.12261 | 7.20E-07 | ACOX3 | novel | TBIL |
| 4 | 24799522 | A | C | 16.0331 | 3.12251 | 2.83E-07 | SOD3 | novel | TBIL |
| 4 | 42020431 | A | AG | 23.368 | 4.41546 | 1.21E-07 | SLC30A9 | novel | TBIL |
| 4 | 46734947 | T | A | 24.1945 | 4.4159 | 4.28E-08 | COX7B2 | rs113832521 | TBIL |
| 4 | 47554880 | T | TA | 77.9995 | 14.0111 | 2.59E-08 | ATP10D | novel | GGT |
| 4 | 47557965 | C | CA | 24.2217 | 4.47305 | 6.13E-08 | ATP10D | novel | ALT |
| 4 | 56435929 | G | A | 31.3131 | 4.41576 | 1.33E-12 | PAICS | novel | TBIL |
| 4 | 73408735 | C | T | -7.95909 | 0.976589 | 3.65E-16 | ALB | rs77238412 | ALB |
| 4 | 73416354 | G | A | -7.70186 | 0.645891 | 8.95E-33 | ALB | rs779988470 | ALB |
| 4 | 73419640 | G | C | -6.46356 | 0.690536 | 8.00E-21 | ALB | rs1800580 | ALB |
| 4 | 83459739 | G | A | 62.4433 | 12.1341 | 2.66E-07 | MRPS18C | rs763890388 | GGT |
| 4 | 88092359 | C | T | 285.679 | 25.9187 | 3.02E-28 | ABCG2 | novel | AP |
| 4 | 88092359 | C | T | 208.149 | 42.0323 | 7.34E-07 | ABCG2 | novel | GGT |
| 4 | 99076291 | C | A | 203.02 | 29.7206 | 8.45E-12 | ADH5 | novel | GGT |
| 4 | 109960980 | C | T | 73.7031 | 11.6579 | 2.58E-10 | EGF | rs369702571 | GGT |
| 4 | 109993335 | T | TGGAC | 96.0391 | 14.1443 | 1.12E-11 | EGF | novel | ALT |
| 4 | 109993335 | T | TGGAC | 1032.24 | 25.8737 | 0 | EGF | novel | AP |
| 4 | 109993335 | T | TGGAC | 65.6015 | 10.4368 | 3.27E-10 | EGF | novel | AST |
| 4 | 110011282 | T | A | 25.1432 | 4.41586 | 1.24E-08 | EGF | rs991255855 | TBIL |
| 4 | 122876367 | A | AGG | 108.81 | 18.3293 | 2.92E-09 | FGF2 | novel | AP |
| 4 | 122876367 | A | AGG | 175.487 | 29.7212 | 3.54E-09 | FGF2 | novel | GGT |
| 4 | 139268746 | G | C | 154.24 | 14.1434 | 1.10E-27 | MGARP | novel | ALT |
| 4 | 139268746 | G | C | 114.235 | 10.4361 | 6.99E-28 | MGARP | novel | AST |
| 4 | 145110988 | C | A | 180.329 | 14.1424 | 3.13E-37 | ABCE1 | novel | ALT |
| 4 | 145110988 | C | A | 66.3016 | 10.4368 | 2.12E-10 | ABCE1 | novel | AST |
| 4 | 145110988 | C | A | 412.445 | 42.0289 | 9.92E-23 | ABCE1 | novel | GGT |
| 4 | 145655304 | T | G | 27.2868 | 4.41583 | 6.44E-10 | MMAA | novel | TBIL |
| 4 | 158710770 | G | A | 119.649 | 14.1462 | 2.73E-17 | PPID | novel | ALT |
| 4 | 158717190 | T | TC | 68.077 | 10.4368 | 6.91E-11 | PPID | novel | AST |
| 4 | 168165282 | G | T | 227.827 | 29.721 | 1.78E-14 | ANXA10 | novel | GGT |
| 4 | 168683105 | T | C | 233.259 | 24.2649 | 7.08E-22 | PALLD | novel | GGT |
| 5 | 233635 | C | T | 20.5247 | 3.12252 | 4.93E-11 | SDHA | rs746165168 | TBIL |
| 5 | 1293970 | C | CTG | 243.766 | 42.0317 | 6.65E-09 | TERT | novel | GGT |
| 5 | 1801333 | C | A | 75.0417 | 14.1445 | 1.12E-07 | NDUFS6 | novel | ALT |
| 5 | 33445366 | A | T | 30.658 | 4.41577 | 3.85E-12 | TARS1 | novel | TBIL |
| 5 | 33461989 | T | TA | 152.461 | 29.7214 | 2.90E-07 | TARS1 | novel | GGT |
| 5 | 34005781 | G | A | 101.405 | 10.4371 | 2.60E-22 | AMACR | rs775128044 | AST |
| 5 | 34005781 | G | A | 272.401 | 42.0357 | 9.17E-11 | AMACR | rs775128044 | GGT |
| 5 | 36208691 | C | T | 239.195 | 25.92 | 2.76E-20 | NADK2 | novel | AP |
| 5 | 36241745 | G | GCCGC | 50.0419 | 10.0018 | 5.64E-07 | NADK2 | novel | ALT |
| 5 | 37834649 | C | CA | 177.023 | 14.1425 | 6.11E-36 | GDNF | novel | ALT |
| 5 | 37834649 | C | CA | 63.9766 | 10.4369 | 8.80E-10 | GDNF | novel | AST |
| 5 | 44815035 | C | T | 224.935 | 42.0323 | 8.73E-08 | MRPS30 | novel | GGT |
| 5 | 55283202 | C | T | 91.3315 | 14.1444 | 1.07E-10 | DHX29 | novel | ALT |
| 5 | 55283202 | C | T | 478.756 | 42.0271 | 4.65E-30 | DHX29 | novel | GGT |
| 5 | 55297323 | C | A | 97.636 | 10.4383 | 8.50E-21 | DHX29 | novel | AST |
| 5 | 56911317 | A | T | 228.001 | 42.0321 | 5.82E-08 | SETD9 | novel | GGT |
| 5 | 71602572 | G | C | 128.804 | 25.9235 | 6.75E-07 | MCCC2 | rs151098534 | AP |
| 5 | 71650185 | T | A | 122.796 | 14.9653 | 2.31E-16 | MCCC2 | novel | AP |
| 5 | 72226113 | G | A | -14.7213 | 2.58389 | 1.22E-08 | MRPS27 | novel | ALB |
| 5 | 72226113 | G | A | 176.185 | 25.921 | 1.07E-11 | MRPS27 | novel | AP |
| 5 | 72226113 | G | A | 77.835 | 10.4366 | 8.81E-14 | MRPS27 | novel | AST |
| 5 | 72226113 | G | A | 341.607 | 42.0302 | 4.39E-16 | MRPS27 | novel | GGT |
| 5 | 72226113 | G | A | 46.8193 | 4.41544 | 2.89E-26 | MRPS27 | novel | TBIL |
| 5 | 72238046 | G | A | 70.684 | 14.1447 | 5.82E-07 | MRPS27 | rs563887275 | ALT |
| 5 | 79054306 | T | TG | 86.2635 | 4.41404 | 5.16E-85 | DMGDH | novel | TBIL |
| 5 | 79063619 | C | T | 91.6254 | 14.1445 | 9.32E-11 | DMGDH | novel | ALT |
| 5 | 79063619 | C | T | 75.8166 | 10.4367 | 3.75E-13 | DMGDH | novel | AST |
| 5 | 79991226 | A | AG | 121.105 | 14.1438 | 1.11E-17 | MTX3 | novel | ALT |
| 5 | 79991226 | A | AG | 165.657 | 25.9204 | 1.65E-10 | MTX3 | novel | AP |
| 5 | 79991226 | A | AG | 60.584 | 10.4368 | 6.45E-09 | MTX3 | novel | AST |
| 5 | 81263608 | C | T | 23.3888 | 4.4159 | 1.18E-07 | CKMT2 | rs778422458 | TBIL |
| 5 | 109784793 | A | T | 54.1375 | 10.4369 | 2.14E-07 | MAN2A1 | novel | AST |
| 5 | 109817385 | C | T | 30.2768 | 4.41583 | 7.07E-12 | MAN2A1 | novel | TBIL |
| 5 | 110761701 | T | G | 85.7694 | 17.1602 | 5.79E-07 | SLC25A46 | novel | GGT |
| 5 | 115840496 | C | A | 84.8998 | 10.4298 | 3.96E-16 | ATG12 | novel | AST |
| 5 | 119536421 | A | C | 294.01 | 42.0324 | 2.66E-12 | HSD17B4 | novel | GGT |
| 5 | 126549936 | A | AC | 131.399 | 14.1436 | 1.55E-20 | ALDH7A1 | novel | ALT |
| 5 | 126549936 | A | AC | 203.699 | 25.9206 | 3.89E-15 | ALDH7A1 | novel | AP |
| 5 | 126549936 | A | AC | 298.485 | 42.0311 | 1.24E-12 | ALDH7A1 | novel | GGT |
| 5 | 126559330 | A | G | 24.9807 | 4.41612 | 1.54E-08 | ALDH7A1 | novel | TBIL |
| 5 | 132331851 | G | A | 82.5115 | 14.1456 | 5.45E-09 | SLC22A4 | novel | ALT |
| 5 | 132331851 | G | A | 60.4343 | 10.4378 | 7.05E-09 | SLC22A4 | novel | AST |
| 5 | 132378475 | C | G | 74.7237 | 14.1446 | 1.27E-07 | SLC22A5 | rs1057516765 | ALT |
| 5 | 132378475 | C | G | 248.237 | 42.0326 | 3.51E-09 | SLC22A5 | rs1057516765 | GGT |
| 5 | 132393675 | G | A | 212.118 | 42.0325 | 4.50E-07 | SLC22A5 | rs386134224 | GGT |
| 5 | 132900871 | C | A | 152.421 | 29.721 | 2.92E-07 | AFF4 | novel | GGT |
| 5 | 138145246 | C | G | 86.0494 | 10.4364 | 1.65E-16 | BRD8 | novel | AST |
| 5 | 138171382 | A | G | 177.879 | 25.9211 | 6.78E-12 | BRD8 | novel | AP |
| 5 | 138329552 | C | A | 60.5777 | 10.438 | 6.50E-09 | CDC25C | novel | AST |
| 5 | 139481306 | G | T | 14.7143 | 2.54951 | 7.87E-09 | STING1 | novel | TBIL |
| 5 | 140697381 | T | TC | 225.972 | 42.032 | 7.61E-08 | HARS2 | novel | GGT |
| 5 | 146142888 | T | C | 26.0418 | 5.21856 | 6.03E-07 | LARS1 | novel | AST |
| 5 | 146172736 | A | G | 158.219 | 25.9219 | 1.04E-09 | LARS1 | novel | AP |
| 5 | 151133159 | C | T | 170.308 | 29.7212 | 1.00E-08 | ANXA6 | novel | GGT |
| 5 | 157731445 | G | T | 277.028 | 42.0331 | 4.38E-11 | THG1L | rs371761718 | GGT |
| 5 | 175492146 | C | T | 11.2879 | 2.20796 | 3.18E-07 | SFXN1 | rs762041499 | TBIL |
| 5 | 179722918 | A | AT | 432.817 | 25.8974 | 1.11E-62 | CANX | novel | AP |
| 5 | 179836453 | A | AT | 157.632 | 11.5904 | 4.07E-42 | SQSTM1 | novel | AP |
| 5 | 179879199 | T | G | 103.112 | 7.37853 | 2.28E-44 | TBC1D9B | novel | AST |
| 5 | 179879199 | T | G | 151.911 | 29.7212 | 3.20E-07 | TBC1D9B | novel | GGT |
| 5 | 179894597 | C | A | 68.0507 | 12.9608 | 1.52E-07 | TBC1D9B | rs780583574 | AP |
| 5 | 180269410 | C | G | 438.902 | 42.0306 | 1.60E-25 | MAPK9 | novel | GGT |
| 5 | 181242285 | C | T | 468.399 | 42.0272 | 7.63E-29 | RACK1 | novel | GGT |
| 6 | 3083282 | G | A | 73.1062 | 14.1451 | 2.36E-07 | RIPK1 | novel | ALT |
| 6 | 3083282 | G | A | 71.7574 | 10.437 | 6.20E-12 | RIPK1 | novel | AST |
| 6 | 7579385 | C | G | 25.4773 | 4.41585 | 7.96E-09 | DSP | rs886039178 | TBIL |
| 6 | 8420818 | A | G | 217.606 | 25.9217 | 4.68E-17 | SLC35B3 | novel | AP |
| 6 | 13620245 | C | G | 41.3402 | 7.38017 | 2.13E-08 | NOL7 | novel | AST |
| 6 | 24875698 | G | A | 59.6115 | 10.0017 | 2.52E-09 | RIPOR2 | novel | ALT |
| 6 | 24875698 | G | A | 43.347 | 7.38004 | 4.27E-09 | RIPOR2 | novel | AST |
| 6 | 30914470 | G | A | 31.4926 | 4.6675 | 1.51E-11 | VARS2 | novel | AST |
| 6 | 31781125 | T | C | 53.1229 | 9.17237 | 6.98E-09 | VARS1 | novel | GGT |
| 6 | 31785594 | G | A | 59.4239 | 10.4368 | 1.24E-08 | VARS1 | novel | AST |
| 6 | 31785594 | G | A | 369.781 | 42.0296 | 1.40E-18 | VARS1 | novel | GGT |
| 6 | 31791710 | G | A | 148.751 | 29.7214 | 5.59E-07 | VARS1 | novel | GGT |
| 6 | 31792986 | G | A | 49.4598 | 7.3799 | 2.06E-11 | VARS1 | rs138074588 | AST |
| 6 | 33573908 | C | G | 70.8189 | 14.145 | 5.54E-07 | BAK1 | novel | ALT |
| 6 | 33575860 | G | A | 443.504 | 29.7145 | 2.32E-50 | BAK1 | novel | GGT |
| 6 | 33667282 | A | T | 312.528 | 42.0308 | 1.04E-13 | ITPR3 | novel | GGT |
| 6 | 33711685 | A | G | 74.3149 | 10.4367 | 1.08E-12 | UQCC2 | novel | AST |
| 6 | 35795148 | A | G | 56.1223 | 10.5828 | 1.14E-07 | CLPS | rs898044867 | AP |
| 6 | 39906868 | C | T | 28.2738 | 5.00093 | 1.57E-08 | MOCS1 | rs780148535 | ALT |
| 6 | 39906868 | C | T | 33.3236 | 3.68984 | 1.71E-19 | MOCS1 | rs780148535 | AST |
| 6 | 41733916 | A | T | 57.5996 | 10.4372 | 3.42E-08 | TFEB | novel | AST |
| 6 | 42214279 | C | A | 22.8616 | 4.41592 | 2.25E-07 | MRPS10 | novel | TBIL |
| 6 | 43056194 | G | C | 36.8121 | 5.21835 | 1.74E-12 | MRPL2 | novel | AST |
| 6 | 46658924 | T | G | 25.5822 | 4.41522 | 6.87E-09 | SLC25A27 | novel | TBIL |
| 6 | 47879269 | C | A | 44.2687 | 6.32562 | 2.59E-12 | PTCHD4 | novel | ALT |
| 6 | 47879269 | C | A | 142.524 | 18.7971 | 3.40E-14 | PTCHD4 | novel | GGT |
| 6 | 49457990 | G | A | 125.066 | 24.2879 | 2.62E-07 | MMUT | rs780068818 | GGT |
| 6 | 73500573 | G | T | 30.896 | 4.41579 | 2.62E-12 | MTO1 | novel | TBIL |
| 6 | 75240275 | A | C | 93.1561 | 14.1475 | 4.56E-11 | COX7A2 | novel | ALT |
| 6 | 75240275 | A | C | 231.361 | 42.0498 | 3.76E-08 | COX7A2 | novel | GGT |
| 6 | 83223876 | G | A | 40.0832 | 7.38007 | 5.60E-08 | ME1 | novel | AST |
| 6 | 88145267 | G | T | 22.1665 | 4.41594 | 5.18E-07 | CNR1 | novel | TBIL |
| 6 | 121292117 | T | TA | 34.3277 | 4.41589 | 7.64E-15 | TBC1D32 | novel | TBIL |
| 6 | 136233252 | G | A | 176.989 | 10.4376 | 1.80E-64 | MTFR2 | novel | AST |
| 6 | 136592525 | C | CA | 56.085 | 10.437 | 7.72E-08 | MAP3K5 | novel | AST |
| 6 | 143433935 | C | T | 108.587 | 21.0162 | 2.38E-07 | ADAT2 | rs750252135 | GGT |
| 6 | 146715381 | G | A | 39.9122 | 7.38039 | 6.38E-08 | ADGB | novel | AST |
| 6 | 146763899 | A | C | 63.598 | 10.4368 | 1.10E-09 | ADGB | novel | AST |
| 6 | 146763899 | A | C | 227.433 | 42.0312 | 6.27E-08 | ADGB | novel | GGT |
| 6 | 151092465 | A | G | 134.434 | 25.9217 | 2.15E-07 | MTHFD1L | novel | AP |
| 6 | 159797291 | C | T | 135.189 | 8.19489 | 4.05E-61 | MRPL18 | rs375117381 | AP |
| 6 | 159797291 | C | T | 73.7199 | 14.0111 | 1.43E-07 | MRPL18 | rs375117381 | GGT |
| 6 | 159797499 | G | A | 70.8705 | 14.1447 | 5.43E-07 | MRPL18 | novel | ALT |
| 6 | 159797499 | G | A | 583.763 | 25.907 | 2.29E-112 | MRPL18 | novel | AP |
| 6 | 159797499 | G | A | 113.838 | 10.4358 | 1.06E-27 | MRPL18 | novel | AST |
| 6 | 159797499 | G | A | 382.554 | 42.0296 | 8.90E-20 | MRPL18 | novel | GGT |
| 6 | 159797499 | G | A | 106.707 | 4.41298 | 4.41E-129 | MRPL18 | novel | TBIL |
| 6 | 161569417 | C | T | 142.021 | 10.4351 | 3.57E-42 | PRKN | novel | AST |
| 6 | 161569417 | C | T | 331.822 | 42.0309 | 2.92E-15 | PRKN | novel | GGT |
| 7 | 5374433 | T | TCC | 23.9905 | 4.41588 | 5.55E-08 | TNRC18 | novel | TBIL |
| 7 | 6111123 | A | G | 22.5038 | 4.41589 | 3.47E-07 | USP42 | novel | TBIL |
| 7 | 17327849 | G | T | 15.2376 | 2.20742 | 5.10E-12 | AHR | novel | TBIL |
| 7 | 23307927 | T | TG | 22.5321 | 4.26099 | 1.24E-07 | MALSU1 | rs757014635 | AST |
| 7 | 24289548 | A | T | 12.7268 | 2.54939 | 5.98E-07 | NPY | novel | TBIL |
| 7 | 27538339 | A | AC | 317.051 | 42.0408 | 4.66E-14 | HIBADH | novel | GGT |
| 7 | 30633859 | G | A | 97.3506 | 12.6778 | 1.61E-14 | GARS1 | rs905239894 | GGT |
| 7 | 48245511 | G | A | 182.636 | 14.1426 | 3.82E-38 | ABCA13 | novel | ALT |
| 7 | 48245511 | G | A | 84.4134 | 10.4367 | 6.07E-16 | ABCA13 | novel | AST |
| 7 | 48248250 | G | C | 62.2775 | 8.19702 | 3.02E-14 | ABCA13 | novel | AP |
| 7 | 48248250 | G | C | 66.4535 | 13.2922 | 5.75E-07 | ABCA13 | novel | GGT |
| 7 | 48269002 | T | A | 51.3516 | 10.0021 | 2.84E-07 | ABCA13 | novel | ALT |
| 7 | 48273223 | T | A | 24.1886 | 4.26088 | 1.37E-08 | ABCA13 | rs199793862 | AST |
| 7 | 48275691 | A | T | 45.8063 | 6.0256 | 2.92E-14 | ABCA13 | novel | AST |
| 7 | 48279282 | T | TA | 23.2497 | 4.41583 | 1.40E-07 | ABCA13 | novel | TBIL |
| 7 | 55978384 | C | A | 51.1625 | 5.94668 | 7.75E-18 | NIPSNAP2 | rs765376797 | AP |
| 7 | 65974701 | G | A | 26.6866 | 3.68995 | 4.76E-13 | GUSB | rs121918185 | AST |
| 7 | 65979909 | C | T | 24.292 | 4.41591 | 3.78E-08 | GUSB | novel | TBIL |
| 7 | 73683380 | C | T | 223.022 | 14.1409 | 5.08E-56 | DNAJC30 | rs903912935 | ALT |
| 7 | 73683380 | C | T | 270.365 | 10.429 | 4.62E-148 | DNAJC30 | rs903912935 | AST |
| 7 | 73683380 | C | T | 218.956 | 42.0323 | 1.90E-07 | DNAJC30 | rs903912935 | GGT |
| 7 | 73737346 | C | A | 78.7419 | 14.1445 | 2.59E-08 | ABHD11 | novel | ALT |
| 7 | 73737346 | C | A | 51.5876 | 10.437 | 7.71E-07 | ABHD11 | novel | AST |
| 7 | 75953987 | A | G | 44.0278 | 4.41567 | 2.06E-23 | POR | novel | TBIL |
| 7 | 75980549 | C | T | 359.252 | 25.9166 | 1.10E-43 | POR | novel | AP |
| 7 | 80672018 | G | GGACATACTTGGATATTGAACCTGTAAGAAAACACCTTATTGATCTGATTT | 30.2211 | 5.52672 | 4.55E-08 | CD36 | rs780002632 | AP |
| 7 | 87391686 | C | T | 139.273 | 14.1446 | 7.15E-23 | CROT | rs200447525 | ALT |
| 7 | 87391686 | C | T | 62.0852 | 10.4377 | 2.71E-09 | CROT | rs200447525 | AST |
| 7 | 87566243 | T | C | 187.111 | 18.796 | 2.42E-23 | ABCB1 | rs776202744 | GGT |
| 7 | 90372185 | A | C | 195.676 | 18.3276 | 1.32E-26 | GTPBP10 | novel | AP |
| 7 | 92518022 | A | AG | 292.565 | 42.036 | 3.41E-12 | PEX1 | novel | GGT |
| 7 | 95412419 | A | T | 23.3332 | 4.41589 | 1.27E-07 | PON2 | novel | TBIL |
| 7 | 95587798 | G | A | 128.034 | 25.9216 | 7.84E-07 | PDK4 | novel | AP |
| 7 | 96296897 | C | G | 217.874 | 14.1418 | 1.53E-53 | SLC25A13 | novel | ALT |
| 7 | 96296897 | C | G | 135.053 | 10.4361 | 2.69E-38 | SLC25A13 | novel | AST |
| 7 | 100721653 | C | T | 75.2321 | 14.9659 | 4.99E-07 | EPO | novel | AP |
| 7 | 100721653 | C | T | 165.413 | 24.2669 | 9.35E-12 | EPO | novel | GGT |
| 7 | 100892371 | G | A | 22.3661 | 4.41591 | 4.09E-07 | ACHE | novel | TBIL |
| 7 | 100892579 | G | T | 32.7532 | 6.3257 | 2.25E-07 | ACHE | novel | ALT |
| 7 | 100892579 | G | T | 24.8103 | 4.66756 | 1.06E-07 | ACHE | novel | AST |
| 7 | 101244959 | C | T | 486.631 | 25.9154 | 1.24E-78 | FIS1 | novel | AP |
| 7 | 107701825 | A | G | 88.7868 | 14.1448 | 3.46E-10 | SLC26A4 | novel | ALT |
| 7 | 107701825 | A | G | 68.0325 | 10.4353 | 7.06E-11 | SLC26A4 | novel | AST |
| 7 | 107701825 | A | G | 226.202 | 42.0403 | 7.43E-08 | SLC26A4 | novel | GGT |
| 7 | 107712587 | G | T | 33.1445 | 5.77454 | 9.49E-09 | SLC26A4 | novel | ALT |
| 7 | 116757541 | T | G | 261.673 | 42.0376 | 4.83E-10 | MET | novel | GGT |
| 7 | 116778931 | C | T | 102.791 | 14.1442 | 3.67E-13 | MET | novel | ALT |
| 7 | 117509144 | T | G | 178.957 | 14.1433 | 1.09E-36 | CFTR | novel | ALT |
| 7 | 117509144 | T | G | 75.8414 | 10.4174 | 3.34E-13 | CFTR | novel | AST |
| 7 | 117587811 | C | T | 8.02207 | 1.10645 | 4.17E-13 | CFTR | rs74597325 | AST |
| 7 | 117665466 | C | CA | 9.92982 | 1.66882 | 2.68E-09 | CFTR | rs397508685 | TBIL |
| 7 | 122079707 | T | C | 36.3107 | 4.41588 | 1.99E-16 | AASS | novel | TBIL |
| 7 | 127588547 | C | T | 28.8414 | 4.41585 | 6.53E-11 | ARF5 | novel | TBIL |
| 7 | 128821883 | G | T | 111.266 | 10.4358 | 1.55E-26 | CCDC136 | novel | AST |
| 7 | 134451741 | G | A | 98.1907 | 14.1454 | 3.88E-12 | AKR1B1 | novel | ALT |
| 7 | 134451741 | G | A | 71.257 | 10.4375 | 8.68E-12 | AKR1B1 | novel | AST |
| 7 | 140674123 | G | GA | 39.3195 | 5.34604 | 1.91E-13 | ADCK2 | rs775731411 | ALT |
| 7 | 140674123 | G | GA | 30.5795 | 3.94468 | 9.06E-15 | ADCK2 | rs775731411 | AST |
| 7 | 140721710 | G | A | 51.0262 | 10.0018 | 3.37E-07 | BRAF | novel | ALT |
| 7 | 151056750 | C | A | 24.7874 | 4.4159 | 1.99E-08 | CDK5 | novel | TBIL |
| 8 | 2129233 | G | T | 184.429 | 24.2693 | 2.98E-14 | MYOM2 | novel | GGT |
| 8 | 2144668 | T | A | 56.085 | 10.4369 | 7.72E-08 | MYOM2 | novel | AST |
| 8 | 11847764 | T | C | 48.6879 | 8.16638 | 2.49E-09 | CTSB | novel | ALT |
| 8 | 11847764 | T | C | 47.735 | 6.02557 | 2.34E-15 | CTSB | novel | AST |
| 8 | 11853328 | C | T | 154.24 | 14.1434 | 1.09E-27 | CTSB | novel | ALT |
| 8 | 11853328 | C | T | 114.235 | 10.436 | 6.99E-28 | CTSB | novel | AST |
| 8 | 17754433 | G | A | 58.6957 | 10.0017 | 4.40E-09 | MTUS1 | novel | ALT |
| 8 | 22513433 | G | A | 54.5854 | 10.4371 | 1.70E-07 | PPP3CC | novel | AST |
| 8 | 22532224 | G | A | 28.8978 | 4.41596 | 6.00E-11 | PPP3CC | novel | TBIL |
| 8 | 23022932 | C | T | 203.283 | 29.7206 | 7.94E-12 | TNFRSF10B | rs375688257 | GGT |
| 8 | 23028330 | C | T | 8.36794 | 1.53436 | 4.94E-08 | TNFRSF10B | rs41308110 | GGT |
| 8 | 23154872 | A | G | 168.495 | 29.7208 | 1.44E-08 | TNFRSF10D | novel | GGT |
| 8 | 27522479 | C | CATGG | 212.099 | 42.0322 | 4.51E-07 | EPHX2 | novel | GGT |
| 8 | 27525415 | C | T | 12.6529 | 2.20794 | 1.00E-08 | EPHX2 | novel | TBIL |
| 8 | 30703091 | A | T | 74.9008 | 14.1447 | 1.19E-07 | GSR | novel | ALT |
| 8 | 30703091 | A | T | 85.8883 | 10.4365 | 1.88E-16 | GSR | novel | AST |
| 8 | 30703091 | A | T | 356.742 | 42.0304 | 2.11E-17 | GSR | novel | GGT |
| 8 | 30793958 | C | CATTG | 146.828 | 25.9214 | 1.48E-08 | PPP2CB | novel | AP |
| 8 | 30793958 | C | CATTG | 330.019 | 42.0304 | 4.11E-15 | PPP2CB | novel | GGT |
| 8 | 42319403 | C | T | 90.9508 | 18.3294 | 6.98E-07 | IKBKB | rs759599047 | AP |
| 8 | 42319403 | C | T | 464.436 | 29.7136 | 4.68E-55 | IKBKB | rs759599047 | GGT |
| 8 | 42398865 | G | GT | 335.813 | 42.0302 | 1.36E-15 | VDAC3 | novel | GGT |
| 8 | 73976309 | T | TGG | 180.645 | 29.721 | 1.22E-09 | TMEM70 | novel | GGT |
| 8 | 73976385 | C | CT | 15.0589 | 2.94945 | 3.30E-07 | TMEM70 | novel | ALT |
| 8 | 81447313 | C | T | 95.5809 | 14.1447 | 1.41E-11 | PMP2 | novel | ALT |
| 8 | 81447313 | C | T | 264.747 | 25.9187 | 1.72E-24 | PMP2 | novel | AP |
| 8 | 81447313 | C | T | 130.88 | 10.4357 | 4.49E-36 | PMP2 | novel | AST |
| 8 | 81447313 | C | T | 618.695 | 42.0248 | 4.78E-49 | PMP2 | novel | GGT |
| 8 | 90060287 | C | G | 304.584 | 42.0311 | 4.28E-13 | CALB1 | novel | GGT |
| 8 | 95048455 | A | C | 206.011 | 29.7218 | 4.18E-12 | NDUFAF6 | novel | GGT |
| 8 | 99103087 | G | T | 22.4101 | 4.4159 | 3.88E-07 | VPS13B | novel | TBIL |
| 8 | 99134686 | A | T | 57.0879 | 9.16473 | 4.70E-10 | VPS13B | rs942483537 | AP |
| 8 | 99467566 | C | T | 64.5046 | 10.5824 | 1.09E-09 | VPS13B | rs140353201 | AP |
| 8 | 99501699 | C | T | 291.227 | 42.032 | 4.25E-12 | VPS13B | novel | GGT |
| 8 | 99507812 | C | T | 24.0751 | 4.41588 | 4.99E-08 | VPS13B | novel | TBIL |
| 8 | 99853931 | C | G | 33.3531 | 4.41571 | 4.25E-14 | VPS13B | novel | TBIL |
| 8 | 99875479 | C | CAA | 57.2623 | 4.41528 | 1.86E-38 | VPS13B | novel | TBIL |
| 8 | 102238729 | A | AC | 128.788 | 24.2676 | 1.11E-07 | RRM2B | novel | GGT |
| 8 | 108479004 | A | G | 55.0529 | 6.32617 | 3.26E-18 | EMC2 | novel | ALT |
| 8 | 108479004 | A | G | 47.2338 | 4.66752 | 4.55E-24 | EMC2 | novel | AST |
| 8 | 109607947 | T | C | 56.8071 | 10.437 | 5.25E-08 | SYBU | novel | AST |
| 8 | 124549760 | G | C | 37.6372 | 2.08671 | 1.07E-72 | NDUFB9 | novel | AST |
| 8 | 143329348 | A | C | 97.9673 | 18.8043 | 1.89E-07 | TOP1MT | rs773764755 | GGT |
| 8 | 143923535 | G | A | 307.408 | 42.0309 | 2.60E-13 | PLEC | novel | GGT |
| 8 | 143935022 | C | T | 60.502 | 10.4368 | 6.76E-09 | PLEC | novel | AST |
| 8 | 144098864 | A | G | 146.075 | 10.4364 | 1.67E-44 | SHARPIN | rs764880938 | AST |
| 8 | 144326436 | C | T | 144.717 | 25.9214 | 2.37E-08 | DGAT1 | novel | AP |
| 8 | 144512014 | C | CT | 161.611 | 25.9215 | 4.53E-10 | RECQL4 | novel | AP |
| 8 | 144512014 | C | CT | 473.491 | 42.0276 | 1.95E-29 | RECQL4 | novel | GGT |
| 8 | 144515213 | C | T | 73.9798 | 14.145 | 1.70E-07 | RECQL4 | novel | ALT |
| 8 | 144515213 | C | T | 78.7273 | 10.4369 | 4.60E-14 | RECQL4 | novel | AST |
| 8 | 144524678 | C | G | 77.3056 | 14.9659 | 2.40E-07 | LRRC24 | novel | AP |
| 8 | 144524678 | C | G | 289.876 | 24.264 | 6.84E-33 | LRRC24 | novel | GGT |
| 9 | 5080656 | C | T | 129.874 | 24.2676 | 8.71E-08 | JAK2 | rs769318116 | GGT |
| 9 | 37428574 | T | A | 24.8854 | 4.26037 | 5.19E-09 | GRHPR | rs180177313 | AST |
| 9 | 37428574 | T | A | 137.271 | 17.1608 | 1.26E-15 | GRHPR | rs180177313 | GGT |
| 9 | 77228254 | C | T | 23.4926 | 4.66764 | 4.83E-07 | VPS13A | novel | AST |
| 9 | 77339648 | A | G | 52.3195 | 10.0019 | 1.69E-07 | VPS13A | rs543745542 | ALT |
| 9 | 95876006 | A | G | 0.359819 | 0.0608155 | 3.29E-09 | ERCC6L2 | rs690528 | AP |
| 9 | 95928166 | G | GT | 97.592 | 18.799 | 2.09E-07 | ERCC6L2 | novel | GGT |
| 9 | 96012934 | A | T | 368.831 | 42.0297 | 1.71E-18 | ERCC6L2 | novel | GGT |
| 9 | 104768957 | T | TA | 65.9575 | 10.4368 | 2.62E-10 | NIPSNAP3B | novel | AST |
| 9 | 104768957 | T | TA | 250.907 | 42.0318 | 2.38E-09 | NIPSNAP3B | novel | GGT |
| 9 | 104770914 | G | T | 88.47 | 17.1607 | 2.53E-07 | NIPSNAP3B | novel | GGT |
| 9 | 117714510 | C | G | 420.066 | 42.0314 | 1.63E-23 | TLR4 | rs199962793 | GGT |
| 9 | 121286186 | G | T | 128.202 | 24.2766 | 1.29E-07 | GSN | novel | GGT |
| 9 | 121324554 | G | A | 45.4509 | 7.38086 | 7.38E-10 | GSN | rs897058765 | AST |
| 9 | 128224250 | G | A | 504.404 | 42.0264 | 3.51E-33 | DNM1 | novel | GGT |
| 9 | 128325229 | C | T | 72.6008 | 6.02486 | 1.96E-33 | COQ4 | novel | AST |
| 9 | 128325229 | C | T | 129.49 | 21.0159 | 7.21E-10 | COQ4 | novel | GGT |
| 9 | 137462728 | C | T | 25.2231 | 2.08718 | 1.29E-33 | PNPLA7 | rs372947422 | AST |
| 9 | 137552188 | G | A | 125.415 | 21.0162 | 2.41E-09 | MRPL41 | novel | GGT |
| 10 | 11747398 | A | G | 101.8 | 15.8871 | 1.48E-10 | ECHDC3 | rs201684812 | GGT |
| 10 | 12089221 | C | G | 33.9776 | 6.02578 | 1.71E-08 | DHTKD1 | novel | AST |
| 10 | 12107995 | C | T | 156.709 | 25.9212 | 1.49E-09 | DHTKD1 | rs745432268 | AP |
| 10 | 12118814 | T | G | 96.3036 | 18.7977 | 3.01E-07 | DHTKD1 | novel | GGT |
| 10 | 17235391 | T | C | 73.8888 | 14.1446 | 1.75E-07 | VIM | novel | ALT |
| 10 | 27046494 | A | T | 14.8327 | 1.55585 | 1.53E-21 | ANKRD26 | novel | AST |
| 10 | 27120509 | G | GT | 75.8316 | 14.9659 | 4.04E-07 | YME1L1 | novel | AP |
| 10 | 30313827 | G | A | 58.1051 | 10.4369 | 2.59E-08 | MTPAP | novel | AST |
| 10 | 30313827 | G | A | 477.234 | 42.0271 | 7.04E-30 | MTPAP | novel | GGT |
| 10 | 101787363 | C | T | 123.274 | 14.1438 | 2.90E-18 | OGA | novel | ALT |
| 10 | 101799457 | T | C | 71.2296 | 14.1448 | 4.76E-07 | OGA | novel | ALT |
| 10 | 101799457 | T | C | 51.7875 | 10.4371 | 6.98E-07 | OGA | novel | AST |
| 10 | 102379331 | C | T | 72.0095 | 8.19674 | 1.57E-18 | GBF1 | rs370874397 | AP |
| 10 | 102411776 | G | A | 72.6041 | 10.0014 | 3.90E-13 | PSD | rs764432848 | ALT |
| 10 | 102411776 | G | A | 110.479 | 7.3783 | 1.13E-50 | PSD | rs764432848 | AST |
| 10 | 102411776 | G | A | 249.061 | 29.7198 | 5.29E-17 | PSD | rs764432848 | GGT |
| 10 | 102412430 | G | A | 152.238 | 29.7212 | 3.02E-07 | PSD | rs374125352 | GGT |
| 10 | 102729809 | G | A | 23.2936 | 3.12241 | 8.66E-14 | SFXN2 | novel | TBIL |
| 10 | 102837360 | A | G | 15.886 | 2.95018 | 7.26E-08 | CYP17A1 | novel | ALT |
| 10 | 102837360 | A | G | 15.6122 | 2.17682 | 7.40E-13 | CYP17A1 | novel | AST |
| 10 | 112157247 | A | T | 39.0872 | 4.41603 | 8.69E-19 | GPAM | novel | TBIL |
| 10 | 113692071 | G | C | 154.24 | 14.1434 | 1.09E-27 | CASP7 | novel | ALT |
| 10 | 113692071 | G | C | 114.235 | 10.436 | 6.99E-28 | CASP7 | novel | AST |
| 10 | 122275100 | G | A | 88.4301 | 10.4364 | 2.39E-17 | BTBD16 | novel | AST |
| 10 | 122510131 | C | T | 24.0001 | 4.6676 | 2.72E-07 | HTRA1 | rs768243150 | AST |
| 10 | 122514189 | A | G | 352.634 | 42.031 | 4.88E-17 | HTRA1 | novel | GGT |
| 10 | 125794980 | T | C | 19.8852 | 3.12244 | 1.91E-10 | UROS | rs150039654 | TBIL |
| 10 | 125836854 | A | C | 215.502 | 42.0324 | 2.94E-07 | DHX32 | novel | GGT |
| 10 | 125839120 | G | A | 26.4437 | 4.41585 | 2.12E-09 | DHX32 | rs771082972 | TBIL |
| 10 | 128104573 | G | A | 71.2263 | 10.0015 | 1.07E-12 | MKI67 | novel | ALT |
| 11 | 233389 | T | C | 83.2569 | 14.1444 | 3.95E-09 | SIRT3 | novel | ALT |
| 11 | 822547 | C | T | 78.2074 | 14.1445 | 3.22E-08 | PNPLA2 | novel | ALT |
| 11 | 6390640 | G | T | 9.12679 | 1.84518 | 7.57E-07 | SMPD1 | novel | AST |
| 11 | 6393624 | T | C | 113.015 | 14.1439 | 1.35E-15 | SMPD1 | novel | ALT |
| 11 | 6393624 | T | C | 96.0892 | 10.4363 | 3.36E-20 | SMPD1 | novel | AST |
| 11 | 6393624 | T | C | 246.623 | 42.032 | 4.43E-09 | SMPD1 | novel | GGT |
| 11 | 6683135 | GC | G | 24.914 | 4.73316 | 1.41E-07 | MRPL17 | novel | AP |
| 11 | 7665514 | C | T | 33.6648 | 4.26523 | 2.96E-15 | CYB5R2 | rs75978231 | ALT |
| 11 | 17410539 | G | A | 88.3552 | 14.1444 | 4.20E-10 | ABCC8 | novel | ALT |
| 11 | 17410539 | G | A | 84.647 | 10.4365 | 5.05E-16 | ABCC8 | novel | AST |
| 11 | 17428288 | C | T | 79.973 | 10.4365 | 1.82E-14 | ABCC8 | novel | AST |
| 11 | 27055496 | T | A | 101.504 | 14.1442 | 7.17E-13 | BBOX1 | rs975719573 | ALT |
| 11 | 27055496 | T | A | 87.0331 | 10.4364 | 7.49E-17 | BBOX1 | rs975719573 | AST |
| 11 | 28328093 | A | G | 100.304 | 14.1443 | 1.33E-12 | METTL15 | novel | ALT |
| 11 | 28328093 | A | G | 60.6283 | 10.4369 | 6.29E-09 | METTL15 | novel | AST |
| 11 | 35214830 | C | A | 22.0934 | 3.1217 | 1.47E-12 | CD44 | rs371386627 | TBIL |
| 11 | 35301592 | G | A | 224.833 | 42.0319 | 8.84E-08 | SLC1A2 | novel | GGT |
| 11 | 36573728 | C | T | 26.2794 | 5.00087 | 1.48E-07 | RAG1 | rs773929270 | ALT |
| 11 | 45902418 | G | A | 40.4889 | 4.41666 | 4.87E-20 | MAPK8IP1 | rs756729012 | TBIL |
| 11 | 46543146 | G | A | 18.8366 | 3.24506 | 6.45E-09 | AMBRA1 | rs747889675 | ALT |
| 11 | 46543146 | G | A | 13.4297 | 2.39445 | 2.04E-08 | AMBRA1 | rs747889675 | AST |
| 11 | 57712809 | T | C | 293.951 | 14.1378 | 5.73E-96 | TMX2 | novel | ALT |
| 11 | 57712809 | T | C | 345.245 | 10.4237 | 3.01E-240 | TMX2 | novel | AST |
| 11 | 57712809 | T | C | 32.8149 | 4.41575 | 1.08E-13 | TMX2 | novel | TBIL |
| 11 | 58955723 | T | TA | 289.965 | 42.0313 | 5.25E-12 | GLYATL1 | novel | GGT |
| 11 | 61796130 | G | A | 40.3079 | 7.38021 | 4.72E-08 | FEN1 | rs757420433 | AST |
| 11 | 61796501 | A | AAAGG | 234.566 | 29.72 | 2.97E-15 | FEN1 | novel | GGT |
| 11 | 61796502 | T | G | 234.566 | 29.72 | 2.97E-15 | FEN1 | novel | GGT |
| 11 | 62575239 | T | C | 113.338 | 18.3298 | 6.28E-10 | TUT1 | novel | AP |
| 11 | 62575239 | T | C | 416.087 | 29.7165 | 1.55E-44 | TUT1 | novel | GGT |
| 11 | 62578559 | A | C | 28.1863 | 4.41587 | 1.74E-10 | TUT1 | novel | TBIL |
| 11 | 62591519 | C | G | 106.251 | 14.1442 | 5.83E-14 | TUT1 | novel | ALT |
| 11 | 62591519 | C | G | 51.8308 | 10.437 | 6.84E-07 | TUT1 | novel | AST |
| 11 | 64898169 | T | C | 12.6286 | 2.54955 | 7.30E-07 | ATG2A | novel | TBIL |
| 11 | 64907531 | T | A | 83.221 | 14.1446 | 4.02E-09 | ATG2A | novel | ALT |
| 11 | 65014209 | T | A | 271.962 | 42.0325 | 9.79E-11 | ARL2 | novel | GGT |
| 11 | 66872160 | C | T | 130.694 | 14.1436 | 2.46E-20 | PC | novel | ALT |
| 11 | 66872160 | C | T | 399.829 | 25.9151 | 1.09E-53 | PC | novel | AP |
| 11 | 66872160 | C | T | 130.854 | 10.4353 | 4.60E-36 | PC | novel | AST |
| 11 | 66872160 | C | T | 580.888 | 42.024 | 1.90E-43 | PC | novel | GGT |
| 11 | 68413921 | G | GT | 8.4488 | 1.69322 | 6.05E-07 | LRP5 | rs886043590 | AST |
| 11 | 68793358 | C | T | 63.4818 | 6.02528 | 5.95E-26 | CPT1A | novel | AST |
| 11 | 68796900 | G | A | 72.1989 | 10.0014 | 5.25E-13 | CPT1A | rs779893091 | ALT |
| 11 | 68796900 | G | A | 39.1422 | 7.38001 | 1.13E-07 | CPT1A | rs779893091 | AST |
| 11 | 68896526 | G | A | 25.8343 | 5.21851 | 7.40E-07 | MRPL21 | rs992841927 | AST |
| 11 | 72590191 | C | A | 78.0392 | 14.1444 | 3.44E-08 | PDE2A | novel | ALT |
| 11 | 72590191 | C | A | 87.4157 | 10.4356 | 5.46E-17 | PDE2A | novel | AST |
| 11 | 72590191 | C | A | 295.674 | 42.0313 | 2.00E-12 | PDE2A | novel | GGT |
| 11 | 73844805 | A | G | 126.482 | 14.1437 | 3.81E-19 | MRPL48 | novel | ALT |
| 11 | 73844805 | A | G | 173.841 | 10.4339 | 2.63E-62 | MRPL48 | novel | AST |
| 11 | 74005729 | C | T | 246.466 | 42.0318 | 4.53E-09 | UCP3 | rs777965664 | GGT |
| 11 | 85656328 | C | T | 32.4706 | 6.32573 | 2.85E-07 | TMEM126A | novel | ALT |
| 11 | 85656328 | C | T | 23.2728 | 4.6676 | 6.17E-07 | TMEM126A | novel | AST |
| 11 | 85656328 | C | T | 110.059 | 18.7974 | 4.77E-09 | TMEM126A | novel | GGT |
| 11 | 86457728 | C | T | 76.9722 | 14.1446 | 5.28E-08 | ME3 | novel | ALT |
| 11 | 89444233 | C | A | 131.846 | 24.2678 | 5.55E-08 | NOX4 | novel | GGT |
| 11 | 108133852 | C | CA | 53.199 | 10.437 | 3.45E-07 | ACAT1 | novel | AST |
| 11 | 108250705 | C | T | 126.717 | 10.4388 | 6.65E-34 | ATM | rs866521873 | AST |
| 11 | 108250705 | C | T | 520.005 | 42.0615 | 4.20E-35 | ATM | rs866521873 | GGT |
| 11 | 108271147 | G | A | 220.393 | 42.0324 | 1.58E-07 | ATM | rs587781558 | GGT |
| 11 | 108284475 | T | C | 547.244 | 42.0251 | 9.35E-39 | ATM | novel | GGT |
| 11 | 108304732 | C | T | 76.0739 | 10.0015 | 2.83E-14 | ATM | rs754562056 | ALT |
| 11 | 108304732 | C | T | 44.8871 | 7.38002 | 1.19E-09 | ATM | rs754562056 | AST |
| 11 | 108319953 | G | C | 394.604 | 42.0208 | 5.99E-21 | ATM | novel | GGT |
| 11 | 108331516 | A | T | 296.952 | 25.9184 | 2.19E-30 | ATM | novel | AP |
| 11 | 108331516 | A | T | 324.065 | 42.0305 | 1.26E-14 | ATM | novel | GGT |
| 11 | 108331877 | A | C | 43.7442 | 7.38019 | 3.08E-09 | ATM | rs587779866 | AST |
| 11 | 108354848 | C | T | 31.9771 | 6.02583 | 1.12E-07 | ATM | novel | AST |
| 11 | 112086687 | G | A | 23.1915 | 4.41587 | 1.51E-07 | TIMM8B | rs914610565 | TBIL |
| 11 | 112086723 | T | C | 94.1631 | 7.37885 | 2.74E-37 | TIMM8B | novel | AST |
| 11 | 112086723 | T | C | 537.132 | 29.7108 | 5.00E-73 | TIMM8B | novel | GGT |
| 11 | 112214945 | G | T | 281.582 | 42.0311 | 2.10E-11 | BCO2 | rs766154406 | GGT |
| 11 | 119045812 | A | G | 73.7079 | 12.9614 | 1.30E-08 | HYOU1 | novel | AP |
| 11 | 119046700 | G | A | 28.8979 | 4.41581 | 5.99E-11 | HYOU1 | novel | TBIL |
| 11 | 119093022 | G | T | 17.4769 | 3.12249 | 2.18E-08 | HMBS | novel | TBIL |
| 11 | 119285032 | C | T | 24.0263 | 4.41589 | 5.30E-08 | CBL | novel | TBIL |
| 11 | 119299780 | A | G | 25.0695 | 4.41587 | 1.37E-08 | CBL | novel | TBIL |
| 12 | 6981044 | G | A | 24.278 | 4.41588 | 3.85E-08 | LPCAT3 | novel | TBIL |
| 12 | 7191360 | T | C | 215.851 | 42.0328 | 2.82E-07 | PEX5 | novel | GGT |
| 12 | 7324561 | C | T | 84.14 | 9.16411 | 4.27E-20 | ACSM4 | rs936896432 | AP |
| 12 | 21842339 | G | A | 290.925 | 42.0316 | 4.47E-12 | ABCC9 | novel | GGT |
| 12 | 21848204 | G | A | 190.918 | 29.7207 | 1.33E-10 | ABCC9 | novel | GGT |
| 12 | 21910829 | C | CAG | 236.369 | 42.0323 | 1.87E-08 | ABCC9 | novel | GGT |
| 12 | 24901843 | C | A | 111.593 | 14.144 | 3.03E-15 | BCAT1 | novel | ALT |
| 12 | 26443623 | G | A | 96.0597 | 14.1444 | 1.11E-11 | ITPR2 | novel | ALT |
| 12 | 26443623 | G | A | 65.4975 | 10.4369 | 3.49E-10 | ITPR2 | novel | AST |
| 12 | 26443623 | G | A | 294.63 | 42.0314 | 2.39E-12 | ITPR2 | novel | GGT |
| 12 | 26954143 | A | G | -13.9275 | 2.58388 | 7.04E-08 | FGFR1OP2 | novel | ALB |
| 12 | 26954143 | A | G | 318.627 | 25.9198 | 1.00E-34 | FGFR1OP2 | novel | AP |
| 12 | 26954143 | A | G | 202.856 | 10.4342 | 3.75E-84 | FGFR1OP2 | novel | AST |
| 12 | 26954143 | A | G | 878.027 | 42.0181 | 6.47E-97 | FGFR1OP2 | novel | GGT |
| 12 | 26954143 | A | G | 47.3853 | 4.41522 | 7.24E-27 | FGFR1OP2 | novel | TBIL |
| 12 | 27716290 | G | A | 30.9549 | 6.02592 | 2.79E-07 | MRPS35 | novel | AST |
| 12 | 27716302 | T | C | 11.4918 | 2.20802 | 1.95E-07 | MRPS35 | rs1041969141 | TBIL |
| 12 | 30715038 | A | AT | 34.2038 | 4.41574 | 9.51E-15 | CAPRIN2 | novel | TBIL |
| 12 | 30724453 | T | C | 56.647 | 11.2336 | 4.59E-07 | CAPRIN2 | novel | GGT |
| 12 | 30730244 | G | A | 354.185 | 42.034 | 3.58E-17 | CAPRIN2 | novel | GGT |
| 12 | 32737153 | C | T | 93.817 | 14.8606 | 2.74E-10 | DNM1L | novel | GGT |
| 12 | 32754028 | T | TC | 165.591 | 24.2668 | 8.88E-12 | YARS2 | novel | GGT |
| 12 | 32754032 | G | GCT | 165.591 | 24.2668 | 8.88E-12 | YARS2 | novel | GGT |
| 12 | 47899836 | C | A | 33.7063 | 2.89491 | 2.51E-31 | VDR | rs371796666 | AST |
| 12 | 48108095 | A | G | 34.6557 | 4.41571 | 4.23E-15 | PFKM | novel | TBIL |
| 12 | 48840093 | G | A | 32.152 | 4.4158 | 3.32E-13 | DDX23 | novel | TBIL |
| 12 | 49186372 | G | A | 266.877 | 42.0316 | 2.16E-10 | TUBA1A | novel | GGT |
| 12 | 52056162 | G | A | 78.5635 | 14.1445 | 2.79E-08 | NR4A1 | novel | ALT |
| 12 | 52056162 | G | A | 671.423 | 42.0208 | 1.88E-57 | NR4A1 | novel | GGT |
| 12 | 54295206 | G | C | 11.3228 | 1.2475 | 1.13E-19 | NFE2 | rs772682262 | AST |
| 12 | 56269538 | C | T | 115.062 | 8.6392 | 1.84E-40 | COQ10A | rs370149350 | AP |
| 12 | 56477939 | C | A | 33.76 | 4.41578 | 2.09E-14 | GLS2 | novel | TBIL |
| 12 | 57764376 | A | C | 25.1231 | 3.12238 | 8.57E-16 | CYP27B1 | novel | TBIL |
| 12 | 57783283 | G | GGT | 55.7936 | 10.0018 | 2.43E-08 | TSFM | rs762424912 | ALT |
| 12 | 57783283 | G | GGT | 66.617 | 7.37962 | 1.77E-19 | TSFM | rs762424912 | AST |
| 12 | 57783283 | G | GGT | 165.569 | 29.722 | 2.54E-08 | TSFM | rs762424912 | GGT |
| 12 | 57953678 | C | T | 11.3064 | 1.80278 | 3.58E-10 | ATP23 | novel | TBIL |
| 12 | 64497165 | A | G | 215.166 | 29.7378 | 4.65E-13 | TBK1 | rs199605037 | GGT |
| 12 | 66138145 | T | C | 16.9349 | 3.12251 | 5.85E-08 | TMBIM4 | rs769509769 | TBIL |
| 12 | 68155400 | G | A | 33.9738 | 4.41575 | 1.43E-14 | IFNG | rs767503659 | TBIL |
| 12 | 71884914 | T | C | 42.8775 | 4.08286 | 8.54E-26 | TBC1D15 | rs376375651 | ALT |
| 12 | 71884914 | T | C | 45.2425 | 3.01223 | 5.63E-51 | TBC1D15 | rs376375651 | AST |
| 12 | 71923160 | C | T | 93.7645 | 14.1443 | 3.38E-11 | TBC1D15 | novel | ALT |
| 12 | 88194870 | C | T | 60.2438 | 7.37972 | 3.27E-16 | TMTC3 | novel | AST |
| 12 | 88194870 | C | T | 465.287 | 29.7138 | 2.99E-55 | TMTC3 | novel | GGT |
| 12 | 98712437 | T | G | 36.4362 | 6.32873 | 8.55E-09 | APAF1 | novel | ALT |
| 12 | 103841154 | C | T | 176.608 | 25.921 | 9.55E-12 | NT5DC3 | novel | AP |
| 12 | 103841154 | C | T | 239.966 | 42.0205 | 1.13E-08 | NT5DC3 | novel | GGT |
| 12 | 105030322 | A | G | 314.78 | 42.0335 | 6.96E-14 | ALDH1L2 | novel | GGT |
| 12 | 105034399 | C | G | 183.423 | 29.7214 | 6.77E-10 | ALDH1L2 | rs755299816 | GGT |
| 12 | 109171915 | G | A | 25.152 | 4.4158 | 1.23E-08 | ACACB | novel | TBIL |
| 12 | 109191647 | C | T | 53.2013 | 10.0022 | 1.04E-07 | ACACB | rs1018207163 | ALT |
| 12 | 111712523 | A | G | 24.2828 | 4.66757 | 1.97E-07 | ACAD10 | novel | AST |
| 12 | 111712523 | A | G | 124.65 | 18.797 | 3.33E-11 | ACAD10 | novel | GGT |
| 12 | 111729836 | G | A | 89.1079 | 14.8634 | 2.03E-09 | ACAD10 | rs199879065 | GGT |
| 12 | 111748336 | C | A | 99.7379 | 14.1443 | 1.77E-12 | ACAD10 | novel | ALT |
| 12 | 111749241 | C | T | 94.3309 | 14.145 | 2.58E-11 | ACAD10 | rs765418830 | ALT |
| 12 | 113320849 | C | T | 61.9364 | 10.4368 | 2.95E-09 | SLC8B1 | novel | AST |
| 12 | 117215326 | T | C | 18.6484 | 3.12247 | 2.34E-09 | NOS1 | novel | TBIL |
| 12 | 117247383 | C | G | 36.8496 | 5.21838 | 1.65E-12 | NOS1 | novel | AST |
| 12 | 119516691 | C | A | 21.0633 | 3.12249 | 1.53E-11 | CCDC60 | novel | TBIL |
| 12 | 120303669 | T | TCCTC | 179.028 | 25.921 | 4.97E-12 | SIRT4 | novel | AP |
| 12 | 120303669 | T | TCCTC | 502.683 | 42.0265 | 5.75E-33 | SIRT4 | novel | GGT |
| 12 | 120737875 | C | T | 1.33974 | 0.20884 | 1.41E-10 | ACADS | rs1800556 | GGT |
| 12 | 121184344 | C | A | 199.976 | 12.9577 | 1.02E-53 | P2RX7 | novel | AP |
| 12 | 121184344 | C | A | 184.361 | 21.0151 | 1.75E-18 | P2RX7 | novel | GGT |
| 12 | 121184344 | C | A | 12.2547 | 2.2079 | 2.85E-08 | P2RX7 | novel | TBIL |
| 13 | 23858859 | C | T | 77.5337 | 14.1446 | 4.22E-08 | MIPEP | novel | ALT |
| 13 | 23858859 | C | T | 52.4442 | 10.437 | 5.04E-07 | MIPEP | novel | AST |
| 13 | 24421315 | C | T | 261.853 | 42.0421 | 4.72E-10 | PARP4 | novel | GGT |
| 13 | 24431457 | G | GT | 17.3918 | 3.12254 | 2.55E-08 | PARP4 | novel | TBIL |
| 13 | 24447081 | G | A | 132.05 | 8.16413 | 7.96E-59 | PARP4 | novel | ALT |
| 13 | 24447081 | G | A | 131.923 | 14.965 | 1.20E-18 | PARP4 | novel | AP |
| 13 | 24447081 | G | A | 174.014 | 24.2667 | 7.46E-13 | PARP4 | novel | GGT |
| 13 | 24456429 | T | TC | 25.4993 | 4.41607 | 7.74E-09 | PARP4 | novel | TBIL |
| 13 | 27440196 | G | A | 26.4883 | 4.47291 | 3.18E-09 | MTIF3 | novel | ALT |
| 13 | 36335649 | G | C | 210.479 | 42.0322 | 5.51E-07 | SPART | novel | GGT |
| 13 | 41226569 | C | T | 65.9072 | 10.0016 | 4.41E-11 | MTRF1 | rs773078192 | ALT |
| 13 | 41226569 | C | T | 106.572 | 7.37844 | 2.82E-47 | MTRF1 | rs773078192 | AST |
| 13 | 41685236 | T | C | 12.4627 | 1.80277 | 4.75E-12 | VWA8 | novel | TBIL |
| 13 | 46227993 | T | A | 23.7787 | 4.41588 | 7.26E-08 | LRRC63 | novel | TBIL |
| 13 | 47968606 | G | T | 102.624 | 10.0008 | 1.06E-24 | SUCLA2 | novel | ALT |
| 13 | 47968606 | G | T | 57.2967 | 7.37978 | 8.25E-15 | SUCLA2 | novel | AST |
| 13 | 47973314 | T | C | 11.2349 | 2.22528 | 4.45E-07 | SUCLA2 | novel | AST |
| 13 | 100425674 | G | A | 6.76526 | 1.22477 | 3.32E-08 | PCCA | rs776496862 | TBIL |
| 13 | 113633183 | C | T | 8.83817 | 1.56126 | 1.51E-08 | TFDP1 | rs141171684 | TBIL |
| 14 | 20996271 | C | G | 34.0192 | 4.41542 | 1.31E-14 | METTL17 | novel | TBIL |
| 14 | 23402463 | C | T | 31.9434 | 4.41576 | 4.70E-13 | MYH6 | novel | TBIL |
| 14 | 23405099 | C | T | 148.563 | 29.7231 | 5.79E-07 | MYH6 | rs896503825 | GGT |
| 14 | 24098206 | G | A | 110.427 | 15.8864 | 3.63E-12 | PCK2 | rs770169595 | GGT |
| 14 | 24098388 | G | A | 19.3791 | 3.12247 | 5.43E-10 | PCK2 | rs749046501 | TBIL |
| 14 | 24339133 | C | CAAAGG | 81.9887 | 14.145 | 6.78E-09 | RIPK3 | novel | ALT |
| 14 | 24339133 | C | CAAAGG | 213.137 | 25.9211 | 2.00E-16 | RIPK3 | novel | AP |
| 14 | 24339133 | C | CAAAGG | 56.5703 | 10.4373 | 5.96E-08 | RIPK3 | novel | AST |
| 14 | 24339133 | C | CAAAGG | 400.807 | 42.0303 | 1.49E-21 | RIPK3 | novel | GGT |
| 14 | 31826628 | G | C | 26.1962 | 4.41573 | 2.99E-09 | NUBPL | rs775422270 | TBIL |
| 14 | 50265357 | C | T | 119.649 | 14.1464 | 2.73E-17 | L2HGDH | novel | ALT |
| 14 | 54413605 | G | C | 52.7533 | 9.40343 | 2.02E-08 | CDKN3 | novel | GGT |
| 14 | 63980684 | C | G | 164.753 | 29.7247 | 2.98E-08 | SYNE2 | novel | GGT |
| 14 | 64003323 | A | T | 128.924 | 25.9217 | 6.57E-07 | SYNE2 | novel | AP |
| 14 | 64022029 | G | A | 113.419 | 14.144 | 1.07E-15 | SYNE2 | novel | ALT |
| 14 | 64022029 | G | A | 81.407 | 10.4365 | 6.19E-15 | SYNE2 | novel | AST |
| 14 | 64098647 | G | A | 246.203 | 29.7024 | 1.15E-16 | SYNE2 | novel | GGT |
| 14 | 64141994 | G | A | 23.6366 | 4.41589 | 8.67E-08 | SYNE2 | novel | TBIL |
| 14 | 64249680 | C | T | 59.4234 | 7.37971 | 8.15E-16 | ESR2 | rs776592617 | AST |
| 14 | 64249680 | C | T | 268.979 | 29.7194 | 1.43E-19 | ESR2 | rs776592617 | GGT |
| 14 | 64282971 | G | C | 18.6314 | 3.12251 | 2.42E-09 | ESR2 | novel | TBIL |
| 14 | 68909359 | G | A | 132.792 | 25.9217 | 3.01E-07 | ACTN1 | novel | AP |
| 14 | 70051007 | C | T | 86.0053 | 7.07155 | 5.01E-34 | SLC8A3 | novel | ALT |
| 14 | 70051007 | C | T | 56.3433 | 5.21822 | 3.57E-27 | SLC8A3 | novel | AST |
| 14 | 70063930 | C | T | 23.4367 | 4.41596 | 1.11E-07 | SLC8A3 | novel | TBIL |
| 14 | 73148062 | C | T | 64.3278 | 10.4368 | 7.12E-10 | PSEN1 | novel | AST |
| 14 | 73573398 | C | CT | 74.2914 | 10.0052 | 1.13E-13 | ACOT2 | rs568865474 | ALT |
| 14 | 73950102 | C | T | 134.893 | 14.1435 | 1.47E-21 | COQ6 | rs750630720 | ALT |
| 14 | 74260851 | G | T | 72.1686 | 14.1446 | 3.36E-07 | VSX2 | novel | ALT |
| 14 | 74902200 | A | AG | 25.1078 | 4.41591 | 1.30E-08 | DLST | novel | TBIL |
| 14 | 89963507 | C | T | 67.996 | 10.5094 | 9.81E-11 | TDP1 | rs568810177 | GGT |
| 14 | 90780874 | C | T | 154.911 | 29.7214 | 1.87E-07 | TTC7B | novel | GGT |
| 14 | 90780907 | C | A | 38.5084 | 7.3801 | 1.81E-07 | TTC7B | novel | AST |
| 14 | 90786245 | G | A | 84.258 | 14.1445 | 2.57E-09 | TTC7B | novel | ALT |
| 14 | 90786245 | G | A | 79.8009 | 10.4366 | 2.07E-14 | TTC7B | novel | AST |
| 14 | 91170002 | G | A | 231.65 | 42.0321 | 3.56E-08 | DGLUCY | rs531687748 | GGT |
| 14 | 91196410 | C | T | 76.6013 | 14.1446 | 6.11E-08 | DGLUCY | novel | ALT |
| 14 | 95544006 | G | GT | 38.7709 | 7.38008 | 1.49E-07 | GLRX5 | novel | AST |
| 14 | 96331515 | G | A | 131.561 | 25.9217 | 3.87E-07 | ATG2B | novel | AP |
| 14 | 100292979 | G | A | 16.9701 | 3.12265 | 5.50E-08 | SLC25A29 | rs973408597 | TBIL |
| 14 | 100329446 | C | A | 48.7784 | 9.3989 | 2.11E-07 | SLC25A47 | rs565898992 | GGT |
| 14 | 103560657 | G | A | 33.4584 | 5.77452 | 6.87E-09 | BAG5 | novel | ALT |
| 14 | 104711008 | G | A | 139.865 | 18.3287 | 2.34E-14 | INF2 | novel | AP |
| 15 | 28265899 | G | A | 119.187 | 15.8917 | 6.40E-14 | HERC2 | rs148362399 | GGT |
| 15 | 34364241 | C | T | 247.273 | 25.9196 | 1.44E-21 | LPCAT4 | novel | AP |
| 15 | 34364241 | C | T | 369.418 | 42.0298 | 1.51E-18 | LPCAT4 | novel | GGT |
| 15 | 39770523 | T | A | 61.7167 | 7.37988 | 6.14E-17 | FSIP1 | novel | AST |
| 15 | 39770523 | T | A | 166.174 | 29.7221 | 2.26E-08 | FSIP1 | novel | GGT |
| 15 | 43520970 | A | C | 86.1044 | 14.1445 | 1.15E-09 | MAP1A | novel | ALT |
| 15 | 43520970 | A | C | 88.0091 | 10.4365 | 3.38E-17 | MAP1A | novel | AST |
| 15 | 43696096 | C | T | 168.601 | 29.7136 | 1.39E-08 | CKMT1A | novel | GGT |
| 15 | 45150702 | G | C | 102.023 | 18.7977 | 5.72E-08 | DUOX1 | novel | GGT |
| 15 | 45669953 | C | T | 74.3688 | 12.1339 | 8.85E-10 | SQOR | novel | GGT |
| 15 | 52112632 | C | T | 246.898 | 29.7198 | 9.80E-17 | BCL2L10 | rs200846236 | GGT |
| 15 | 60354215 | T | A | 255.533 | 42.0294 | 1.20E-09 | ANXA2 | novel | GGT |
| 15 | 63127050 | G | T | 15.4187 | 3.12235 | 7.89E-07 | LACTB | rs1019640287 | TBIL |
| 15 | 63263617 | T | G | 16.2655 | 2.53137 | 1.32E-10 | RAB8B | rs368594258 | AST |
| 15 | 64162986 | T | C | 46.5388 | 6.02577 | 1.14E-14 | PPIB | rs201472818 | AST |
| 15 | 64821077 | C | T | 64.3571 | 10.0016 | 1.24E-10 | PIF1 | rs747072711 | ALT |
| 15 | 73985478 | C | T | 71.1614 | 10.4368 | 9.22E-12 | STOML1 | novel | AST |
| 15 | 74023038 | G | GC | 145.749 | 25.9214 | 1.88E-08 | PML | novel | AP |
| 15 | 74023038 | G | GC | 292.484 | 42.0312 | 3.44E-12 | PML | novel | GGT |
| 15 | 74035637 | C | T | 51.9528 | 10.437 | 6.43E-07 | PML | rs778003774 | AST |
| 15 | 78160156 | T | C | 213.505 | 9.99679 | 3.77E-101 | IDH3A | novel | ALT |
| 15 | 78160156 | T | C | 112.208 | 7.37836 | 3.25E-52 | IDH3A | novel | AST |
| 15 | 78465250 | G | A | 190.522 | 14.1422 | 2.34E-41 | IREB2 | novel | ALT |
| 15 | 78465250 | G | A | 75.7699 | 10.4368 | 3.88E-13 | IREB2 | novel | AST |
| 15 | 78490762 | G | A | 235.438 | 25.92 | 1.06E-19 | IREB2 | novel | AP |
| 15 | 89316808 | G | GTAAA | 28.7768 | 4.4159 | 7.20E-11 | POLG | novel | TBIL |
| 15 | 101049667 | G | A | 85.1962 | 14.1445 | 1.71E-09 | LRRK1 | novel | ALT |
| 15 | 101049667 | G | A | 109.56 | 10.4359 | 8.86E-26 | LRRK1 | novel | AST |
| 15 | 101057051 | G | GTACT | 79.2987 | 14.966 | 1.17E-07 | LRRK1 | novel | AP |
| 16 | 670121 | A | G | 40.4428 | 6.02565 | 1.93E-11 | RHOT2 | novel | AST |
| 16 | 670121 | A | G | 189.988 | 24.2664 | 4.92E-15 | RHOT2 | novel | GGT |
| 16 | 1339161 | C | T | 5.09088 | 0.987454 | 2.53E-07 | BAIAP3 | novel | TBIL |
| 16 | 1347620 | A | C | 40.1794 | 5.65717 | 1.23E-12 | BAIAP3 | rs905972851 | AP |
| 16 | 1819177 | G | A | 145.934 | 14.1433 | 5.87E-25 | HAGH | rs758131317 | ALT |
| 16 | 1819177 | G | A | 73.7089 | 10.4367 | 1.64E-12 | HAGH | rs758131317 | AST |
| 16 | 2040039 | C | T | 141.709 | 14.1434 | 1.26E-23 | NTHL1 | novel | ALT |
| 16 | 2040039 | C | T | 148.753 | 25.9215 | 9.55E-09 | NTHL1 | novel | AP |
| 16 | 2040039 | C | T | 129.757 | 10.4354 | 1.73E-35 | NTHL1 | novel | AST |
| 16 | 2538113 | A | G | 19.5126 | 2.79056 | 2.71E-12 | PDPK1 | novel | AST |
| 16 | 3256587 | T | C | 44.971 | 7.37993 | 1.10E-09 | MEFV | novel | AST |
| 16 | 3690935 | G | A | 21.304 | 3.16287 | 1.63E-11 | TRAP1 | rs141984778 | ALT |
| 16 | 4382897 | T | G | 113.096 | 21.0163 | 7.40E-08 | VASN | novel | GGT |
| 16 | 4442266 | A | T | 112.625 | 14.1439 | 1.69E-15 | DNAJA3 | novel | ALT |
| 16 | 4442266 | A | T | 73.01 | 10.4366 | 2.65E-12 | DNAJA3 | novel | AST |
| 16 | 8774966 | G | A | 45.7767 | 4.66722 | 1.04E-22 | ABAT | novel | AST |
| 16 | 8779477 | A | C | 22.1613 | 4.41585 | 5.21E-07 | ABAT | novel | TBIL |
| 16 | 11676272 | C | T | 180.128 | 21.0151 | 1.03E-17 | SNN | rs755344205 | GGT |
| 16 | 15407975 | T | A | 186.523 | 29.7215 | 3.48E-10 | MPV17L | novel | GGT |
| 16 | 16115074 | C | T | 333.997 | 42.0305 | 1.92E-15 | ABCC1 | novel | GGT |
| 16 | 20471590 | G | A | 621.751 | 42.0226 | 1.61E-49 | ACSM2A | novel | GGT |
| 16 | 20559243 | G | A | 102.244 | 18.7975 | 5.35E-08 | ACSM2B | rs267604440 | GGT |
| 16 | 21267704 | C | A | 21.4527 | 3.16732 | 1.26E-11 | CRYM | rs145787995 | ALT |
| 16 | 21267704 | C | A | 12.203 | 2.33715 | 1.78E-07 | CRYM | rs145787995 | AST |
| 16 | 23552154 | C | T | 80.0488 | 5.77341 | 1.05E-43 | EARS2 | novel | ALT |
| 16 | 23552154 | C | T | 48.1001 | 4.26038 | 1.48E-29 | EARS2 | novel | AST |
| 16 | 23713142 | G | A | 38.5731 | 6.10994 | 2.74E-10 | ERN2 | novel | AP |
| 16 | 24219960 | G | A | 306.172 | 42.0308 | 3.24E-13 | PRKCB | novel | GGT |
| 16 | 25217234 | G | C | 119.401 | 18.3291 | 7.31E-11 | AQP8 | novel | AP |
| 16 | 28491673 | C | T | 10.6943 | 1.97486 | 6.12E-08 | CLN3 | rs375532645 | TBIL |
| 16 | 55479633 | G | T | 285.242 | 42.0312 | 1.15E-11 | MMP2 | novel | GGT |
| 16 | 55583096 | T | C | 72.013 | 13.292 | 6.04E-08 | LPCAT2 | novel | GGT |
| 16 | 58718246 | C | A | 206.282 | 29.7207 | 3.91E-12 | GOT2 | novel | GGT |
| 16 | 66531441 | C | T | 297.15 | 42.0314 | 1.55E-12 | TK2 | rs761860999 | GGT |
| 16 | 67174389 | C | T | 52.1824 | 10.5088 | 6.85E-07 | NOL3 | rs375650340 | GGT |
| 16 | 67930464 | G | A | 113.28 | 21.0162 | 7.04E-08 | CTRL | novel | GGT |
| 16 | 67930589 | C | T | 62.1534 | 10.0038 | 5.20E-10 | CTRL | rs756379071 | ALT |
| 16 | 67930589 | C | T | 16.0881 | 3.12296 | 2.58E-07 | CTRL | rs756379071 | TBIL |
| 16 | 71571604 | A | C | 120.533 | 14.1439 | 1.57E-17 | TAT | novel | ALT |
| 16 | 72788228 | G | A | 222.749 | 42.0322 | 1.16E-07 | ZFHX3 | novel | GGT |
| 16 | 72958003 | G | A | 22.1392 | 4.41592 | 5.35E-07 | ZFHX3 | novel | TBIL |
| 16 | 74676300 | C | A | 16.279 | 3.12258 | 1.86E-07 | MLKL | rs541991111 | TBIL |
| 16 | 74678933 | G | C | 89.2555 | 14.1444 | 2.79E-10 | MLKL | novel | ALT |
| 16 | 74678933 | G | C | 112.335 | 10.4358 | 5.11E-27 | MLKL | novel | AST |
| 16 | 75114235 | C | T | 210.447 | 42.0322 | 5.54E-07 | LDHD | novel | GGT |
| 16 | 75115196 | A | ACCT | 14.4026 | 2.55059 | 1.64E-08 | LDHD | rs777397715 | TBIL |
| 16 | 75634230 | A | G | 75.6568 | 6.02492 | 3.68E-36 | KARS1 | novel | AST |
| 16 | 75634230 | A | G | 321.076 | 24.2634 | 5.77E-40 | KARS1 | novel | GGT |
| 16 | 75641662 | C | A | 73.2607 | 14.1446 | 2.23E-07 | KARS1 | novel | ALT |
| 16 | 75641662 | C | A | 237.238 | 42.032 | 1.66E-08 | KARS1 | novel | GGT |
| 16 | 81084595 | C | CA | 45.7651 | 8.16622 | 2.09E-08 | GCSH | novel | ALT |
| 16 | 84124711 | A | C | 13.2318 | 2.54956 | 2.11E-07 | HSDL1 | novel | TBIL |
| 16 | 87403094 | G | GT | 40.0739 | 4.66739 | 9.04E-18 | MAP1LC3B | rs775812124 | AST |
| 16 | 88432703 | C | T | 127.869 | 14.1436 | 1.56E-19 | ZNF469 | novel | ALT |
| 16 | 88435292 | G | GA | 77.0579 | 14.1445 | 5.10E-08 | ZNF469 | novel | ALT |
| 16 | 88435292 | G | GA | 71.4539 | 10.4367 | 7.58E-12 | ZNF469 | novel | AST |
| 16 | 89541151 | A | G | 49.4653 | 9.90745 | 5.96E-07 | SPG7 | novel | GGT |
| 16 | 89541200 | A | G | 71.2484 | 11.6581 | 9.88E-10 | SPG7 | rs904356299 | GGT |
| 16 | 89919346 | C | T | 65.4021 | 9.79748 | 2.47E-11 | MC1R | rs756579024 | AP |
| 17 | 769868 | C | T | 71.8682 | 9.99917 | 6.61E-13 | GLOD4 | novel | ALT |
| 17 | 769868 | C | T | 40.8062 | 7.38113 | 3.23E-08 | GLOD4 | novel | AST |
| 17 | 771346 | C | CA | 25.7836 | 4.4159 | 5.26E-09 | GLOD4 | novel | TBIL |
| 17 | 782361 | G | A | 23.2208 | 4.41588 | 1.45E-07 | MRM3 | novel | TBIL |
| 17 | 783295 | G | A | 167.769 | 29.7212 | 1.66E-08 | MRM3 | novel | GGT |
| 17 | 783296 | G | A | 83.3013 | 14.1447 | 3.88E-09 | MRM3 | novel | ALT |
| 17 | 791863 | G | GT | 73.3564 | 11.5924 | 2.49E-10 | MRM3 | novel | AP |
| 17 | 1365056 | T | C | 113.48 | 14.1441 | 1.03E-15 | YWHAE | novel | ALT |
| 17 | 1365056 | T | C | 303.805 | 25.9185 | 1.00E-31 | YWHAE | novel | AP |
| 17 | 1365056 | T | C | 93.4756 | 10.4364 | 3.36E-19 | YWHAE | novel | AST |
| 17 | 1365056 | T | C | 551.224 | 42.0255 | 2.70E-39 | YWHAE | novel | GGT |
| 17 | 2691897 | T | G | 185.093 | 18.3281 | 5.62E-24 | CLUH | novel | AP |
| 17 | 2691897 | T | G | 17.1475 | 3.12255 | 3.99E-08 | CLUH | novel | TBIL |
| 17 | 2697895 | C | T | 211.707 | 42.0334 | 4.74E-07 | CLUH | novel | GGT |
| 17 | 3732367 | C | T | 18.1801 | 3.123 | 5.84E-09 | ITGAE | rs923781539 | TBIL |
| 17 | 6756258 | T | C | 96.4359 | 18.3287 | 1.43E-07 | XAF1 | novel | AP |
| 17 | 7010313 | C | T | 32.6546 | 5.21851 | 3.92E-10 | ALOX12 | rs776964465 | AST |
| 17 | 7223860 | T | A | 84.7586 | 10.4366 | 4.62E-16 | ACADVL | novel | AST |
| 17 | 7437297 | G | T | 23.3851 | 4.66758 | 5.44E-07 | TMEM102 | novel | AST |
| 17 | 7936483 | G | A | 229.31 | 18.8004 | 3.26E-34 | CNTROB | novel | GGT |
| 17 | 10535077 | G | A | 30.6701 | 5.29137 | 6.79E-09 | MYH2 | rs904850136 | AP |
| 17 | 12129138 | G | T | 209.035 | 42.0327 | 6.59E-07 | MAP2K4 | novel | GGT |
| 17 | 16381909 | T | C | 24.0789 | 4.41623 | 4.97E-08 | UBB | novel | TBIL |
| 17 | 16382299 | C | T | 136.487 | 24.2677 | 1.86E-08 | UBB | novel | GGT |
| 17 | 17205932 | G | A | 253.734 | 42.0316 | 1.57E-09 | PLD6 | novel | GGT |
| 17 | 17797386 | A | G | 2.59472 | 0.519773 | 5.98E-07 | RAI1 | rs139684843 | AST |
| 17 | 17836817 | T | C | 51.0126 | 8.19726 | 4.88E-10 | SREBF1 | novel | AP |
| 17 | 18290841 | C | T | 509.962 | 42.0262 | 7.04E-34 | TOP3A | novel | GGT |
| 17 | 18333240 | T | TAAAAG | 58.8793 | 6.02529 | 1.49E-22 | SHMT1 | rs865866988 | AST |
| 17 | 18340242 | G | GTA | 241.052 | 29.7205 | 5.05E-16 | SHMT1 | novel | GGT |
| 17 | 18348345 | A | AC | 25.8862 | 4.66863 | 2.95E-08 | SHMT1 | rs756737767 | AST |
| 17 | 19739074 | C | CT | 36.9401 | 7.38002 | 5.58E-07 | ALDH3A1 | novel | AST |
| 17 | 21298927 | G | A | 150.772 | 25.9212 | 6.01E-09 | MAP2K3 | rs762048822 | AP |
| 17 | 28372273 | C | T | 173.632 | 24.2667 | 8.37E-13 | SARM1 | novel | GGT |
| 17 | 29616079 | C | T | 12.8292 | 2.54954 | 4.86E-07 | CORO6 | novel | TBIL |
| 17 | 30904361 | G | C | 283.556 | 42.0312 | 1.52E-11 | TEFM | novel | GGT |
| 17 | 36602354 | C | A | 92.2195 | 14.1455 | 7.07E-11 | MRM1 | novel | ALT |
| 17 | 38764156 | C | G | 45.0348 | 8.16636 | 3.50E-08 | PSMB3 | rs764625412 | ALT |
| 17 | 39657086 | G | T | 61.2455 | 8.16794 | 6.48E-14 | STARD3 | novel | ALT |
| 17 | 39661021 | C | T | 145.361 | 25.9221 | 2.05E-08 | STARD3 | rs755347541 | AP |
| 17 | 39661021 | C | T | 1149.68 | 41.9972 | 7.58E-165 | STARD3 | rs755347541 | GGT |
| 17 | 40412875 | T | TA | 179.255 | 14.1433 | 8.34E-37 | TOP2A | novel | ALT |
| 17 | 40412875 | T | TA | 152.269 | 25.923 | 4.26E-09 | TOP2A | novel | AP |
| 17 | 40412875 | T | TA | 201.162 | 10.4333 | 8.47E-83 | TOP2A | novel | AST |
| 17 | 41727064 | A | C | 25.9101 | 4.41624 | 4.44E-09 | HAP1 | novel | TBIL |
| 17 | 41732693 | G | A | 484.143 | 42.0237 | 1.05E-30 | HAP1 | novel | GGT |
| 17 | 41821819 | T | C | 52.9123 | 10.437 | 3.99E-07 | FKBP10 | novel | AST |
| 17 | 41821819 | T | C | 579.869 | 42.0242 | 2.66E-43 | FKBP10 | novel | GGT |
| 17 | 42223440 | C | CT | 28.8219 | 4.41586 | 6.72E-11 | STAT5B | novel | TBIL |
| 17 | 43200424 | C | T | 43.732 | 4.41553 | 4.02E-23 | NBR1 | novel | TBIL |
| 17 | 44014363 | C | T | 21.911 | 4.41595 | 6.99E-07 | TMEM101 | novel | TBIL |
| 17 | 44014983 | C | A | 30.9979 | 4.41579 | 2.23E-12 | TMEM101 | novel | TBIL |
| 17 | 44351663 | A | AG | 141.965 | 14.1434 | 1.05E-23 | GRN | novel | ALT |
| 17 | 44351663 | A | AG | 75.5836 | 10.4367 | 4.43E-13 | GRN | novel | AST |
| 17 | 44914110 | C | T | 396.715 | 29.7164 | 1.21E-40 | GFAP | novel | GGT |
| 17 | 45962396 | T | TG | 51.8614 | 10.4369 | 6.73E-07 | MAPT | novel | AST |
| 17 | 45983351 | C | CA | 260.403 | 42.0317 | 5.82E-10 | MAPT | novel | GGT |
| 17 | 48893429 | C | T | 15.447 | 2.2079 | 2.63E-12 | ATP5MC1 | novel | TBIL |
| 17 | 49512004 | C | T | 54.8288 | 10.4369 | 1.49E-07 | NGFR | novel | AST |
| 17 | 50385262 | G | A | 48.2324 | 8.16636 | 3.50E-09 | LRRC59 | novel | ALT |
| 17 | 50426263 | T | C | 52.718 | 7.18925 | 2.26E-13 | ACSF2 | novel | AP |
| 17 | 50548453 | G | A | 47.5145 | 7.37992 | 1.21E-10 | SPATA20 | novel | AST |
| 17 | 50548453 | G | A | 204.314 | 29.7206 | 6.23E-12 | SPATA20 | novel | GGT |
| 17 | 57105879 | G | T | 30.4814 | 4.41578 | 5.10E-12 | AKAP1 | novel | TBIL |
| 17 | 59220753 | T | C | 28.7254 | 4.4158 | 7.77E-11 | GDPD1 | novel | TBIL |
| 17 | 59685701 | T | TA | 160.581 | 25.9213 | 5.84E-10 | CLTC | novel | AP |
| 17 | 59685701 | T | TA | 214.99 | 42.0323 | 3.14E-07 | CLTC | novel | GGT |
| 17 | 60043787 | G | A | 73.7878 | 10.0026 | 1.62E-13 | HEATR6 | novel | ALT |
| 17 | 64068245 | C | T | 294.75 | 25.9185 | 5.81E-30 | ERN1 | novel | AP |
| 17 | 64068245 | C | T | 661.277 | 42.0212 | 8.79E-56 | ERN1 | novel | GGT |
| 17 | 64072104 | C | T | 148.078 | 29.7214 | 6.29E-07 | ERN1 | novel | GGT |
| 17 | 64080773 | A | C | 73.1903 | 14.1444 | 2.29E-07 | ERN1 | novel | ALT |
| 17 | 64080773 | A | C | 66.0285 | 10.4376 | 2.52E-10 | ERN1 | novel | AST |
| 17 | 68436442 | G | C | 130.563 | 14.144 | 2.69E-20 | WIPI1 | novel | ALT |
| 17 | 68436442 | G | C | 96.1644 | 10.4365 | 3.15E-20 | WIPI1 | novel | AST |
| 17 | 68990835 | A | G | 334.641 | 42.0304 | 1.70E-15 | ABCA9 | novel | GGT |
| 17 | 69021829 | G | A | 231.769 | 42.0333 | 3.51E-08 | ABCA9 | novel | GGT |
| 17 | 69029168 | C | A | 94.1424 | 14.1454 | 2.83E-11 | ABCA9 | rs751003002 | ALT |
| 17 | 69051042 | G | A | 176.606 | 25.9208 | 9.55E-12 | ABCA9 | novel | AP |
| 17 | 69051042 | G | A | 239.962 | 42.0321 | 1.14E-08 | ABCA9 | novel | GGT |
| 17 | 74872940 | G | GCCAT | 101.045 | 18.7975 | 7.64E-08 | FDXR | novel | GGT |
| 17 | 75019737 | T | G | 112.866 | 18.3294 | 7.39E-10 | MRPL58 | novel | AP |
| 17 | 75020312 | G | C | 110.535 | 10.0008 | 2.15E-28 | MRPL58 | novel | ALT |
| 17 | 75020312 | G | C | 250.08 | 18.326 | 2.17E-42 | MRPL58 | novel | AP |
| 17 | 75020312 | G | C | 58.6704 | 7.37952 | 1.86E-15 | MRPL58 | novel | AST |
| 17 | 75020312 | G | C | 149.599 | 29.7217 | 4.82E-07 | MRPL58 | novel | GGT |
| 17 | 75038934 | A | G | 22.655 | 4.41591 | 2.89E-07 | ATP5PD | novel | TBIL |
| 17 | 78378666 | A | ATGGCGG | 178.705 | 21.0151 | 1.84E-17 | PGS1 | rs767269824 | GGT |
| 17 | 78400840 | G | A | 194.211 | 17.1577 | 1.07E-29 | PGS1 | novel | GGT |
| 17 | 81665336 | G | A | 102.888 | 18.807 | 4.48E-08 | OXLD1 | rs368222035 | GGT |
| 17 | 81833853 | C | T | 8.28887 | 1.3965 | 2.93E-09 | PPP1R27 | rs752939473 | TBIL |
| 17 | 81845956 | C | T | 64.533 | 10.4368 | 6.29E-10 | P4HB | novel | AST |
| 17 | 81914142 | C | T | 15.8107 | 3.12251 | 4.12E-07 | SIRT7 | rs201841580 | TBIL |
| 17 | 82236232 | G | A | 24.0452 | 4.4159 | 5.18E-08 | SLC16A3 | novel | TBIL |
| 17 | 82619074 | G | A | 19.6211 | 3.12249 | 3.31E-10 | WDR45B | rs786205510 | TBIL |
| 18 | 10548379 | G | A | 71.7637 | 14.1449 | 3.91E-07 | NAPG | novel | ALT |
| 18 | 10548379 | G | A | 198.202 | 25.921 | 2.07E-14 | NAPG | novel | AP |
| 18 | 10548379 | G | A | 69.9298 | 10.4369 | 2.08E-11 | NAPG | novel | AST |
| 18 | 10548379 | G | A | 354.137 | 42.031 | 3.60E-17 | NAPG | novel | GGT |
| 18 | 12420324 | G | A | 986.936 | 42.0062 | 5.50E-122 | PRELID3A | novel | GGT |
| 18 | 23536743 | G | A | 108.7 | 21.0166 | 2.32E-07 | NPC1 | rs786204455 | GGT |
| 18 | 23539354 | C | T | 51.8328 | 8.16749 | 2.21E-10 | NPC1 | novel | ALT |
| 18 | 23541127 | G | T | 15.724 | 2.46004 | 1.64E-10 | NPC1 | novel | AST |
| 18 | 42015475 | G | A | 98.7639 | 10.0013 | 5.37E-23 | PIK3C3 | novel | ALT |
| 18 | 42015475 | G | A | 73.8309 | 7.37976 | 1.46E-23 | PIK3C3 | novel | AST |
| 18 | 50918172 | T | A | 172.989 | 25.9221 | 2.50E-11 | ME2 | novel | AP |
| 18 | 50918172 | T | A | 75.1589 | 10.4366 | 5.97E-13 | ME2 | novel | AST |
| 18 | 50918172 | T | A | 280.181 | 42.0293 | 2.63E-11 | ME2 | novel | GGT |
| 18 | 55401499 | C | T | 152.989 | 29.7245 | 2.65E-07 | TCF4 | novel | GGT |
| 18 | 59345970 | T | C | 54.1637 | 9.39917 | 8.29E-09 | LMAN1 | rs183873209 | GGT |
| 18 | 77017007 | A | G | 187.997 | 29.7211 | 2.53E-10 | MBP | novel | GGT |
| 19 | 582541 | C | G | 73.0525 | 14.1446 | 2.41E-07 | BSG | novel | ALT |
| 19 | 632902 | G | T | 143.996 | 21.0158 | 7.30E-12 | POLRMT | novel | GGT |
| 19 | 3978026 | G | A | 46.8087 | 7.37995 | 2.26E-10 | EEF2 | rs781090546 | AST |
| 19 | 5712049 | G | GCTGCC | 42.2037 | 6.02625 | 2.50E-12 | LONP1 | rs538821177 | AST |
| 19 | 7850042 | G | T | 56.4409 | 10.4369 | 6.38E-08 | EVI5L | novel | AST |
| 19 | 10687709 | G | A | 25.8143 | 4.41586 | 5.04E-09 | ILF3 | rs113804117 | TBIL |
| 19 | 10829221 | A | AC | 75.2294 | 14.1446 | 1.05E-07 | DNM2 | novel | ALT |
| 19 | 10929387 | G | A | 78.9729 | 14.1446 | 2.36E-08 | TIMM29 | novel | ALT |
| 19 | 11113544 | T | TGACA | 22.1676 | 4.4159 | 5.17E-07 | LDLR | rs875989920 | TBIL |
| 19 | 12896422 | G | C | 401.217 | 42.0295 | 1.35E-21 | GCDH | novel | GGT |
| 19 | 14566916 | G | A | 13.8008 | 2.20795 | 4.10E-10 | NDUFB7 | novel | TBIL |
| 19 | 15120082 | C | T | 192.075 | 9.99818 | 3.25E-82 | ILVBL | novel | ALT |
| 19 | 15120082 | C | T | 144.545 | 7.37727 | 1.93E-85 | ILVBL | novel | AST |
| 19 | 15376975 | C | T | 13.2166 | 2.20793 | 2.15E-09 | AKAP8 | novel | TBIL |
| 19 | 16157788 | C | A | 39.4546 | 7.67456 | 2.73E-07 | HSH2D | novel | GGT |
| 19 | 17306366 | C | A | 110.566 | 14.9655 | 1.49E-13 | MRPL34 | novel | AP |
| 19 | 17337933 | C | A | 155.585 | 25.9232 | 1.95E-09 | GTPBP3 | novel | AP |
| 19 | 17338572 | G | A | 73.5377 | 14.1446 | 2.00E-07 | GTPBP3 | rs769418711 | ALT |
| 19 | 17338572 | G | A | 141.459 | 25.9215 | 4.84E-08 | GTPBP3 | rs769418711 | AP |
| 19 | 17338572 | G | A | 754.647 | 42.0174 | 4.24E-72 | GTPBP3 | rs769418711 | GGT |
| 19 | 17500743 | C | A | 93.0569 | 14.1443 | 4.74E-11 | SLC27A1 | novel | ALT |
| 19 | 17500743 | C | A | 186.031 | 25.9208 | 7.14E-13 | SLC27A1 | novel | AP |
| 19 | 17500743 | C | A | 100.234 | 10.4361 | 7.69E-22 | SLC27A1 | novel | AST |
| 19 | 17500743 | C | A | 711.933 | 42.0192 | 2.28E-64 | SLC27A1 | novel | GGT |
| 19 | 17500743 | C | A | 45.8317 | 4.41546 | 3.09E-25 | SLC27A1 | novel | TBIL |
| 19 | 17720357 | A | G | 112.682 | 14.144 | 1.63E-15 | MAP1S | novel | ALT |
| 19 | 17720357 | A | G | 66.883 | 10.4368 | 1.47E-10 | MAP1S | novel | AST |
| 19 | 17727143 | C | T | 103.857 | 14.1443 | 2.10E-13 | MAP1S | rs770051124 | ALT |
| 19 | 17734390 | C | T | 77.1265 | 10.0025 | 1.25E-14 | MAP1S | novel | ALT |
| 19 | 30011136 | G | T | 417.854 | 42.0286 | 2.75E-23 | URI1 | novel | GGT |
| 19 | 32915458 | C | A | -5.20534 | 1.05489 | 8.04E-07 | CEP89 | rs770853408 | ALB |
| 19 | 38315723 | C | CT | 70.0207 | 14.1446 | 7.41E-07 | YIF1B | rs769816316 | ALT |
| 19 | 38490106 | C | T | 24.8279 | 4.41588 | 1.88E-08 | RYR1 | novel | TBIL |
| 19 | 38502726 | A | C | 401.248 | 41.8985 | 1.01E-21 | RYR1 | novel | GGT |
| 19 | 38527672 | G | A | 74.7911 | 10.0027 | 7.61E-14 | RYR1 | novel | ALT |
| 19 | 38916035 | A | G | 70.8457 | 4.25964 | 4.29E-62 | SARS2 | novel | AST |
| 19 | 38917788 | G | A | 216.152 | 42.0325 | 2.71E-07 | SARS2 | novel | GGT |
| 19 | 42405475 | G | A | 67.4423 | 11.658 | 7.25E-09 | LIPE | rs377459162 | GGT |
| 19 | 44906639 | G | A | 462.191 | 42.0302 | 4.00E-28 | APOE | rs777551553 | GGT |
| 19 | 45348911 | T | A | 234.383 | 42.1277 | 2.64E-08 | KLC3 | novel | GGT |
| 19 | 48800102 | T | C | 267.385 | 42.0301 | 2.00E-10 | BCAT2 | novel | GGT |
| 19 | 48965600 | C | T | 41.3542 | 5.77444 | 7.99E-13 | FTL | novel | ALT |
| 19 | 48965600 | C | T | 26.7592 | 4.26072 | 3.38E-10 | FTL | novel | AST |
| 19 | 48965600 | C | T | 200.156 | 17.1573 | 1.92E-31 | FTL | novel | GGT |
| 19 | 48966666 | C | G | 53.7965 | 8.16625 | 4.47E-11 | FTL | novel | ALT |
| 19 | 48966666 | C | G | 48.9459 | 6.0255 | 4.56E-16 | FTL | novel | AST |
| 19 | 50250602 | C | T | 329.924 | 42.0307 | 4.18E-15 | MYH14 | novel | GGT |
| 19 | 50250689 | G | A | 146.763 | 29.7217 | 7.90E-07 | MYH14 | novel | GGT |
| 20 | 2659794 | C | T | 68.2782 | 6.02615 | 9.38E-30 | IDH3B | novel | AST |
| 20 | 3148071 | C | G | 101.871 | 17.1595 | 2.91E-09 | FASTKD5 | novel | GGT |
| 20 | 3861330 | A | G | 29.5783 | 4.41582 | 2.11E-11 | MAVS | novel | TBIL |
| 20 | 5183100 | C | T | 117.657 | 14.1439 | 8.93E-17 | CDS2 | novel | ALT |
| 20 | 5183100 | C | T | 73.8733 | 10.4367 | 1.46E-12 | CDS2 | novel | AST |
| 20 | 6031330 | C | CTGCTG | 55.2224 | 10.4374 | 1.22E-07 | CRLS1 | rs756040375 | AST |
| 20 | 9544367 | A | C | 85.7581 | 14.1445 | 1.34E-09 | PAK5 | novel | ALT |
| 20 | 17494595 | C | CT | 209.796 | 42.0322 | 6.00E-07 | BFSP1 | rs759229724 | GGT |
| 20 | 25057798 | C | T | 129.659 | 15.8862 | 3.31E-16 | ACSS1 | novel | GGT |
| 20 | 31643503 | C | CA | 73.2433 | 14.165 | 2.33E-07 | COX4I2 | rs766687519 | ALT |
| 20 | 31643503 | C | CA | 265.233 | 42.0921 | 2.96E-10 | COX4I2 | rs766687519 | GGT |
| 20 | 33374155 | G | A | 148.918 | 29.7241 | 5.44E-07 | CDK5RAP1 | rs775021762 | GGT |
| 20 | 34946052 | C | T | 52.3372 | 10.0019 | 1.67E-07 | GSS | novel | ALT |
| 20 | 35548847 | C | T | 24.5338 | 4.41587 | 2.76E-08 | ERGIC3 | novel | TBIL |
| 20 | 44406134 | G | C | 34.0696 | 6.6931 | 3.58E-07 | HNF4A | rs781364773 | AP |
| 20 | 46010921 | G | A | 149.31 | 24.2671 | 7.62E-10 | MMP9 | novel | GGT |
| 20 | 46011572 | A | G | 941.904 | 42.0084 | 2.80E-111 | MMP9 | rs774152169 | GGT |
| 20 | 54157199 | T | C | 16.0391 | 2.08708 | 1.53E-14 | CYP24A1 | novel | AST |
| 20 | 57564168 | G | C | 12.8759 | 1.93815 | 3.07E-11 | PCK1 | rs773173117 | AST |
| 20 | 57565606 | C | G | 9.0614 | 1.52249 | 2.66E-09 | PCK1 | rs371151851 | AST |
| 20 | 63567648 | C | T | 96.2112 | 10.002 | 6.67E-22 | HELZ2 | novel | ALT |
| 20 | 63567648 | C | T | 225.351 | 29.7233 | 3.42E-14 | HELZ2 | novel | GGT |
| 21 | 26000035 | A | AT | 222.749 | 42.0322 | 1.16E-07 | APP | novel | GGT |
| 21 | 36045897 | A | T | 77.7559 | 14.1446 | 3.86E-08 | SETD4 | novel | ALT |
| 21 | 36045897 | A | T | 87.9314 | 10.4364 | 3.61E-17 | SETD4 | novel | AST |
| 21 | 36146471 | C | T | 51.325 | 10.0018 | 2.87E-07 | CBR3 | novel | ALT |
| 21 | 37088194 | A | G | 147.675 | 24.2674 | 1.16E-09 | TTC3 | novel | GGT |
| 21 | 42904261 | C | T | 37.7943 | 7.31853 | 2.42E-07 | NDUFV3 | rs368251445 | GGT |
| 21 | 44426692 | C | G | 117.975 | 17.1595 | 6.20E-12 | TRPM2 | novel | GGT |
| 21 | 46210719 | T | TC | 52.2925 | 10.002 | 1.71E-07 | LSS | novel | ALT |
| 22 | 19178166 | C | A | 43.5373 | 7.07221 | 7.46E-10 | SLC25A1 | rs868977488 | ALT |
| 22 | 19178166 | C | A | 117.962 | 21.0163 | 1.99E-08 | SLC25A1 | rs868977488 | GGT |
| 22 | 20140615 | A | G | 153.413 | 25.9214 | 3.25E-09 | ZDHHC8 | novel | AP |
| 22 | 20712485 | C | T | 267.425 | 42.0318 | 1.99E-10 | PI4KA | novel | GGT |
| 22 | 24070798 | A | G | 26.1051 | 4.41584 | 3.39E-09 | CABIN1 | novel | TBIL |
| 22 | 24440863 | C | T | 17.4462 | 3.12256 | 2.31E-08 | ADORA2A | novel | TBIL |
| 22 | 29480917 | A | T | 196.976 | 24.2663 | 4.78E-16 | NEFH | novel | GGT |
| 22 | 32484087 | T | TA | 217.591 | 42.0328 | 2.26E-07 | FBXO7 | novel | GGT |
| 22 | 36305099 | A | G | 132.607 | 18.8026 | 1.76E-12 | MYH9 | rs990176353 | GGT |
| 22 | 37018331 | C | T | 18.3774 | 3.1225 | 3.97E-09 | TST | rs769952949 | TBIL |
| 22 | 39522429 | T | G | 24.8295 | 4.41587 | 1.88E-08 | ATF4 | novel | TBIL |
| 22 | 40779212 | A | G | 159.54 | 29.7211 | 7.97E-08 | SLC25A17 | novel | GGT |
| 22 | 40860707 | C | T | 65.9125 | 10.0005 | 4.38E-11 | XPNPEP3 | novel | ALT |
| 22 | 41526447 | C | A | 213.714 | 42.0322 | 3.69E-07 | ACO2 | novel | GGT |
| 22 | 41875614 | C | T | 343.36 | 42.0304 | 3.11E-16 | SREBF2 | novel | GGT |
| 22 | 46356913 | C | A | 58.8071 | 10.0017 | 4.11E-09 | TRMU | novel | ALT |
| 22 | 46356913 | C | A | 37.5837 | 7.38005 | 3.53E-07 | TRMU | novel | AST |
| 22 | 46687207 | C | T | 27.5349 | 4.41587 | 4.51E-10 | CERK | novel | TBIL |
| 22 | 50255195 | A | G | 25.2137 | 4.4159 | 1.13E-08 | MAPK12 | novel | TBIL |
| 22 | 50261229 | C | G | 11.1948 | 2.20826 | 3.99E-07 | MAPK12 | novel | TBIL |
| 22 | 50261501 | A | AGAGCTCATGGCAGGCCCGG | 27.2358 | 4.41746 | 7.03E-10 | MAPK12 | novel | TBIL |
| 22 | 50524144 | G | A | 24.844 | 4.26485 | 5.71E-09 | SCO2 | rs74315512 | ALT |
| 22 | 50524144 | G | A | 16.5306 | 3.14694 | 1.50E-07 | SCO2 | rs74315512 | AST |
| 23 | 2221230 | C | G | 229.95 | 13.6828 | 2.32E-63 | DHRSX | novel | ALT |
| 23 | 2221230 | C | G | 228.018 | 25.8763 | 1.24E-18 | DHRSX | novel | AP |
| 23 | 2221230 | C | G | 126.283 | 10.2739 | 1.02E-34 | DHRSX | novel | AST |
| 23 | 2221230 | C | G | 544.767 | 41.3102 | 1.06E-39 | DHRSX | novel | GGT |
| 23 | 23783730 | C | CG | 906.776 | 41.2834 | 7.20E-107 | SAT1 | novel | GGT |
| 23 | 23783730 | C | CG | 34.0329 | 4.28127 | 1.88E-15 | SAT1 | novel | TBIL |
| 23 | 31134135 | C | A | 73.913 | 10.2752 | 6.33E-13 | DMD | novel | AST |
| 23 | 32844794 | G | A | 24.4793 | 4.28105 | 1.08E-08 | DMD | rs128626234 | TBIL |
| 23 | 47571520 | G | T | 46.0386 | 3.42311 | 3.17E-41 | ARAF | novel | AST |
| 23 | 47571520 | G | T | 196.532 | 13.7688 | 3.27E-46 | ARAF | novel | GGT |
| 23 | 91436723 | C | A | 94.9753 | 18.2987 | 2.10E-07 | PABPC5 | rs369864267 | AP |
| 23 | 91436723 | C | A | 296.094 | 29.214 | 3.87E-24 | PABPC5 | rs369864267 | GGT |
| 23 | 100874113 | C | T | 19.6904 | 3.65811 | 7.34E-08 | NOX1 | novel | ALT |
| 23 | 101010708 | C | T | 38.7226 | 5.93678 | 6.92E-11 | TRMT2B | novel | AP |
| 23 | 101010708 | C | T | 69.2635 | 9.47875 | 2.73E-13 | TRMT2B | novel | GGT |
| 23 | 101656607 | C | T | 17.6503 | 2.141 | 1.67E-16 | ARMCX2 | novel | TBIL |
| 23 | 130340354 | G | A | 10.5586 | 2.14083 | 8.14E-07 | SLC25A14 | novel | TBIL |
| 23 | 155064035 | T | C | 16.8763 | 3.02722 | 2.48E-08 | CMC4 | rs782029945 | TBIL |

# Figure S1 Functional enrichment analysis of significantly associated PTVs for each liver-related biomarker.

**A**, the pathways of Kyoto Encyclopedia of Genes and Genomes pathway analysis **(**KEGG) based on PTVs linked to each liver-related biomarker. **B**–**D**, the terminologies of biological process (BP) (**B**), cellular component (CC) (**C**), and molecular function (MF) (**D**) in gene ontology (GO) analysis based on PTVs linked to each liver-related biomarker. Abbreviation: PPAR, peroxisome proliferator-activated receptor; ABC, ATP-binding cassette; MT, mitochondrial; NADP, nicotinamide adenine dinucleotide phosphate.


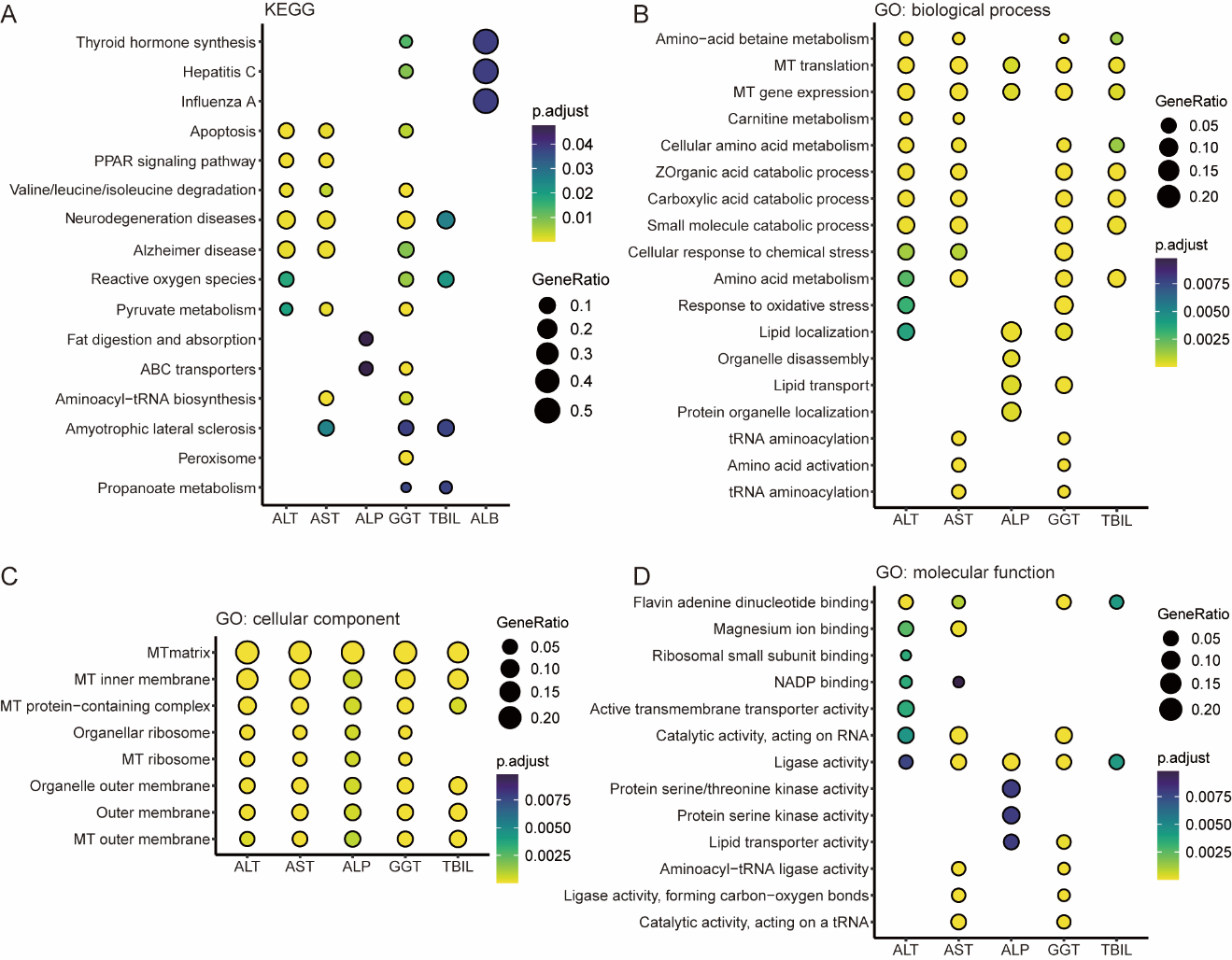


# Figure S2 Impact of biomarker-specific and pathway-specific PTVs on the risks of end-stage liver diseases and liver-related mortality.

The impact of PTVs on the risk of end-stage liver diseases (fibrosis and cirrhosis of liver and liver cancer) and liver disease-related mortality was evaluated using a COX regression model. The model was adjusted for age, sex, alcohol intake frequency, ever smoked, physical activity level, education deprivation score, household income, BMI, hypertension, diabetes, and dyslipidemia. The incidence rates (per 100,000-person years, /10^5pys) of diseases were also calculated in the whole population and subgroups. We report here only the results of PTVs with statistically significant. Abbreviation: PTV, protein truncating variant; HR, hazard ratio; CI, confidence interval.


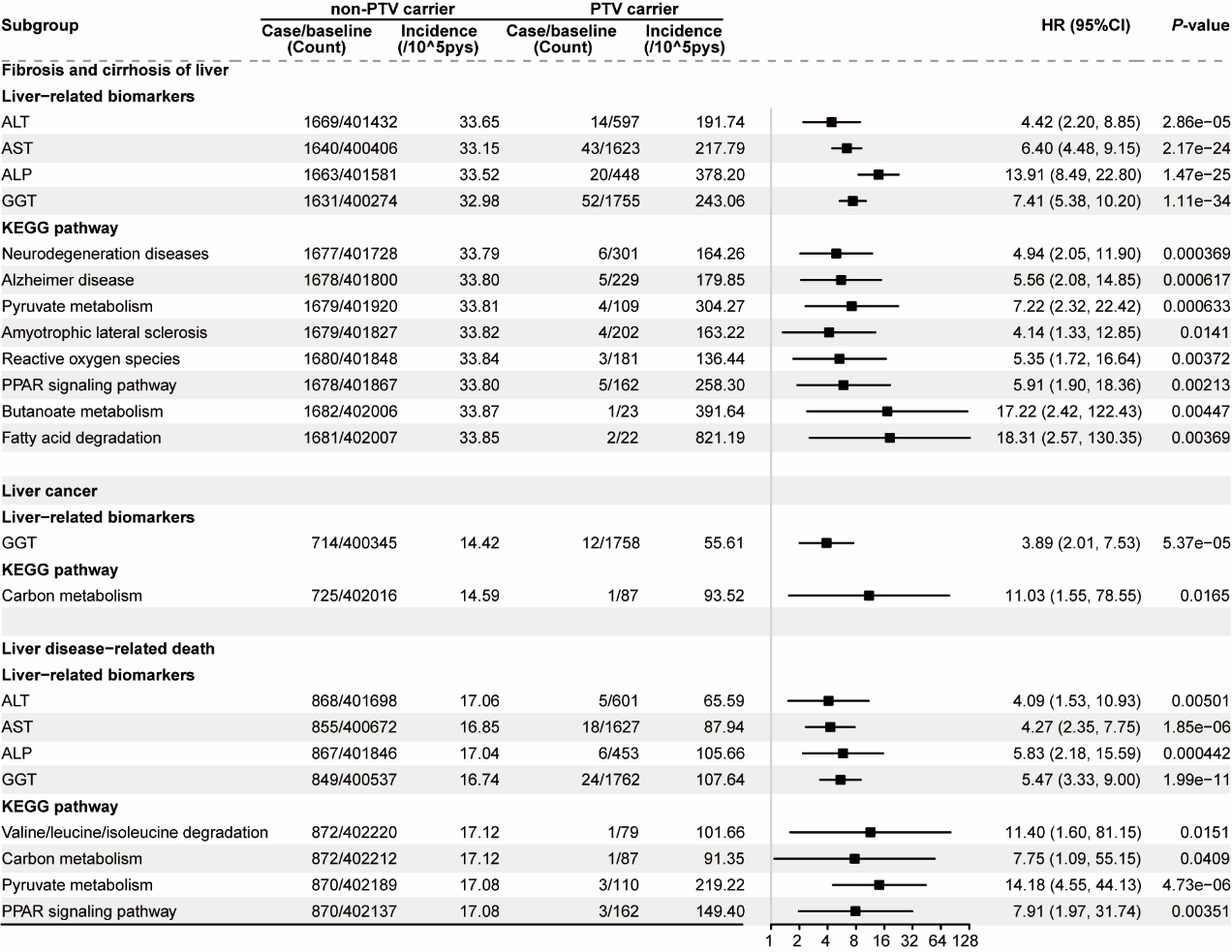


# Figure S3 Impact of PTVs on the risks of non-alcoholic fatty liver disease, viral hepatitis, and autoimmune hepatitis.

The impact of PTVs on the risk of non-alcoholic fatty liver disease, viral hepatitis, and autoimmune hepatitis was evaluated using a COX regression model. The model was adjusted for age, sex, alcohol intake frequency, ever smoked, physical activity level, education deprivation score, household income, BMI, hypertension, diabetes, and dyslipidemia. The incidence rates (per 100,000-person years, /10^5pys) of diseases were also calculated in the whole population and subgroups. We report here only the results of PTVs with statistically significant. Abbreviation: PTV, protein truncating variant; HR, hazard ratio; CI, confidence interval; NAFLD, non-alcoholic fatty liver disease.


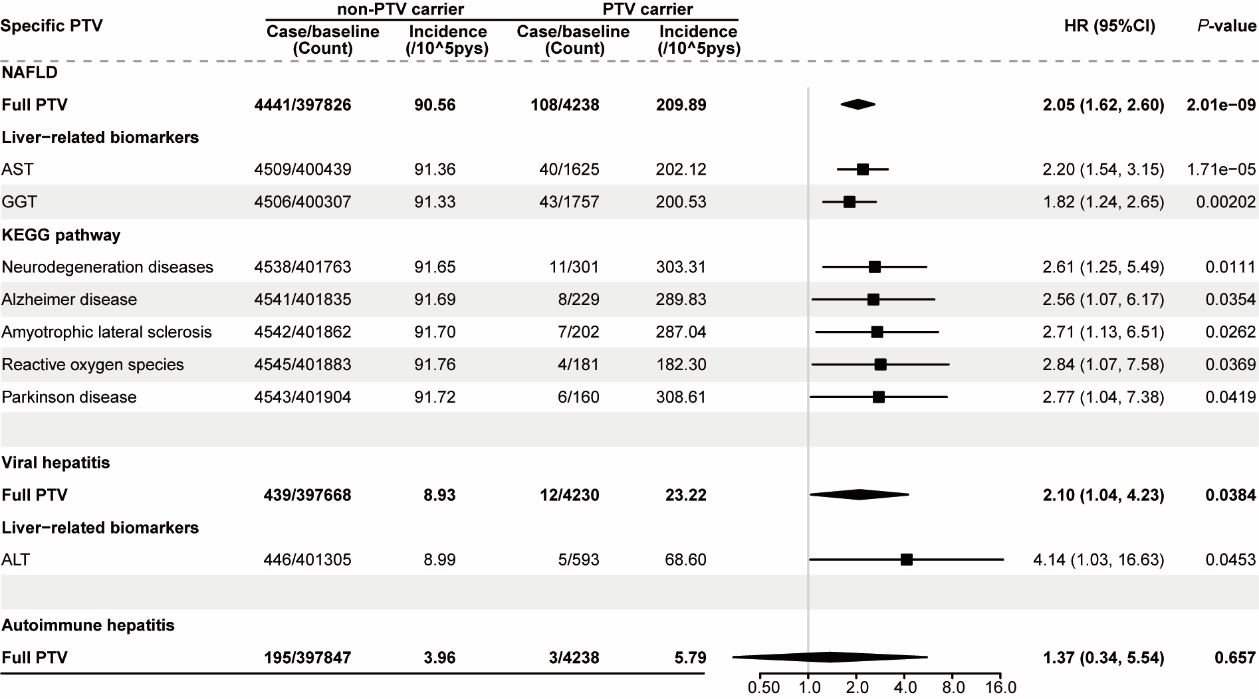


# Figure S4 Stratified analysis of the impact of PTVs on mitochondrial-related PTVs on the risks of end-stage liver diseases and liver-related mortality based on liver disease susceptibility genes.

The impact of PTVs on the risk of end-stage liver diseases (fibrosis and cirrhosis of liver and liver cancer) and liver disease-related mortality was evaluated using a COX regression model. The model was adjusted for age, sex, alcohol intake frequency, ever smoked, physical activity level, education deprivation score, household income, BMI, hypertension, diabetes, and dyslipidemia. The incidence rates (per 100,000-person years, /10^5pys) of diseases were also calculated in the whole population and subgroups. We report here only the results of PTVs with statistically significant. Abbreviation: PTV, protein truncating variant; HR, hazard ratio; CI, confidence interval.


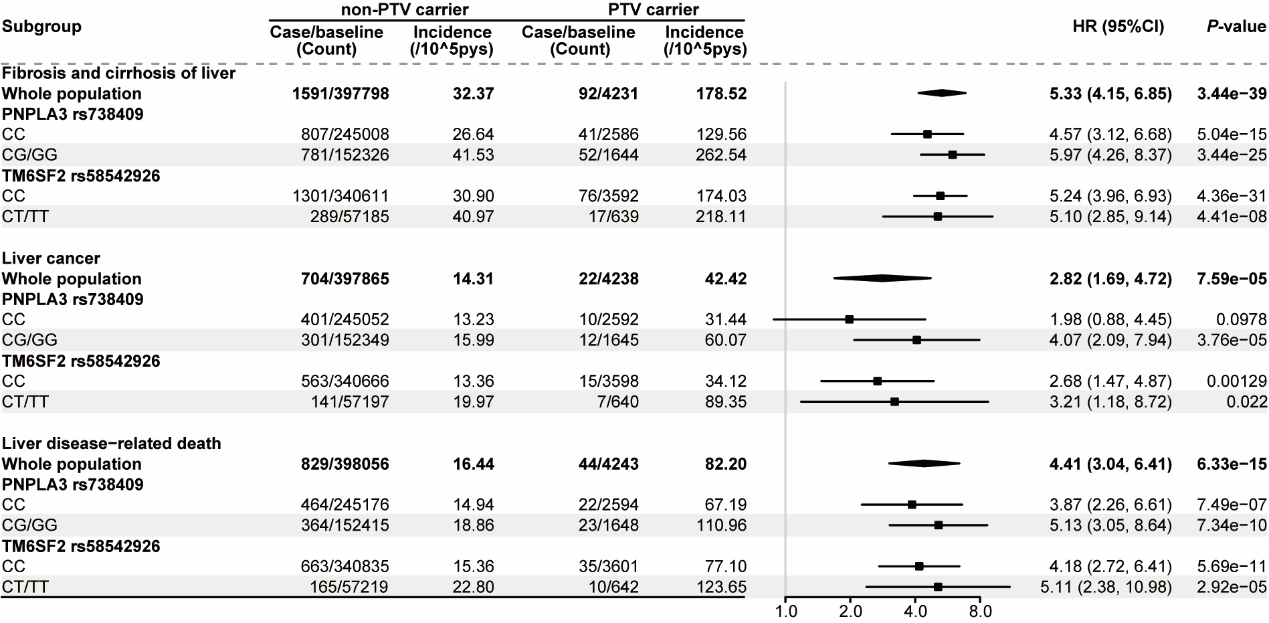

Supplement: Supplementary file 1 — Additional file 1: Table S1 List of mitochondrial-related nuclear genes in the study. Table S2 Mitochondrial-related protein truncating variants that were positively associated with liver dysfunction. Fig. S1 Functional enrichment analysis of significantly associated PTVs for each liver-related biomarker. Fig. S2 Impact of biomarker-specific and pathway-specific PTVs on the risks of end-stage liver diseases and liver-related mortality. Fig. S3 Impact of PTVs on the risks of non-alcoholic fatty liver disease, viral hepatitis, and autoimmune hepatitis. Fig. S4 Stratified analysis of the impact of PTVs on mitochondrial-related PTVs on the risks of end-stage liver diseases and liver-related mortality based on liver disease susceptibility genes. [file 12916_2024_3466_MOESM1_ESM.docx]
